# Targeting malaria in high-risk populations in low endemic regions in northern Namibia: a quasi-experimental controlled trial to reduce malaria in seasonal agricultural workers and cattle herders

**DOI:** 10.1136/bmjgh-2024-015565

**Published:** 2025-02-17

**Authors:** Jennifer L Smith, Henry Ntuku, Francois Rerolle, Ashley Morgan Burke, Tabeth Mwema, Keirstinne Turcios, Petrina Uusiku, Justine Kulla Haikali, Michael Lifasi, Cara Smith-Gueye, Elodie Vajda, Jerry O Jacobson, Bryan Greenhouse, Roly Gosling, Adam Bennett, Davis R Mumbengegwi

**Affiliations:** 1 Epidemiology & Biostatistics, University of California San Francisco, San Francisco, California, USA; 2 Malaria Elimination Initiative, University of California San Francisco, San Francisco, California, USA; 3 University of the Witwatersrand Johannesburg Faculty of Health Sciences, Johannesburg, Gauteng, South Africa; 4 National Institute for Communicable Diseases, Johannesburg, South Africa; 5 Multidisciplinary Research Services, University of Namibia, Windhoek, Namibia; 6 Department of Medicine, University of California San Francisco, San Francisco, California, USA; 7 National Vector-Borne Diseases Control Program, Namibia Ministry of Health and Social Services, Windhoek, Khomas, Namibia; 8 Ohangwena Health Directorate, Republic of Namibia Ministry of Health and Social Services, Windhoek, Khomas, Namibia; 9 Zambezi Health Directorate, Republic of Namibia Ministry of Health and Social Services, Windhoek, Khomas, Namibia; 10 Malaria Operational Research Program, Multidisciplinary Research Services, University of Namibia, Windhoek, Khomas, Namibia

**Keywords:** malaria, intervention study, epidemiology, global health, medical entomology

## Abstract

**Background:**

Agricultural worksites are rarely targeted by malaria control programmes, yet may play a role in maintaining local transmission due to workers’ high mobility, low intervention coverage and occupational exposures.

**Methods:**

A quasi-experimental controlled intervention study was carried out in farming and cattle herding populations in northern Namibia to evaluate the impact of a targeted malaria intervention package. Eight health facility catchment areas in Zambezi and Ohangwena Regions were randomised to an intervention arm and eligible individuals within worksites in intervention areas received targeted drug administration with artemether-lumefantrine, mop-up indoor residual spraying and long-lasting insecticidal nets, combined with distribution of topical repellent in Zambezi Region. Impact on malaria outcomes and intervention coverage was evaluated over a single transmission season using pre-intervention and post-intervention cross-sectional surveys in a random subset of worksites and community incidence from passively detected cases. Entomological collections and residual efficacy assays on canvas and tarpaulin were conducted.

**Results:**

Delivery of a single intervention round was associated with a reduction in the prevalence of malaria (OR 0.24, 95% CI 0.1 to 0.5; risk difference (RD) −6.0%, 95% CI −9.4 to –2.8). Coverage of at least one intervention increased (RD 51.6%, 95% CI 44.4 to 58.2) among the target population in intervention compared with control areas. This effect was largely driven by results in Zambezi Region, which also observed a decline in community incidence (−1.29 cases/1000 person-weeks, 95% CI −2.2 to –0.3). Residual efficacy of pirimiphos-methyl (Actellic) on tarpaulin and canvas was high at 24hours but declined to 44.6% at 4 months.

**Conclusion:**

The study shows that targeted delivery of malaria interventions to cattle herders and agricultural workers at worksites has potential to impact local transmission. Findings highlight the need for further research on the role of key populations in *Plasmodium falciparum* transmission in Namibia.

**Trial registration number:**

NCT04094727.

**WHAT IS ALREADY KNOWN ON THIS TOPIC**

- Seasonal agricultural workers are key populations at higher risk of malaria who are mobile and frequently missed by routine interventions.

**WHAT THIS STUDY ADDS**

- The study illustrates how a community-based approach, with malaria programmes, employers and community leaders, can improve access and delivery of malaria interventions and reduce transmission in a key population.
- Findings also suggest possible spillover effects to communities and low residual efficacy of insecticides; however, more research is needed to demonstrate the impact of targeted drug administration on overall transmission in elimination settings.

**HOW THIS STUDY MIGHT AFFECT RESEARCH, PRACTICE OR POLICY**

- Findings will provide an evidence base to mobilise funding to improve coverage of appropriate interventions in these vulnerable groups and have practical implications for the timing and targets of indoor residual spraying retreatment campaigns.

## Introduction

 Progress in malaria control and elimination has stalled across Africa, with estimated numbers of cases plateauing around 230 million between 2020 and 2022.^1^ National Malaria Programmes need new tools and strategies to overcome remaining malaria control challenges. These challenges include gaps in routine intervention coverage, rising insecticide resistance (IR), outdoor mosquito biting observed in many countries^2^,^4^ and high human and parasite mobility.^5^ New initiatives addressing intervention gaps with subnational tailoring strategies and innovations around vector control, including product formulation and spatial repellents, hold promise in addressing IR and outdoor biting. However, relatively little has been done to evaluate tailored delivery platforms to key populations or address parasite mobility linked to human movement in Africa.

Much has been learnt from tailoring interventions for key populations in malaria elimination settings in Southeast Asia, where people who work or live in or near the forest are at highest risk of being infected with malaria.^6^ In these settings, tailored interventions have shown impact on malaria prevalence in forest-goers by combining vector control with case management or chemoprevention^7^,^9^ and leveraging novel community delivery pathways.^10 11^ In Africa, key populations at higher risk of infection have been identified across low transmission settings and include boarding students, nomadic pastoralists and gold miners in Senegal^12^; migrant sugar plantation workers in Eswatini^13^; migrant farm workers in the Ethiopia highlands^14^,^16^ and in Namibia, seasonal agricultural workers engaged in herding and farming, students and individuals travelling to Angola for harvesting mopane worms and fruit.^17^,^19^ Evidence to support tailored and targeted intervention strategies to improve coverage and reduce malaria within these groups and impact onward transmission to surrounding communities is lacking.

In low-endemic contexts such as Namibia, no malaria intervention strategy has specifically targeted these populations. Seasonal farm workers and cattle herders are increasingly prioritised by the Ministry of Health and Social Services (MoHSS) due to concerns around cross-border mobility, population mixing at worksites and anticipated intervention gaps. Workers require a targeted delivery approach to achieve high coverage and an intervention package able to clear existing infections and address both indoor and outdoor biting. In this study, a quasi-experimental design was used to evaluate the impact of a tailored package of interventions delivered through agricultural worksites on coverage and malaria outcomes in agricultural workers and cattle herders in northern Namibia and their surrounding communities.

## Materials and methods

### Study area and target population

**Figure 1:**
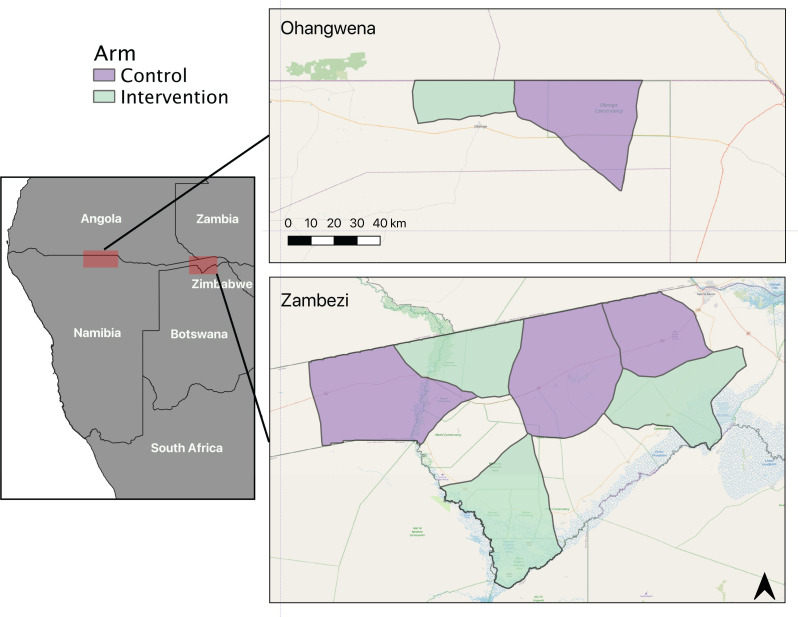
Control (purple) and intervention (green) health facility catchment areas in Ohangwena and Zambezi Regions in northern Namibia.

The study was conducted in 2019–2020 in a total of eight health facility catchment areas (HFCAs) (figure 1) in Zambezi and Ohangwena Regions of northern Namibia, which border Zambia and Angola, respectively. The total estimated population in the study area was 33 000 according to health catchment data in 2019. Malaria transmission in northern Namibia is almost entirely caused by *Plasmodium falciparum* and is highly seasonal, coinciding with agricultural periods and peaking from January to May. The annual incidence since 2010 has been <10.2 cases per 1000 individuals,^20 21^ but increased to 32.5 cases per 1000 individuals in 2016 following an outbreak.^22^ The main vector in Namibia is *Anopheles arabiensis* and entomological studies have demonstrated that the majority of infectious bites occur outdoors in both regions.^4^ The community-level prevalence of infection in Zambezi Region, measured by loop-mediated isothermal amplification, was 2.2% in 2016.^19^

Clinical case management in Namibia includes screening of febrile patients presenting to health facilities with a rapid diagnostic test (RDT) and first-line treatment with artemether-lumefantrine (AL). Trained health extension (ie, community health) workers may also support diagnosis and treatment of malaria within communities, either through (i) active case screening under supervision by non-governmental organisations, (ii) reactive case detection (RACD) with RDTs to households within a 500 m radius of an index case household or (iii) passive case detection in clinics supervised by health facility staff. All cases are reported through the health facility and in practice, active screening is rarely done due to funding gaps and RACD implemented inconsistently. During this study, activities outside of routine case management at health clinics ceased during the SARS-CoV-19 shelter-in-place between 28 March and 6 May 2020. Vector control remains the cornerstone of malaria control in Namibia, with annual preseason indoor residual spraying (IRS) campaigns and targeted LLIN distributions supplemented by larval source management.^3^ In 2019, Namibia implemented preseason blanket IRS of households with dichlorodiphenyltrichloroethane and deltamethrin or pirimiphos-methyl (Actellic 300CS) (for modern structures).

The target population included agricultural workers engaged in farming and cattle herding in Zambezi Region and cross-border cattle herders in Ohangwena Region. The population size is known to vary seasonally, but formative research approximated the maximum at 6000. Formative work and prior case-control studies have highlighted that interventions are typically directed at households, and coverage at worksites (farms and cattle kraals) may be limited by distance to roads and site closure during the spray season.^3 17 23^ In addition, sleeping structures at worksites are frequently classified as unsprayable, and outdoor exposure to biting mosquitoes in the evening and early morning hours is commonly reported.

### Study design

The study was a quasi-experimental trial with a control group and pre/post cross-sectional design. The implementation unit was the health facility catchment area; eight catchment areas were stratified by population size and annual incidence, and randomly assigned to study conditions. Study condition assignment was not blinded. The study was carried out between 20 November 2019 and 30 June 2020, and baseline and endline cross-sectional surveys were conducted to inform the primary outcomes of intervention coverage and malaria prevalence. Weekly incidence data collection from routine surveillance and entomological assessments informed secondary outcomes of community malaria incidence and residual efficacy of the insecticide. The protocol was registered with ClinicalTrials.gov (NCT04094727) and is available in the online supplemental appendix S4.

### Intervention

The design and delivery of the intervention package was designed to clear existing malaria infections and address both indoor and outdoor biting. Interventions were delivered by health extension workers (HEWs) and study staff to all eligible participants at worksites in intervention areas between late January and early March 2020, in coordination with networks of employers.

The intervention package included mop-up IRS with an organophosphate compound (Actellic 300CS, Syngenta), targeted drug administration (TDA) with AL (Komefan 140, Mylan Laboratories) and distribution of a backpack with a long-lasting insecticide net (LLIN) (Olyset Plus, Sumitomo) and topical repellent (Autan, SC Johnson) (Zambezi Region only). Two rounds of TDA were planned, 1 month apart, and administered at each farm site to everyone present who met eligibility criteria; the second round of TDA and LLIN mop-up was cut short due to the SARS-CoV-19 lockdown in April 2020.

IRS was carried out in all sprayable structures at worksites within intervention areas not covered during the routine campaign, as well as tents and tarps currently in use. Individual inclusion criteria to receive topical repellent, TDA and LLINs in Zambezi Region included (i) employment as an agricultural worker or cattle herder within an intervention area and (ii) sleeping or working outside at least one night within the past 30 days. In Ohangwena Region, eligibility criteria for distribution of targeted TDA and LLINs included (i) working as cattle herder in the past 30 days for an employer within the intervention area and (ii) travel to Angola during the prior 60 days (for TDA) and/or planned travel during the malaria transmission season (for LLINs). There were no additional inclusion criteria for TDA, but exclusion criteria included: treatment with an antimalarial drug in the past 4 weeks, weight under 5 kg, severe malaria, contraindicated treatments or pregnancy in the first trimester (assessed by testing if no menstruation was reported in the prior month). Participants were instructed to report adverse events to a study HEW or nurse at the nearest healthcare facility.

### Outcomes

The primary outcome measures were measured at baseline and endline and included (i) self-reported coverage of any intervention among the targeted population and (ii) prevalence of *P. falciparum* among the target population by quantitative PCR (qPCR). Secondary outcomes reported in this manuscript are (i) routine confirmed incidence of malaria at health facilities measured throughout the study period, (ii) adherence to treatment based on a 28-day pill count and (iii) the residual efficacy of Actellic 300CS using WHO cone assays at 1 week and 4 months, in the context of morphological species complex identification. Other findings related to acceptability and cost-effectiveness will be reported elsewhere.

### Epidemiological component

Primary outcomes were assessed pre-intervention and post-intervention through cross-sectional surveys and the secondary outcome through routine case data.

*Eligibility*. Workers were eligible for inclusion in the cross-sectional surveys in Zambezi Region if they were employed at the selected worksite, identified their current primary occupation as cattle herder or agricultural worker and had slept or worked at least one night at the site in the past week. In Ohangwena Region, cattle herders were eligible if they reported overnight travel to Angola for cattle grazing during the malaria transmission season. Individuals who were aged under 12 years or otherwise unable to consent were excluded.

*Recruitment*. Sensitisation activities were carried out with health workers, HEWs, community leaders and employers in both regions prior to the initiation of surveys. Baseline mapping and enumeration of the target population was conducted in October/November 2019 and identified all cattle post and farm owners through community and veterinary networks. Each location was visited to collect information on geographic location and current and anticipated number of workers at each site during the study period. Geolocated sites in Zambezi Region and the list of confirmed cattle owners in Ohangwena with at least one employee anticipated to graze in Angola were used as a sampling frame. Worksites were randomly sampled for cross-sectional surveys, and all eligible individuals working at the site were recruited.

*Data collection*. The baseline cross-sectional survey was conducted from November 2019 to January 2020 in Ohangwena Region, and from December 2019 to January 2020 in Zambezi Region. The start of the endline cross-sectional survey was delayed over a month due to the SARS-CoV-19 shelter-in-place and was conducted over a 6-week period from mid-2020 to May 2020. Survey visits were scheduled in advance, and a team consisting of a community health worker, interviewer and driver provided information and carried out individual informed consent. A standardised questionnaire was administered by interview at both time points, on a password-secured tablet programmed in Open Data Kit (V.1.22.4). Data collected included information on sociodemographics, housing, behavioural risk factors, travel history and ownership/use of malaria interventions.

Each participant was tested for malaria by Carestart Malaria HRP2/pLDH RDTs (Access Bio, Somerset, New Jersey, USA) and four dried blood spots were collected on filter paper for subsequent molecular testing. Individuals testing positive for malaria by RDT were treated according to the Namibia MoHSS guidelines. Workers were eligible to receive the interventions regardless of their participation in the surveys. Pill counts were done 7–10 days after TDA with AL in a subsample of 320 participants. HEWs actively inquired about adverse events during the adherence follow-up visit.

The confirmed case incidence from all reporting health facilities was captured throughout the study on a weekly basis. Reported cases included those diagnosed at health facilities and by HEWs who may be attached to a given facility. Routine programme data on routine spray campaign coverage was collected for the 2019–2020 spray season at the health facility level to control for potential sources of variation.

### Sampling considerations

The target sample size of 930 participants per arm was calculated to detect a 20% difference in the proportion of target population who used any intervention at a worksite between control and intervention arms, assuming 50% coverage in control areas and a design effect of six, with 90% power at the 5% significance level and allowing for 20% non-response. Based on the estimated size of the target population in each region and the average size of worksites from enumeration, 60% of the target sample was from the Zambezi Region (156 farms/posts per arm), and the remaining 40% from the Ohangwena Region (124 per arm). Worksites were randomly sampled independently at each survey time point; however, there was a substantial degree of overlap between the two samples. Eligible participants within worksites were completely enumerated for inclusion.

### Entomological component

Entomological collections and residual efficacy assays were conducted in three study intervention sites in the Zambezi Region (online supplemental material). In brief, three tarp structures and a sentinel canvas tent were selected per site for insecticide residual efficacy testing, with all structures sprayed with Actellic 300CS 1 week prior to collections. Human landing catches (HLCs) were conducted in the sprayed structures over four nights, for 12 hours during each night and larvae collected. HLC-collected *Anopheles* were used for residual efficacy testing via WHO cone bioassays in the field at the first time point, with mosquito knockdown recorded at 30 and 90 min and mortality at 24 hours postexposure.^24^ All collected *Anopheles* larvae were reared to the adult stage and tested for susceptibility to pirimiphos-methyl (0.25) using the WHO tube test. On completion of residual efficacy and IR assays, all *Anopheles* specimens were morphologically sorted to species group using the Gillies and Coetzee identification key.^25^

Monthly IR assays and WHO cone bioassays were planned with wild-caught mosquitos on tarps and canvas, but operations were disrupted by the pandemic. Sentinel canvas tents were relocated to the courtyard of the Oshakati Insectary in Oshana Region, Namibia. At 4 months post-Actellic 300CS spraying, 12 WHO cone bioassays were conducted on the three sentinel canvas tents using Oshakati’s pyrethroid-susceptible *A. arabiensis* (KGB strain).

### Laboratory methods

Dried blood spots from cross-sectional surveys were stored at −20°C. DNA was extracted by Tween/Chelex method as described by Schwartz *et al*.^26^ To estimate *P. falciparum* parasite density, an ultrasensitive quantitative qPCR varATS assay was conducted.^27^ Reactions were run with 1X Taqman Gene Expression, 0.8 µM forward (cccatacacaaccaaytgga) and reverse (ttcgcacatatctctatgtctatct) primers, 0.4 µM varATS probe (6-FAM-trttccataaatggt-NFQ-MGB) and 5 µL of extracted DNA, for a total reaction volume of 25 µL. The qPCR reactions were run using the following settings: pre-incubation: 2 min at 50°C; initial denaturation: 10 min at 95°C; amplification and denaturation: 15 s at 95°C, annealing and elongation: 1 min at 55°C. Each reaction was run in the QuantStudio 3 (ThermoFisher A28567) for 60 reaction cycles and read fluorescence at the end of each cycle. Standards at the following parasite concentrations were included in each plate in duplicates: 10 000 p/μL; 1000 p/μL; 100 p/μL; 10 p/μL; 1 p/μL; 0.1 p/μL; 0.05 p/μL. Parasite densities were estimated using linear regression on standards using the QuantStudio Design and Analysis Software. All samples with detectable parasite DNA were counted as positive.

### Data management

All data management was carried out in R (V.4.2.3). For every surveyed site, altitude, day and night land surface temperature (LST), enhanced vegetation index (EVI) and precipitation were extracted and averaged over the 30 days preceding the survey date (online supplemental table S1).^28^,^32^ Similarly, the mean, min and max over the 30 days prior to the incidence data collection week of the same environmental covariates were extracted and aggregated over each health facility catchment area.

Cross-border migrants were defined as individuals who had moved from another country and established their main place of residence in Namibia within the past year. Initial descriptive analyses examined covariate balance across time points and regions and between control and intervention arms.

### Statistical analyses

Statistical analyses for the main outcomes were carried out in R (V.4.2.3) and the secondary incidence outcome in Stata/MP V.14.2 (College Station, Texas, USA). An intention-to-treat approach was used, in which all individual participants within intervention areas were included in the analysis. Descriptive statistics were weighted to account for the sampling design, which was stratified by time point, region and health facility, with probability of cluster selection proportional to the projected population in the catchment area and adjusted for non-response rates. For the primary outcomes, a two-way difference-in-difference (DID) approach within a generalised linear model framework, with binomial distribution and log link, was used to estimate the DID estimator, reported as both the OR and the risk difference (RD) (ie, difference in average outcome in the treatment group before and after treatment minus the difference in average outcome in the control group before and after treatment).^33^ The ‘svyglm’ approach was used to account for complex sampling design, along with a fixed catchment effect and cluster robust SEs. Imbalances in potential confounding variables across arms and time points were addressed by evaluating the influence of individual (age, gender, occupation type, nationality, travel history) and site-level (rainfall and altitude) factors associated with the outcome. Factors that changed the DID coefficient by 10% or more, or improved the model fit by 10 points or more based on the Akaike information criterion (AIC) were retained in the adjusted model. Other covariates were excluded in the final model due to collinearity or lack of confounding (site ownership, baseline LLIN and IRS coverage, EVI, LST). For the secondary incidence outcome, weekly cases and environmental covariates were modelled in a DID framework using a negative-binomial model and including adjustment for a population offset, rainfall and maximum day LST as a non-linear covariate and clustered SEs. Weeks 8–11 were excluded from the analysis as interventions were underway during that period. Subanalyses were re-run by region a priori, based on known occupational and travel differences. Following Lechner, we assume that the true intervention effect can be estimated and the parallel trends assumption holds, conditional on exogenous control variables that may lead to differences in time trends.^27 34^ To explore heterogeneity of intervention impact, a triple difference term was estimated using a DID estimate (ie, interaction term) as above, but considering as the coverage/malaria outcome a difference between dichotomised groups: (a) sex: men versus women; (b) age: ≥ 50 years vs <50 years; (c) nationality: Namibian versus non-Namibian.

*Sensitivity analyses*. We used several approaches to explore the sensitivity of our findings to potential biases and model assumptions. First, a cohort of individuals was identified at both time points, based on a unique name identifier used for population size estimation in the broader study.^35^ In a second approach, to improve sample size restrictions, the above cohort was augmented with cohort matches of (i) baseline participants not interviewed at endline and (ii) endline participants not interviewed at baseline. Matches within the cohort were identified using a nearest neighbour approach and implemented with the R MatchIt package based on age, gender, seasonal work and travel history in the previous 2 months. Last, we explored the impact of potential violations in the parallel trends assumption using the R package ‘HonestDID’ for our secondary outcome of incidence after aggregating the weekly data across three periods: pre-intervention (weeks 1–7), intervention (8–11) and post-intervention (12–22).

## Results

### Intervention

**Figure 2:**
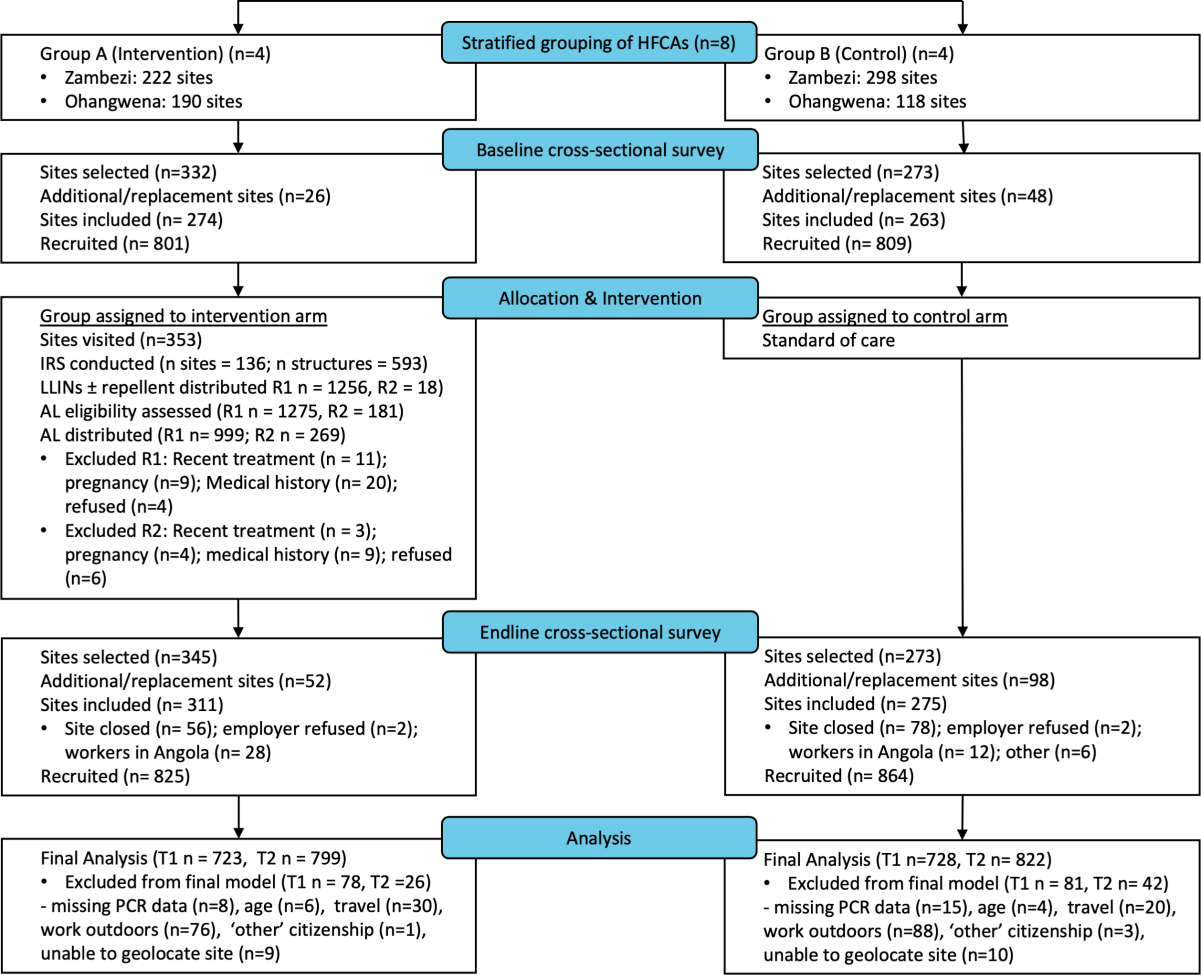
Participant flow chart, showing numbers (**n**) of participants at each time point (**T**) and interventions distributed in each round (**R**). HFCA, health facility catchment area; IRS, indoor residual spraying; LLIN, long-lasting insecticidal net.

The prestudy site mapping identified 828 worksites across the eight health facility catchment areas; a high proportion of intervention sites in both regions (82% and 92%) were accessed for at least one intervention round (figure 2). Overall, 1274 individuals received at least part of the intervention package, and targeted mop-up of IRS was conducted to fill gaps in 46% of sites in Zambezi Region and 18% of sites in Ohangwena Region (figure 2). AL was well tolerated in this study and only 10 individuals refused TDA. Overall, adherence to the 3-day AL treatment course was estimated to be 98.8% (253/256) based on the 28-day pill count.

### Population characteristics

The cross-sectional surveys captured a total of 537 sites and 1610 individuals at baseline and 586 sites with 1689 individuals at endline; 449 sites and 539 individuals were captured at both time points (figure 2 and table 1). A quarter (24%) of sites initially selected in Zambezi at endline could not be reached due to site closures, and many cattle herders from Ohangwena were in Angola at both time points, leaving 22% and 19% of worksites unreached. All sites in Ohangwena Region were cattle kraals or posts, and the majority of sites in Zambezi Region were farms with cattle posts in both control (68.9%) and intervention (70.8%) areas. The average number of workers employed at sites was low, with a median of two workers (IQR 1–2) in Ohangwena and three (IQR 2–6) in Zambezi Region, but a minority of sites employed as many as 18 workers.

**Table 1:** Weighted baseline and endline participant characteristics at agricultural worksites in northern Namibia (2019–2020), by study arm

	Baseline (%)		P value	Endline (%)		P value	Overall p value
	Control	Intervention		Control	Intervention		
Individual characteristics							
Number	809	801		864	825		
Male gender	71.1 (68.2–73.9)	78.0 (74.9–80.8)	**<0.001**	73.6 (69.5–77.5)	82.4 (78.8–85.6)	**<0.001**	**<0.001**
Mean age (range)	34.1 (12–96)	34.2 (13–94)	0.88	33.8 (13–96)	34.9 (12–97)	0.21	0.22
Occupation: cattle herder	36.2 (31.5–41.1)	49.0 (43.7–54.3)	**<0.001**	47.9 (42.5–53.4)	60.9 (54.9–66.6)	**0.001**	**<0.001**
Agricultural worker	63.8 (58.9–68.5)	51.0 (45.7–56.3)		52.1 (46.6–57.5)	39.1 (33.4–45.1)		
Seasonal employment	27.9 (23.7–32.4)	28.5 (24.4–32.9)	0.34	25.0 (21.7–28.6)	15.9 (12.7–19.5)	**<0.001**	**<0.001**
Site owner	9.0 (7.5–10.8)	5.6 (4.3–7.1)	**0.001**	7.0 (5.5–8.8)	4.7 (3.4–6.3)	**0.03**	**<0.001**
Nationality			0.08			0.31	**0.04**
Namibian	38.3 (33.9–43.0)	46.5 (41.4–51.8)		40.3 (35.0–45.8)	46.0 (40.8–51.4)		
Zambian	52.7 (47.8–57.6)	46.2 (40.8–51.7)		51.5 (45.4–57.5)	45.4 (39.8–51.1)		
Angolan	8.5 (6.5–10.9)	7.0 (5.2–9.1)		8.2 (5.9–11.1)	8.6 (6.1–11.6)		
Other	0.4 (0.0–1.1)	0.3 (0.0–0.9)		0.0 (-)	0.0 (-)		
Cross-border movement†	27.5 (23.6–31.7)	37.6 (32.8–42.6)	**<0.001**	15.6 (11.9–19.8)	22.2 (18.3–26.6)	**0.018**	**<0.001**
Cross-border migrationin then past year	15.6 (12.9–18.7)	10.8 (8.7–13.3)	**0.009**	12.1 (9.3–15.4)	10.0 (7.9–12.4)	0.27	**0.008**
Slept outside at worksite‡	28.3 (24.5–32.4)	44.5 (40.9–48.2)	**<0.001**	16.2 (13.3–19.4)	31.4 (28.3–34.6)	**<0.001**	**<0.001**
Worked at night‡	81.2 (77.9–84.3)	80.4 (76.5–84.0)	0.74	60.4 (54.6–66.1)	49.5 (44.7–54.3)	**0.004**	**0.008**
Malaria prevalence and self-reported intervention coverage							
PCR positivity§	3.0 (2.0–4.2)	4.0 (2.8–5.5)	0.21	8.8 (6.5–11.6)	3.1 (2.0–4.6)	**<0.001**	**0.006**
Intervention (any)	39.3 (34.8–43.9)	29.6 (25.6–33.9)	**0.002**	30.2 (25.7–35.0)	78.9 (74.9–82.5)	**<0.001**	**<0.001**
Slept under LLIN previous night	14.2 (11.8–16.9)	14.6 (11.9–17.6)	0.86	10.2 (8.0–12.7)	67.3 (63.1–71.4)	**<0.001**	**<0.001**
IRS at worksite	27.9 (23.4–32.8)	16.9 (13.1–21.4)	**<0.001**	23.9 (19.7–28.6)	38.0 (32.7–43.5)	**<0.001**	0.63
TDA	0.0 (-)	0.0 (-)	–	0.0 (-)	41.8 (38.0–45.7)	**<0.001**	**<0.001**
Topical repellent	0.0 (-)	0.0 (-)	–	0.1 (0.0–0.6)	39.7 (35.0–44.5)	**<0.001**	**<0.001**
Site characteristics	Mean (SE)			Mean (SE)			
Number	263	274		275	311		
Minutes to health facility	40.1 (1.74)	46.9 (3.79)	0.1	45.3 (2.54)	57.2 (5.03)	**0.04**	**0.006**
Rainfall (mm)	3.3 (0.009)	3.5 (0.017)	**<0.001**	2.8 (0.013)	2.9 (0.012)	**0.01**	**<0.001**
EVI	0.36 (0.006)	0.33 (0.006)	**<0.001**	0.30 (0.002)	0.30 (0.002)	0.91	**<0.001**
Elevation (m)	1021 (4.53)	1054 (5.69)	**<0.001**	1028 (5.56)	1055 (5.77)	**0.001**	**<0.001**
LST (°C)	37.2 (0.26)	38.3 (0.24)	**0.003**	29.3 (0.09)	29.2 (0.11)	0.61	0.61

The χ2 tests were used for categorical variables and t-tests for continuous variables.

Values < 0.05 are shown in bold.

* 10 missing.

† Defined in Ohangwena as grazing cattle in Angola within the last 2 months and in Zambezi as cross-border travel within the last 2 months (12 missing in Zambezi).

‡ 167 missing.

§ 23 missing.

EVIenhanced vegetation indexIRSindoor residual sprayLLINlong-lasting insecticide netLSTland surface temperatureTDAtargeted drug administration

Baseline and endline worker and site characteristics are described in table 1, stratified by study arm. Differences in the population composition and baseline coverage between arms and timepoints, in part, reflect a high degree of turnover in this population through the peak agricultural season. Regional differences in terms of gender, occupation, nationality and travel history as well as baseline intervention characteristics are reflected in online supplemental table S2 (online supplemental material). Although similarly aged, the majority of workers in the Zambezi Region were male (67%) and Zambian (64%), while cattle herders in Ohangwena Region consisted almost entirely of men (99%) from Namibia (82%) and Angola (18%). Outside work at night and cross-border travel was common in this population, particularly at baseline (table 1).

### Baseline intervention coverage and malaria prevalence

Self-reported baseline coverage of IRS at worksites was higher in control areas compared with intervention areas (27.9% vs 16.9%, p≤0.001) and differed between regions (table 1 and online supplemental table S1). This trend is mirrored in programme data from Zambezi, which reported a trend towards higher IRS coverage in communities within control HFCAs compared with intervention HFCAs (75% vs 72%). No TDA or use of repellent was reported in either arm at baseline, and self-reported use of nets was low.

Malaria prevalence by PCR was similarly low across control and intervention arms at baseline (3.0% vs 4.0%, p=0.21), with the majority of infections <100 parasites/μL (87%), undetected by RDT (90%) and afebrile (80.5%).

### Estimated impact on malaria and self-reported coverage

Overall, the endline prevalence of malaria by PCR was lower in intervention areas compared with control areas (table 1). This was driven mainly by Zambezi Region (4.4% vs 11.1%, p≤0.0001) and there was no difference observed in Ohangwena Region (0.7% vs 0.5%, p=0.77). Similar to baseline, infections detected through PCR at endline were predominantly low density (78% <100 parasites/μL), undetected by RDT (73%) and afebrile (78%). The crude coverage of any intervention at endline was higher in intervention areas (77.5%) compared with control areas (34.0%; p≤0.0001) in Zambezi Region and Ohangwena Region (81.4% vs 16.3%, p≤0.0001). However, the observed coverage in the control area of Ohangwena was lower at endline compared with baseline (16.8%, 95% CI 11.4 to 23.6 vs 44.8%, 95% CI 37.0 to 52.9), suggesting differential capture of the eligible population at these two time points.

**Figure 3:**
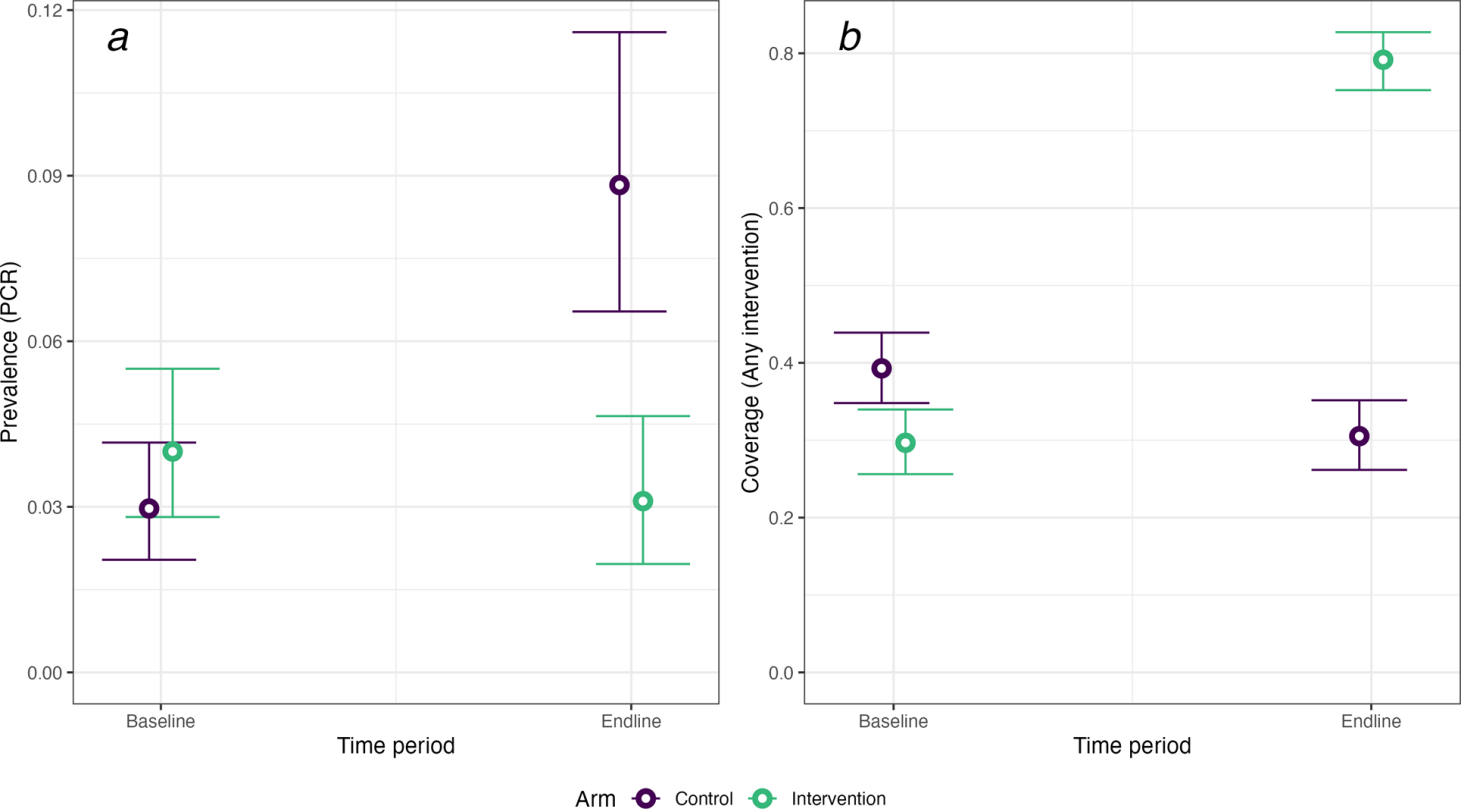
Baseline and endline estimates of (a) malaria prevalence by PCR and (b) coverage of any intervention in the target population, in control (purple) and intervention (green) arms.

**Figure 4:**
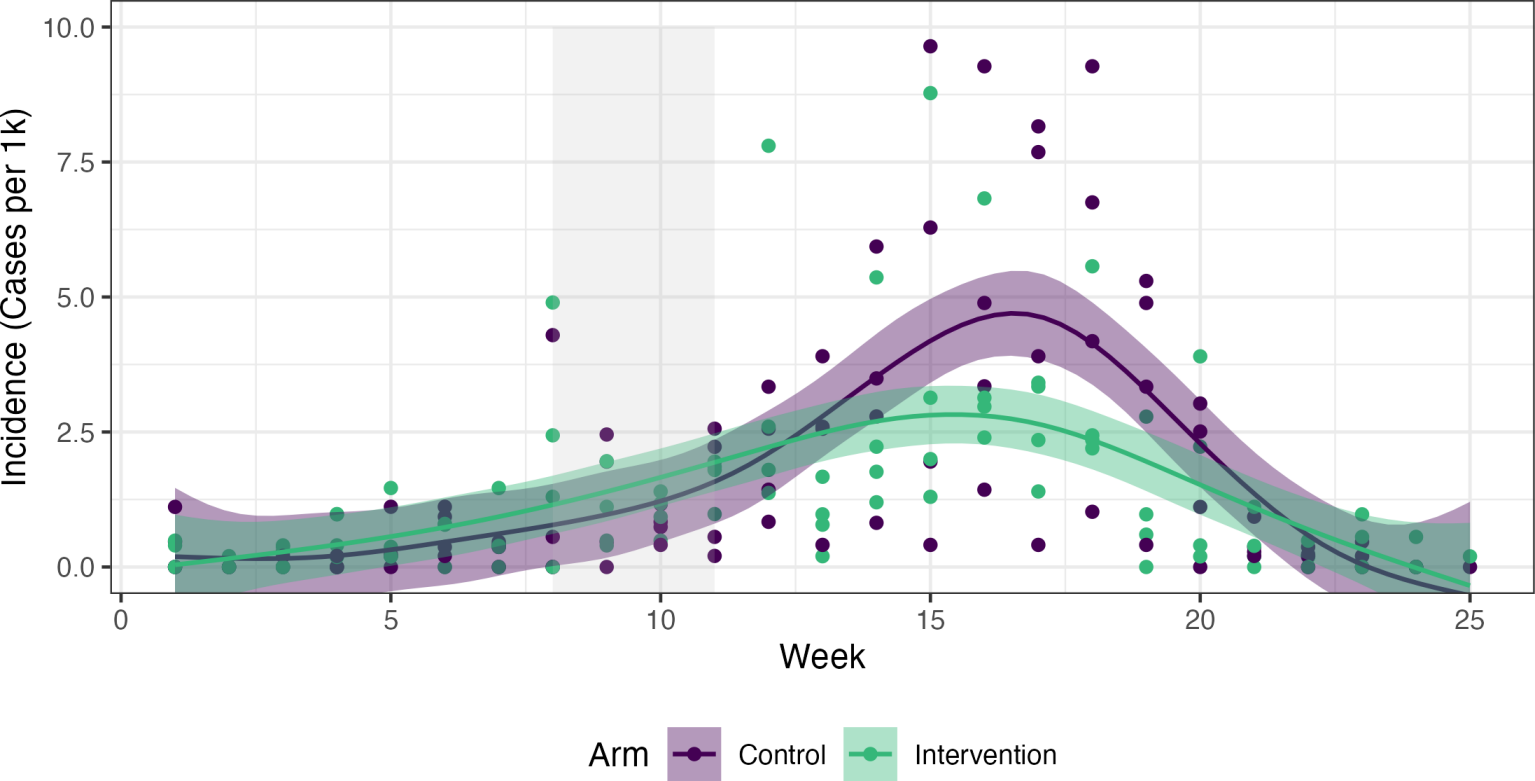
Smoothed weekly incidence (cases per 1000 population) from health facilities in control (purple) and intervention (green) arms over the study period, with the intervention distribution period shaded grey.

The unadjusted and adjusted DID models in table 2 present both RDs and ORs for malaria prevalence and coverage outcomes, and model coefficients are presented in the online supplemental table S3. Over the entire population, the intervention reduced malaria infection by 6.0% (95% CI −9.4 to –2.8), which corresponds to an OR of 0.24 (95% CI 0.1 to 0.5) (figure 3). Intervention coverage increased by 51.6% (95% CI 44.4 to 58.2). Stratified by region, the results were consistent with an impact on malaria prevalence in the target population in Zambezi (−7.7%, 95% CI −12.1 to –3.9) but not in Ohangwena Region (−0.9%, 95% CI −7.0 to 5.1), despite higher coverage. In the wider community, the intervention was not associated with a decrease in malaria incidence overall (−0.68 incident cases per 1000, 95% CI −2.0 to 0.6), but there was weak evidence of community impact in Zambezi Region (−1.29 cases per 1000, 95% CI −2.2 to –0.3) (table 3, figure 4).

**Table 2:** Unadjusted and adjusted DID model estimates for main study outcomes

	DID of malaria infection by PCR (95% CI)		DID of intervention coverage (95% CI)	
Study arm	Unadjusted	Adjusted*	Unadjusted	Adjusted†
Combined analysis				
OR	0.25 (0.1 to 0.5)	0.24 (0.1 to 0.5)	12.9 (8.6 to 19.3)	12.7 (8.5 to 19.0)
RD	−6.3% (−9.8 to −2.8)	−6.0% (−9.4 to −2.8)	53.3% (45.7 to 60.1)	51.6% (44.4 to 58.2)
Regional analyses				
Zambezi Region:OR	0.27 (0.1 to 0.6)	0.22 (0.1 to 0.5)	10.0 (6.1 to 16.3)	10.3 (6.3 to 16.7)
RD	−6.8% (−11.2 to −2.7)	−7.7% (−12.1 to −3.9)	49.3% (39.5 to 58.1)	49.3% (39.2 to 58.0)
Ohangwena Region:OR	1.0 (0.05 to 19.6)	0.61 (0.02 to 14.6)	37.6 (18.5 to 76.2)	37.2 (18.3 to 75.6)
RD	0.0% (−5.7 to 5.8)	−0.9% (−7.0 to 5.1)	68.0% (58.6 to 76.9)	67.7% (58.3 to 76.7)

* Adjusted for differences in individual age, gender, citizenship, rainfall, altitude, Sibbinda health facility and using robust SEs.

† Adjusted for differences in individual age, gender, Kasheshe health facility and using robust SEs

DIDdifference-in-difference estimatorRDrisk difference

**Table 3:** Unadjusted, weighted incidence at endline and adjusted DID model estimates for health facility incidence of malaria

	HFCA malaria incidence (cases per 1000 person-weeks)		
Study arm	Unadjusted incidence (95% CI)	Adjusted DID* (95% CI)	
		Rate ratio	Risk difference
Combined analysis			
Intervention	2.3 (2.1 to 2.5)	0.37 (0.2 to 0.9)	−0.68 (−2.0 to 0.6)
Control	1.6 (1.5 to 1.8)		
Regional analysis			
Zambezi Region: Control	3.2 (2.9 to 3.5)	0.27 (0.1 to 0.8)	−1.29 (−2.2 to –0.3)
Intervention	1.8 (1.6 to 2.1)		
Ohangwena Region: Control	0.5 (0.3 to 0.6)	1.17 (0.6 to 2.4)	0.35 (−0.1 to 0.8)
Intervention	1.1 (0.9 to 1.4)		

* Adjusted for region, health facility (1), weekly maximum land surface temperature and rainfall and using cluster SEs.

DIDdifference-in-difference estimatorHFCAhealth facility catchment area

### Estimated inequities in intervention

There was a trend towards greater impact on malaria prevalence in non-Namibians when compared with their Namibian counterparts, but up to a 23% absolute lower increase in intervention coverage (online supplemental table S4). The increase in intervention coverage was lower in younger participants, but there was no evidence of gender or age inequities in other models.

### Insecticide susceptibility and residual efficacy

Detailed findings on species identification and IR assays are described in the online supplemental appendix S2. In brief, mortality of 300 wild, female *Anopheles* mosquitoes caught from HLCs was 100% at 24 hours postexposure to pirimiphos-methyl 0.25%. The vast majority (99.3%) were identified as *Anopheles gambiae* s.l. with two specimens belonging to *Anopheles funestus* s.l. (one exposed to Actellic 300CS, one exposed to control) were identified.

Out of 71 WHO cone bioassays performed, 39 tests were deemed valid (ie, control mortality below 20%) and the average mortality per surface type was estimated as 95% for tarpaulin and 89% for canvas tents (p=0.885). At 4 months, WHO cone assays on canvas tents indicated the 24-hour postexposure mortality had declined to 44.6%.

### Sensitivity analyses

Overall, the cohort included more females and owners compared with the overall dataset, and tended to be more stable (fewer seasonal workers and less travel) (online supplemental table S5). There was a higher impact on coverage in the sensitivity analysis, which was 72.8% (95% CI 62.1 to 79.6) across the whole cohort (online supplemental table S6). There was no statistical evidence of a decrease in malaria prevalence in the cohort overall (RD −1.2% (95% CI −4.7 to 2.2)) or in either region; however, malaria prevalence in the cohort was notably low at all time points and across both arms (<4%). The augmented cohort approach yielded similar results with slightly stronger estimates of impact in the cohort augmented with endline matches (online supplemental table S6); in Zambezi Region, this cohort estimated a 5.7% decrease in prevalence (95% CI −9.9 to –2.5). The sensitivity analysis in the community incidence data suggested that the overall result is robust to violations of parallel trends up to the maximum violation in the pretreatment period (defined here as weeks 0–7 of the transmission season) and in the Zambezi Region, up to 1.5 times greater than the maximum violation in the pretreatment period.

## Discussion

This study demonstrates that malaria interventions targeted to agricultural worksites in Namibia have a substantial impact on malaria prevalence and effective coverage in farm workers and cattle herders over one transmission season. The intervention was associated with a 6.0% decline in the prevalence of malaria by PCR overall, corresponding to a substantial 75% reduction in odds, and a high (>75%) level of coverage in both regions for at least one of the interventions in the package. Malaria outcomes were driven by the greater impact observed in the Zambezi Region and a weaker effect observed on health facility reported incidence of malaria. Overall, sensitivity analyses did not support a strong effect on malaria prevalence in the cohort, except in Zambezi Region using the augmented endline cohort, which is likely due to population differences and a very low prevalence of malaria at both time points. Compliance to TDA was high in this study; however, residual efficacy of Actellic 300CS was found to be below the WHO threshold at 4 months. This demonstration project represents one of the first studies in Africa to target a malaria intervention package to worksites to improve outcomes in an underserved key population.

Populations at higher risk of malaria frequently experience lower coverage of routine interventions compared with the general population due to higher mobility, remoteness or other barriers to accessing treatment (such as nationality).^2336^,^38^ In addition to schoolchildren and miners, nomadic pastoralists and seasonal agricultural workers are increasingly recognised as core groups in Africa in which malaria exposures and intervention gaps intersect across different contexts.^39^,^41^ Tailoring the timing and delivery of standard interventions for these populations is a relatively simple way to improve effective coverage. In Namibia, for example, IRS occurs before the planting season when worksites are typically closed, meaning that sprayable structures at worksites are largely inaccessible to the spray team and tents and tarps are not included. Community engagement was crucial to achieve high coverage; this project engaged village leaders and existing resources in worksite mapping and closely coordinated intervention distribution with worksite employers.

High levels of subpatent infection combined with outdoor vector exposures and lack of sprayable structures indicate a need for new strategies and tools to address transmission in seasonal agricultural populations. Preventative treatment is increasingly targeted to and has shown a 15% absolute risk reduction on malaria prevalence in high-risk forest-going populations in Southeast Asia,^9^ while in Africa community-based mass drug administration (MDA) strategies are recommended for rapidly reducing malaria outcomes in lower transmission settings.^8 42 43^ More targeted applications directed to specific populations at higher risk of infection have not been widely implemented in Africa but could be a high-impact strategy that builds on established use cases.^7 42 44 45^ Alternative vector control tools to address outdoor biting are another area under active investigation.^46^ Tents and tarps are frequently used as shelters in emergency response settings, but there is little evidence on the effectiveness of spraying these surfaces. This study found that Actellic 300CS sprayed on tarp and canvas walls is effective in killing local, wild-caught *Anopheles* immediately postspraying but induced only a 44.6% mortality of *A. arabiensis* s.s. at 4 months; well below the 80% threshold set by WHO. Unfortunately, the residual efficacy against wild-caught *Anopheles* could not be assessed on a monthly basis, as was planned.

Despite large increases in coverage observed across both regions, only in Zambezi Region was the intervention package associated with a substantial (7.7%) decrease in the prevalence of malaria and a 78% reduction in odds of infection. This finding is consistent with the 72% reduction in parasite prevalence reported by Eisele *et al* in a community randomised trial of MDA in low transmission areas of Zambia.^43^ The effect of ITNs alone will vary with uptake but have halved malaria prevalence in some settings; the additional benefits of topical repellents are not well defined.^47 48^ A lower impact on coverage outcomes and higher impact on malaria was observed in non-Namibians. This conflicting finding may be due to the bulk of infections and coverage gaps co-occurring in non-Namibian populations and suggest a need for targeted efforts to improve access in these groups. There was no evidence of a difference in impact between cattle herders and farm workers within Zambezi Region, but we note that the study was underpowered for this analysis, and previous work indicates differences in risk levels and exposure profiles.^17^ There was weak evidence of spillover effects to the wider community in Zambezi Region, with an estimated average decrease of 1.3 cases per 1000 person-weeks. This impact is plausible given that population mixing and low intervention coverage in worksites could lead to ongoing transmission in the surrounding community.^49^

Several factors are likely to have limited the impact on prevalence in Ohangwena, including the lower prevalence of malaria and challenges in accessing the highest-risk subpopulations in this region. Formative research in Ohangwena identified substantial variation in movement patterns of cattle herders who graze in Angola, with some sub-groups such as bull herders staying almost year-round in Angola and other groups crossing the border more frequently or mostly remaining in Namibia.^36^ Although efforts were made to coordinate surveys with veterinary and cultural events that most cattle herders return for, highly mobile populations are likely to be under-represented in surveys and distribution efforts. Other potential reasons for differences in impact between regions could include the smaller sample size in Ohangwena and differences in baseline intervention coverage. A community-based net campaign targeting cattle herders was conducted in June 2019 following our formative findings, accounting for the higher use of nets at baseline in Ohangwena Region (35% vs 8%). Tents and tarps, although sprayed, were few and topical repellents were not distributed in Ohangwena due to limited availability.

Our study is subject to several limitations in design, implementation and interpretation. First, the study follow-up was short and conducted over one 4-month transmission season in Namibia. *The overall target sample size of 930 per arm was not achieved, which was due to the smaller population size and difficulties in accessing the target population in the Ohangwena Region*. Second, population turnover is high between the planting (baseline) and harvest (planned endline) periods, and numbers of local and cross-border workers are known to increase for the harvest. The second intervention distribution planned in March/April was halted due to the SARS-CoV-19 pandemic. Namibia did implement a shelter-in-place between 28 March and 6 May 2020, in which borders were officially closed. While it is possible that the pandemic may have reduced cross-border population mobility and favoured our intervention, migrant workers generally cross at informal border posts that are not controlled, and anecdotal evidence suggests that information in rural areas and subsequent impact on behaviour was limited. Disruptions to intervention distribution and delays in the endline survey meant that workers coming only for the harvest season may not have accessed the intervention and many workers may also not be captured at endline, given many sites were already closed. Overall, the direction of biases related to population mobility is likely to be towards the null. The sensitivity analysis suggested a higher uptake of the intervention and lower impact on malaria in the cohort of participants present at both time points. Limitations of this approach included a small sample size and low prevalence of malaria at both time points, which was in part overcome by augmenting the cohort with endline matches. Differing characteristics between the cohort and target populations raise questions around generalisability. Finally, it is not possible to assess the parallel trend assumption on which the DID approach relies, which states that the control arm would follow the same trend as the intervention arm, in the absence of treatment. However, the geographical proximity of health catchment areas makes large violations less likely and the incidence sensitivity analysis was found to be relatively robust to violations. Specimens used for the insecticide susceptibility and residual efficacy bioassays were sorted to morphological species complexes and instead identified species complexes (*A. gambiae* s.l., *A. funestus* s.l.) should be confirmed to species-level using molecular methods.^50 51^ However, the insecticide susceptibility test results align well with findings reported in.^3 3^

In conclusion, our study found that targeting tailored malaria interventions to high-risk agricultural workers through their worksites is an effective approach to improving coverage in this vulnerable population and likely to reduce transmission at worksites. In the higher transmission setting of the Zambezi Region, we saw an impact on malaria prevalence within the target population and a smaller impact on the incidence of malaria in surrounding communities. Targeted focal mass drug administration together with tailored vector control interventions are likely to help address the high proportion of subclinical infections, gaps in IRS and outdoor exposures.

## supplementary material

10.1136/bmjgh-2024-015565 online supplemental file 1

## Data Availability

Data are available in a public, open access repository.
