# Supplementary material for: Targeting malaria in high-risk populations in low endemic regions in northern Namibia: a quasi-experimental controlled trial to reduce malaria in seasonal agricultural workers and cattle herders
Source: BMJ Glob Health. 2025 Feb 17;10(2):e015565. doi: 10.1136/bmjgh-2024-015565 (PMC11836845; doi:10.1136/bmjgh-2024-015565)
Supplement: online supplemental file 1 [file bmjgh-10-2-s001.pdf]

Supplement to: **Targeting malaria in high-risk populations in low endemic regions in Northern Namibia: A quasi-experimental controlled trial to reduce malaria in seasonal agricultural workers and cattle herders**

Table of contents

Appendix S1 – Supplementary tables 2
Appendix S2 – Entomological supplement 7
Appendix S3 – Reflexivity statement 14
Appendix S4 – Study protocol 17

Appendix S1 – Supplementary tables

Table S1. Source and resolution of environmental covariates extracted for each data point.

Environmental covariate	Temporal resolution	Spatial resolution	Data Source [Reference]
Precipitation (millimeters)	Daily, aggregated weekly	0.05 degree (~5 km)	CHIRPS [1]
Land surface temperature (kelvin)	8 days	1km	MODIS [2]
Enhanced vegetation index	16 days	250m	MODIS [3]
Altitude (meters)	NA	90m	SRTM [4]
M meters			

Table S2. Baseline participant characteristics of control and intervention worksites in northern Namibia (2019-2020), by region and study arm

	**Zambezi Region N (%)**		**p-value**	**Ohangwena Region N (%)**		**p-value**
	Control	Intervention		Control	Intervention	
N sites [surveyed]	167	130		96	144	
Individual characteristics						
	n=615	n=561		n=194	n=240	
Male gender	65.1 (62.1-68.0)	69.8 (66.2-73.3)	**0.04**	99.0 (96.5-99.9)	99.6 (97.8-100)	0.44
Mean age [Range]	33.8 [12-96]	34.0 [13-94]	0.83	35.3 [12-80]	34.7 [13-92]	0.65
Occupation: Cattle herder	22.4 (18.8-26.3)	29.8 (25.1-34.9)	**0.01**	100.0 (-)	100.0 (-)	-
Agricultural worker	77.6 (73.6-81.2)	70.2 (65.1-74.9)		0.0 (-)	0.0 (-)	
Seasonal employment	29.2 (24.3-34.5)	35.3 (30.1-40.8)	0.14	21.6 (16.0-28.2)	10.4 (7.0-14.7)	**0.004**
Site owner	11.0 (9.1-13.1)	7.5 (5.8-9.6)	**0.01**	0.0 (-)	0.4 (0.0-2.2)	0.30
Nationality: Namibian	29.6 (25.2-34.3)	31.9 (26.5-37.8)	0.62	78.9 (72.2-84.6)	85.4 (80.3-89.6)	**0.08**
Zambian	64.1 (59.2-68.7)	63.6 (57.6-69.2)		0.0 (-)	0.0 (-)	
Angolan	5.8 (3.8-8.4)	4.1 (2.5-6.4)		21.1 (15.4-27.8)	14.6 (10.4-19.7)	
Other	0.5 (0.1-1.7)	0.4 (0.0-1.3)		0.0 (-)	0.0 (-)	
Cross-border movement[1]	13.8 (10.1-17.1)	15.3 (11.7-19.5)	0.53	91.8 (85.8-95.8)	97.9 (95.4-99.3)	**0.02**
Cross-border migration	18.4 (15.3-21.9)	14.1 (11.3-17.3)	**0.05**	2.6 (0.9-5.8)	2.1 (0.7-4.6)	0.72
Slept outside at worksite[2]	22.1 (18.5-26.2)	40.0 (35.9-44.0)	**<0.001**	58.3 (49.5-66.7)	56.4 (48.8-63.8)	0.73
Worked at night[2]	85.6 (81.3-89.3)	95.9 (93.6-97.6)	**<0.001**	50.4 (41.4-59.3)	41.4 (33.9-49.2)	0.06
Malaria prevalence and self-reported intervention coverage						
PCR positivity[3]	3.0 (2.0-4.4)	3.9 (2.5-5.6)	0.35	2.7 (0.8-6.3)	4.2 (2.1-7.3)	0.36
Intervention coverage (any)	38.1 (32.9-43.5)	28.9 (23.8-34.4)	**0.01**	44.8 (37.0-52.9)	31.7 (25.5-38.3)	**0.009**
Slept under LLIN previous night	8.5 (6.6-10.8)	8.2 (5.7-11.3)	0.83	40.7 (32.9-48.9)	31.7 (25.5-38.3)	**0.07**
IRS at worksite	33.0 (27.7-38.5)	23.0 (17.9-28.8)	**0.009**	4.6 (2.0-9.0)	0.8 (0.1-2.9)	**0.03**
TDA	0.0 (-)	0.0 (-)	-	0.0 (-)	0.0 (-)	-
Topical repellent	0.0 (-)	0.0 (-)	-	0.0 (-)	0.0 (-)	-
**Site characteristics**	Mean (sd)			Mean (sd)		
Minutes to HF [IQR]	40.1 (1.7)	46.9 (3.8)	0.10	-	-	
Rainfall (mm)	3.2 (0.004)	3.2 (0.006)	**<0.001**	3.6 (0.006)	3.8 (0.006)	0.17
EVI	0.4 (0.005)	0.4 (0.006)	0.06	0.2 (0.005)	0.3 (0.005)	**<0.001**
Elevation (meters)	969 (0.98)	958 (1.76)	**<0.001**	1162 (0.28)	1154 (0.43)	**<0.001**
LST (C)	35.0 (0.23)	36.4 (0.34)	**<0.001**	43.0 (0.24)	40.1 (0.28)	**<0.001**

PCR polymerase chain reaction; LLIN long lasting insecticide nets; IRS indoor residual spray; TDA targeted drug administration; HF health facility; IQR Inter-quartile range; EVI enhanced vegetation index; LST land surface temperature

[1] Defined in Ohangwena as grazing cattle in Angola within the last 2 months and in Zambezi, arriving from another country to work at the site within the last two months, [2] 167 missing, [3] 23 missing;

Chi-squared tests used for categorical variables and t-tests used for continuous variables

Table S3. Full coefficients of unadjusted and adjusted statistical models from the combined analysis in Table 2.

	**DID of malaria infection by PCR (95% CI)**		**DID of intervention coverage (95% CI)**	
	**Unadjusted**	**Adjusted[1]**	**Unadjusted**	**Adjusted[1]**
Intercept	-3.47 (-3.82, -3.12)	-5.40 (-6.75, -4.05)	-0.43 (-0.62, -0.24)	-0.94 (-1.27, -0.61)
Time	1.13 (0.67, 1.60)	1.52 (0.55, 2.48)	-0.38 (-0.66, -0.10)	-0.34 (-0.62, -0.07)
DID	-1.40 (-2.11, -0.68)	-1.39 (-2.13, -0.66)	2.55 (2.14, 2.96)	2.55 (2.14, 2.95)
Treated	0.29 (-0.20, 0.77)	-0.14 (-0.66, 0.38)	-0.43 (-0.71, -0.17)	-0.14 (-0.42, 0.14)
Female		-0.87 (-1.27, -0.47)		0.33 (0.15, 0.50)
Age category: <20		Ref		Ref
20-49		-0.18 (-0.69, 0.33)		0.10 (-0.17, 0.38)
50+		-0.49 (-1.21, 0.23)		0.36 (0.04, 0.68)
Citizenship: Namibian		Ref		-
Zambian		0.87 (0.21, 1.53)		-
Angolan		0.79 (0.30, 1.27)		-
Rainfall		0.94 (-0.66, 2.54)		-
Rainfall squared term		2.94 (1.28, 4.59)		-
Altitude < 1050 m		1.97 (0.88, 3.04)		-
Sibbinda health facility		-1.63 (-1.40, -0.52)		-
Kasheshe health facility		-		0.81 (0.52, 1.10)

PCR polymerase chain reaction; DID Difference in difference estimator; CI confidence interval.
[1] Adjusted for differences in individual age, gender, citizenship, rainfall, altitude, Sibbinda health facility and using robust standard errors; [2] Adjusted for differences in individual age, gender, Kasheshe health facility and using robust standard errors

Table S4. Heterogeneity in intervention effect on primary outcomes, by age, gender and citizenship

	Adjusted DID (95% CI)	
	Malaria Prevalence (%)	Intervention coverage (%)
Age: <50 vs >= 50 years	-1.5 (-7.4, 4.5)	-16.9 (-31.6, -1.9)
Gender: Female vs male	2.0 (-3.0, 7.1)	-1.7 (-15.1, 11.9)
Citizenship: Foreign vs Namibian	-4.9 (-9.8, -0.1)	-23.3 (-33.9, -12.3)

DID Difference in difference estimator expressed as a risk difference; CI confidence interval
Adjusted for variables in main models and using robust standard errors

Table S5. Individual characteristics of 504 participants matched in baseline and endline datasets

	**Zambezi Region N (%)**		**p-value**	**Ohangwena Region N (%)**		**p-value**
	Control	Intervention		Control	Intervention	
N sites [surveyed]	84	86		57	89	
Individual characteristics						
	n=145	n=172		n=70	n=111	
Male gender	55.6 (48.5-62.4)	65.3 (58.5-71.7)	**0.04**	100.0 (-)	100.0 (-)	-
Mean age [Range]	35.7 [13-96]	35.9 [17-84]	0.85	36.7 [18-80]	38.8 [15-74]	0.34
Occupation: Cattle herder	27.4 (19.9-36.1)	35.2 (27.9-43.1)	0.15	100.0 (-)	100.0 (-)	-
Agricultural worker	72.6 (63.9-80.1)	64.8 (56.9-72.1)		0.0 (-)	0.0 (-)	
Seasonal employment	14.4 (9.6-20.4)	28.3 (21.8-35.6)	**0.002**	24.3 (14.4-36.7)	7.2 (3.3-13.3)	**0.004**
Site owner	18.4 (13.2-24.7)	10.2 (6.8-14.4)	**0.01**	0.0 (-)	0.9 (0.0-4.7)	0.29
Nationality: Namibian	33.6 (26.0-41.9)	33.6 (26.1-41.7)	0.13	87.1 (75.7-95.5)	91.9 (85.7-96.0)	0.34
Zambian	61.0 (52.4-69.2)	64.8 (56.6-72.3)		0.0 (-)	0.0 (-)	
Angolan	5.4 (2.6-9.7)	1.7 (0.5-4.1)		12.9 (5.5-24.3)	8.1 (4.0-14.3)	
Other	0.5 (0.1-1.7)	0.4 (0.0-1.3)		0.0 (-)	0.0 (-)	
Cross-border movement[1]	5.7 (2.9-10.0)	7.9 (4.4-13.0)	0.40	91.8 (85.8-95.8)	97.9 (95.4-99.3)	0.35
Cross-border migration	14.5 (9.2-21.3)	8.3 (4.8-13.3)	**0.08**	2.6 (0.9-5.8)	2.1 (0.7-4.6)	0.83
Slept outside at worksite[2]	21.7 (15.6-29.0)	41.8 (35.0-48.8)	**<0.001**	58.3 (49.5-66.7)	56.4 (48.8-63.8)	0.96
Worked at night[2]	87.3 (80.0-92.6)	98.8 (96.0-99.8)	**<0.001**	50.4 (41.4-59.3)	41.4 (33.9-49.2)	0.56
Malaria prevalence and self-reported intervention coverage						
PCR positivity	1.4 (0.3-4.2)	2.4 (0.9-5.2)	0.45	1.4 (0.0-7.4)	2.7 (0.6-7.5)	0.52
Intervention coverage (any)	41.6 (32.5-51.2)	31.6 (23.7-40.3)	0.10	37.1 (25.3-50.2)	24.3 (16.7-33.4)	**0.08**
Slept under LLIN previous night	11.5 (7.4-16.8)	9.2 (4.6-15.9)	0.50	35.7 (24.0-48.8)	24.3 (16.6-33.4)	0.12
IRS at worksite	37.3 (28.7-46.6)	25.8 (18.4-34.4)	**0.05**	2.9 (0.3-10.0)	0.9 (0.0-4.8)	0.36
TDA	0.0 (-)	0.0 (-)	-	0.0 (-)	0.0 (-)	-
Topical repellent	0.0 (-)	0.0 (-)	-	0.0 (-)	0.0 (-)	-

PCR polymerase chain reaction; LLIN long lasting insecticide nets; IRS indoor residual spray; TDA targeted drug administration; HF health facility; IQR Inter-quartile range.
[1] Defined in Ohangwena as grazing cattle in Angola within the last 2 months and in Zambezi, arriving from another country to work at the site within the last two months, [2] 43 missing;
Chi-squared tests used for categorical variables and t-tests used for continuous variables

Table S6. Sensitivity analyses in 539 participants matched in baseline and endline datasets

**Analysis**	**DID Outcomes (95% CI)**			
	**Malaria prevalence**	**p-value**	**Coverage**	**p-value**
Main	-6.0% (-9.4, −2.8)	<0.001	51.6% (44.4, 58.2)	<0.001
Cohort	-1.2% (-4.7, 2.2)	0.48	72.8% (62.1, 79.6)	<0.001
Cohort augmented with baseline matches	-1.6% (-5.2, 2.0)	0.38	65.7% (48.5, 79.8)	<0.001
Cohort augmented with endline matches	-3.2% (-6.9, 0.3)	0.08	83.1% (74.2, 87.2)	<0.001

DID Difference in difference estimator expressed as a risk difference; CI confidence interval
Adjusted for variables in main models and using robust standard errors

1. Funk CC, Peterson PJ, Landsfeld MF, *et al.* A quasi-global precipitation time series for drought monitoring. Reston, VA: U.S. Geological Survey 2014. Accessed March 10, 2021. http://dx.doi.org/10.3133/ds832
2. Wan Z, Hook S, Hulley G. MODIS/Terra Land Surface Temperature/Emissivity 8-Day L3 Global 1km SIN Grid V061. NASA EOSDIS Land Processes DAAC. 2021. Accessed March 10, 2021. http://dx.doi.org/10.5067/MODIS/MOD11A2.061
3. Didan K. MOD13Q1 MODIS/Terra Vegetation Indices 16-Day L3 Global 250m SIN Grid V061. NASA EOSDIS Land Processes DAAC. 2021. Accessed March 10, 2021. http://dx.doi.org/10.5067/MODIS/MOD13Q1.061
4. NASA Shuttle Radar Topography Mission (SRTM). 2013. Accessed March 10, 2021. http://dx.doi.org/10.5069/G9445JDF.

Appendix S2 – Entomological supplement

Residual efficacy testing of Actellic on canvas tent walls

OBJECTIVES

Indoor residual spraying (IRS) decreases malaria transmission by killing *Anopheles* mosquitoes that are pyrethroid-susceptible and rest indoors on sprayed walls, or by reducing human-vector exposure indoors via its excitorepellent properties (Montoya et al., 2022). Therefore, the effectiveness or IRS is dependent on a range of elements, such as vector resting behavior (Msugupakulya et al 2020), vector susceptibility to the pyrethroid applied (Ranson et al 2011), IRS coverage (WHO 2015), quality of spraying (Fuseini et al 2020), and the residual efficacy of the IRS product over time (Mugenyi et al 2020).

Residual efficacy is typically measured as the number of months during which mosquito mortality 24h post-exposure to a sprayed wall remains above 80% (WHO 2006). A longer residual efficacy is important for increased IRS effectiveness (Mugenyi et al 2020). Towards understanding if Actellic 300 CS applied to tarp and canvas tents is effective for protecting target HRPs against *Anopheles* vectors, the Entomological Surveillance Planning Tool (Ávila et al., 2021; Mwema et al., 2022; Ávila et al., 2023; Lukubwe et al., 2023) (ESPT) was applied to formulate a priority entomological question regarding the residual efficacy of Actellic 300 CS: What is the efficacy of Actellic on tarp and canvas tent walls of sleeping structures on local *Anopheles* species at time of initial spraying, and on a monthly basis for 6 months? To address this question, the 24-hour post exposure to Actellic mortality of local, wild-caught *Anopheles* mosquitoes were measured using both the WHO cone bioassay and the WHO insecticide susceptibility assay.

METHODS

Sampling sites

The entomological collections and residual efficacy assays occurred in three study intervention sites: I1-I3 in the Zambezi Region (Table 1). A sampling site was represented by a single farm enclosure. Sites were selected for entomological sampling based on specific epidemiological, ecological, demographic, and logistical criteria: 1) epidemiological: sites with the highest known malaria burden were selected (Sangwali HFCA, and Sibbinda HFCA); 2) ecological: vegetation density, known mosquito larval sites, rainfall, were all considered, such that sites near known *Anopheles* larval sites were prioritized; 3) demographic: population density. Sites with higher population density were favored; and 4) logistical: accessibility by study teams, number of sleeping structures known to be occupied by target study population, variety and number of sleeping structure type of interest to this study (tarp, canvas tent). While roof type (zinc, thatch, tarp) was also considered, wall type was prioritized.

Table 1. Entomological sampling sites.

Site Code	GPS coordinates	HFCA
**I1**	-18.207884; 23.698509	Sangwali
**I2**	-18.213; 23.6585	Sangwali
**I3**	-18.1858; 23.5949	Sangwali

Structures included for residual efficacy testing of Actellic

Three tarp structures per site were selected for residual efficacy testing of Actellic. While canvas tents are also commonly used by the target population, due to a variety of constraints, none of the selected

sampling sites included a canvas tent. Thus, the study team set up three of the same types of canvas tents as those used by the target population (one canvas tent per site); these tents are referred to as the 'sentinel canvas tents'.

In the three intervention sites, structures had all been sprayed with Actellic through the IRS mop-up about 1 week prior to onset of collections in Intervention site 1 (I1) and about 3 weeks prior to onset of collections in Intervention site 3 (I3). The sentinel canvas tents were also sprayed with Actellic for the purpose of residual efficacy testing.

Human landing catches (HLCs)

HLCs were conducted in the Actellic-sprayed tarp structures. HLCs were conducted by trained community collectors for four nights, for 12 hours during each night (18h00 to 06h00). Collectors sampled mosquitoes for 50 minutes of each hour, allowing for 10 minute breaks at the top of each of the 12 collection hours. HLCs were conducted inside and outside sleeping structures where HRPs were known to live and sleep. Collectors wore long-sleeved shirts and rolled up trousers to knee-length, as well as no shoes, thus exposing tibiae, ankles, and feet. Collectors were instructed to collect all host-seeking mosquitoes having landed before attempting to blood feed on their lower limbs. Collectors switched collection location (inside or outside) at the end of each collection hour. Temperature and relative humidity (%RH) were recorded at the start of each collection hour. All collected mosquitoes were maintained alive and fed 10% glucose solution till the following morning, in preparation for residual efficacy testing via WHO cone bioassays.

WHO cone bioassays in the field (Feb-Mar 2020)

A total of 71 WHO cone bioassays (WHO, Guidelines for testing mosquito adulticides for indoor residual spraying and treatment of mosquito nets. Geneva: World Health Organization; 2006) were carried out in the tarp structures and in the canvas sentinel tents. Cone bioassays were conducted the following morning, when temperatures were cooler (around 26-27 degrees C), between 6 and 9am, after each HLC collection night, thus utilizing the wild-caught female *Anopheles* specimens. Only non-blood-fed, overnight sugar-fed, female *Anopheles* were used. Because the spray quality of the sprayed walls is unknown, cones were only affixed to the middle portions of the walls. Cones were not removed from substrate, unless otherwise required, in which case, placement of cones was marked. Four cones per structure, per testing day, were used. In each cone, between 10 and 13 female *Anopheles* were inserted. When mosquito numbers were too low, fewer cones were used, in order to ensure that at least 10 *Anopheles* specimens were inserted in each used cone (WHO 2006, WHO 2015). If mosquito numbers were too low, then the tarp structures were prioritized. A single piece of unsprayed, clean, flat, cardboard was used as the control substrate for all structures undergoing cone assays, where at least 10 female *Anopheles* in 1 cone were inserted. Mosquitoes were left in the cones for 30 minutes (WHO 2006, WHO 2015). Mosquito knockdown was recorded at 30 minutes and again at 90 minutes, after the mosquitoes had been removed from the cones. Mortality was recorded at 24h post-exposure. Cone bioassays were deemed valid if the corresponding control total mortality rate was below 20%. If the mortality rate was above 20%, the corresponding insecticide cone bioassay was excluded from the analysis (WHO 2006).

However, in March 2020, the Covid-19 pandemic halted the continuation of this fieldwork shortly after the first round of residual efficacy testing. Fieldwork was only permitted to resume during the dry season, when local mosquitoes densities are very low, and thus, adjustments to the research question had to be made. The sentinel canvas tents were retrieved from the field, and set up in the courtyard of the Oshakati Insectary, in Oshakati, Oshana Region, Namibia for residual efficacy testing against the susceptible *Anopheles arabiensis* s.s. (KGB strain) reared at the Oshakati Insectary, in Oshakati, Oshana Region, Namibia.

Anopheles *species identification*

Following of the WHO cone assays at the end of each of the four mornings, any remaining live mosquito was killed using chloroform fumes. All dead *Anopheles* were then placed in Eppendorf tubes with silica gel. Eppendorf tubes were labeled by the structure number and the cone number used for residual testing. All samples returned to the Katima Insectary for morphological identification to species group using the Gillies and Coetzee (1987) identification key.

Insecticide susceptibility assays

Following completion of the 4-day HLC collection period in each site, *Anopheles* larvae (instars 1-4) were collected in a variety of different larval sites surrounding each of the three sampling sites to 1) ensure tested mosquitoes were of the same species and population biting the target population in the sampling sites, and 2) ensure tested mosquitoes represent adequate genetic variability. Upon collection of larvae, samples were returned to the Katima Insectary housed at the Katima Mulilo UNAM campus for rearing and subsequent testing. Larvae were fed a mixture of dog biscuits and brewer's yeast, and adults were fed a 10% glucose solution.

Female *Anopheles* aged between 3-5 days old, were tested for susceptibility to pirimiphos-methyl [0.25] (the active ingredient in Actellic 300 CS) using the WHO tube test (WHO 2013). A total of two full insecticide susceptibility tests were conducted. test comprised four insecticide replicates, and two controls. Each replicate and control tube included 25 female *Anopheles*. Mortality at 24-hours post exposure was calculated. Each test was validated by verifying the control mortality at 24-hours post exposure.

WHO Cone Bioassays on the sentinel canvas tents in the Oshakati Insectary

In June 2020 (four months post-IRS mop-up with Actellic, and three months following the first round of WHO cone bioassays on the tarp structures and the sentinel canvas tents in the field), twelve WHO cone bioassays were conducted on the three sentinel canvas tents. Four WHO cone tests with 4-5 cones per test were conducted on each of the three sentinel canvas tents. Only non-blood fed, 3-5 day old females from Oshakati's pyrethroid-susceptible *An. arabiensis* were utilized.

RESULTS

IR testing and morphological species identification

A total of 300 wild, female *Anopheles* mosquitoes were tested for susceptibility to pirimiphos-methyl (Actellic) 0.25%. In Test 1, the control mortality was 0%, and in Test 2, the control mortality was 2%, rendering both tests valid. Mortality at 24-hours post-exposure for both Tests 1 and 2 were 100%.

Upon completion of IR tests, specimens were morphologically sorted to species group. In total, 199 females of *Anopheles gambiae* s.l. were exposed to Actellic, and 99 *An. gambiae* s.l were exposed to the control papers. Only two specimens belonging to *Anopheles funestus* s.l (one exposed to Actellic, one exposed to control) were identified.

WHO cone bioassays in the field

Out of 71 WHO cone bioassays performed, 39 tests were deemed valid (i.e., control mortality below 20%). Replicate mortality was corrected if control mortality was between 5 and 20%, using Abbott's formula: *(Observed mortality % - Control mortality %)/(100 - Control mortality %))*100.*

Replicate and control mortality were calculated for each cone on each surface, allowing for the subsequent calculation of average mortality per surface, per site (Table 5).

Table 5. Summary of 24-hour post exposure mortality of local *Anopheles* per surface type, per site.

Site number	Surface type	Average mortality (%) per surface *per site*
**I1**	Tarpaulin	100
**I2**	Tarpaulin	93
**I3**	Tarpaulin	100
**I1**	Canvas Tent	100
**I3**	Canvas Tent	83

Thus, the average mortality per surface type is 95% for tarpaulin, and 89% for canvas tents. The WHO considers the insecticide sprayed on a wall to be effective if it kills at least 80% of mosquitoes of a single species at 24-hour post-exposure. A comparison of 24-hour mortality per cone by surface analysis (Bonferroni) confers a p-value of 0.885, therefore indicating that there is no significant difference in 24-hour mortality between Tarpaulin and Canvas.

WHO cone bioassays at the Oshakati Insectary

The sentinel canvas tents were brought to the Oshakati Insectary at 4 months post spraying with Actellic. WHO cone assays were conducted on all three tents, using the pyrethroid-susceptible *Anopheles arabiensis* (KGB strain) colony reared at the Oshakati Insectary. Twelve tests were conducted, for a total of 58 replicates (19 replicates for Tent 1, 20 reps for Tent 2, and 19 replicates for Tent 3) exposing 558 mosquitoes (508 females; 50 males). Males were only used when there were insufficient numbers of females. All tests were validated, as control mortality at 24-hour post-exposure was below the 20% mortality threshold.

The 24-hour post-exposure mortality for the 58 replicates was 44.6%, which is well-below the WHO 80% threshold for efficacy. This suggests that the efficacy of Actellic on canvas tents on susceptible *An. arabiensis* attains the 80% threshold *before* 5-months post-spraying. Hence, residual efficacy of Actellic on canvas tents on susceptible *Anopheles arabiensis* should be investigated on a monthly basis immediately post-spraying in order to pinpoint exactly when mortality crosses the 80% threshold.

DISCUSSION, LIMITATIONS AND CONCLUSIONS

The WHO considers the insecticide sprayed on a wall to be effective if it kills at least 80% of mosquitoes of a single species at 24-hour post-exposure. Based on this study's residual efficacy results, it appears that while Actellic sprayed on tarp and canvas walls is effective in killing local, wild-caught *Anopheles* immediately post-spraying, its effectiveness on canvas walls quickly declines in the following months. By month 4 post-spraying, Actellic on canvas walls only induced a 44.6% mortality of *Anopheles arabiensis* s.s.. Given this low mortality rate at month 4, the residual efficacy against *An. arabiensis* s.s. of Actellic on canvas wall likely drops below the 80% threshold well before the 4-month mark, and this should be investigated. Unfortunately, the Covid-19 pandemic did not allow for the study team to monitor the residual efficacy for both tarp and canvas walls against wild-caught Anopheles on a monthly basis, as was planned.

Specimens used for the insecticide susceptibility and residual efficacy bioassays were sorted to morphological species complexes. However, the identified species complexes (*An. gambiae* s.l., *An funestus* s.l.) should be confirmed to species-level using molecular methods (Scott et al 1993, Koekemorer et al 2002) in order to confirm whether a predominant *Anopheles* species was tested during both bioassays. Thus, it cannot be confirmed that a meaningful number of at least one predominant *Anopheles* species was tested for susceptibility to Actellic, nor can this be confirmed for the residual efficacy bioassays. Consequently, it cannot be assured that the same species used for the insecticide susceptibility bioassays were also used for the residual efficacy bioassays. However, the insecticide susceptibility test

results align with the findings reported in Lukubwe et al 2023, in which the authors found that in Zambezi, all *An. gambiae* s.l. were susceptible to Actellic in both 2018 and 2019, and that 93.4% of the *An. gambiae* s.l. randomly selected for to species confirmation using PCR analysis were identified as *Anopheles arabiensis* s.s.. Therefore, it can be hypothesized that *An. arabiensis* s.s. was also the predominant species tested in this study.

Examining the residual efficacy of Actellic for the local *Anopheles* species is important for evaluating the effectiveness of IRS with Actellic, but further entomological investigations are required to consider the other key contributors to the efficacy of IRS. For instance, the indoor resting behavior (endophily) of local *Anopheles* in Zambezi was not evaluated in this study, and *An. arabiensis* s.s. is typically known as an exophilic, rather than endophilic, and exophagic species (Noor et al., 2013; Namibia MoHSS 2020; Kamwi 2005; Lukubwe et al., 2023). In 2019, Lukubwe et al (2023) found that all indoor resting specimens collected were identified as *An. arabiensis* s.s., and that Zambezi had the highest indoor resting rate (2 mosquitoes per structure) amongst the four regions investigated. Still, this indoor resting rate is quite low, and the authors argue that the small proportion of indoor resting *An. arbiensis* s.s. might be due to prior successful IRS campaigns selecting for resistant behaviors (Lukubwe et al 2023). Additional studies of indoor resting behaviors could measure the indoor resting rates throughout the night, on an hourly basis, in order to capture any indoor resting that might occur before sunrise (Avilà et al., 2021).

REFERENCES

Msugupakulya BJ, Kaindoa EW, Ngowo HS, Kihonda JM, Kahamba NF, Msaky DS, et al. Preferred resting surfaces of dominant malaria vectors inside different house types in rural south-eastern Tanzania. Malar J. 2020;19:22. doi: 10.1186/s12936-020-3108-0

Ranson H, N'Guessan R, Lines J, Moiroux N, Nkuni Z, Corbel V. Pyrethroid resistance in African anopheline mosquitoes: what are the implications for malaria control? *Trends Parasitol*. 2011;27(2):91–8. doi: 10.1016/j.pt.2010.08.004

WHO. *Indoor residual spraying: an operational manual for indoor residual spraying (IRS) for malaria transmission control and elimination - 2nd ed*. Geneva: World Health Organization, 2015

Fuseini G, Ismail HM, von Fricken ME, Weppelmann TA, Smith J, Ellis Logan RA, et al. Improving the performance of spray operators through monitoring and evaluation of insecticide concentrations of pirimiphos-methyl during indoor residual spraying for malaria control on Bioko Island. *Malar J*. 2020;19:35. doi: 10.1186/s12936-020-3118-y

Mugenyi L, Nankabirwa JI, Arinaitwe E, Rek J, Hens N, Kamya M, et al. Estimating the optimal interval between rounds of indoor residual spraying of insecticide using malaria incidence data from cohort studies. *PLOS ONE*. 2020;15(10):e0241033. doi:

WHO. *Guidelines for testing mosquito adulticides for indoor residual spraying and treatment of mosquito nets*. Geneva: World Health Organization; 2006

WHO. *Indoor residual spraying: an operational manual for indoor residual spraying (IRS) for malaria transmission control and elimination - 2nd ed*. Geneva: World Health Organization, 2015.

Ávila, M.I, Vajda, E.A., Gutiérrez, E.J., Gibson, D.A., Mosquera Renteria, M., Presley, N., O'Reilly, D., Burton, T.A., Tatarsky, A., Lobo, N.F. 2021. *Anopheles* drivers of persisting malaria transmission in Guna Yala, Panamá: an operational investigation. Malar J. 24:20(1):443.

Mwema, T., Lukubwe, O., Joseph, R., Malitia, D., Iitula, I., Katokele, S., Uusiku, P., Walusimbi, D., Barasa Ogoma, S., Tambo, M., Smith Gueye, C., Williams, Y.A., Vajda, E., Tatarsky, A., Lobo, N.F., Eiseb, S.F. 2022. Human and vector behaviors determine exposure to Anopheles in Namibia. *Parasit Vectors*. 15:463.

Ávila, M.I, Vajda, E.A., Gutiérrez, E.J., Gibson, D.A., Mosquera Renteria, M., Presley, N., O'Reilly, D., Tatarsky, A., Lobo, N.F. Entomological Surveillance Planning Tool (ESPT)-generated actionable evidence on human and vector behaviours optimizes present interventions and reduces exposure to Anopheles vectors in two communities of Guna Yala, Panamá. Malar J. 2023;22:26.

Lukubwe, O., Mwema, T., Joseph, R., Maliti, D., Iitula, I., Katokele, S., Walusimbi, D., Ogoma, S.B., Smith Gueye, C., Vajda, E., Tatarsky, A., Thomsen E., Tambo, M., Mumbengegwi, D., Lobo, N.F. 2023. Baseline characterization of entomological drivers of malaria transmission in Namibia: a targeted operational entomological surveillance strategy. Parasites & Vectors., 16: 220.

Gillies MT, Coetzee M. A supplement to the anophelinae of Africa South of the Sahara. Publ South Afr Inst Med Res. 1987;55:1–143.

WHO. Larval source management: a supplementary measure for malaria vector control, operational manual. 2013. https:// www. who. int/ publications/i/ item/ 97892 41505 604.

Scott JA, Brogdon WG, Collins FH. Identification of single specimens of the Anopheles gambiae complex by the polymerase chain reaction. Am J Trop Med Hyg. 1993;49:49520–9.

Koekemoer LL, Kamau L, Hunt RH, Coetzee M. A cocktail polymerase chain reaction assay to identify members of the Anopheles funestus (Diptera: Culicidae) group. Am J Trop Med Hyg. 2002;66:804–11.

Noor AM, Alegana VA, Kamwi RN, Hansford CF, Ntomwa B, Katokele S. Malaria control and the intensity of Plasmodium falciparum transmission in Namibia 1969–1992. PLoS One. 2013;8:63350.

Republic of Namibia Ministry of Health and Social Services (MoHSS). National Vector-borne Disease Control Programme 2020. Annual entomological surveillance report 2018–2020. Windhoek: Ministry of Health and Social Services

Kamwi RC. Malaria situation in Namibia a study of vector species and effectiveness of the past and current control strategies in selected parts of Namibia. Namibia: University of Namibia; 2005.

Appendix S3 – Reflexivity statement

1. How does this study address local research and policy priorities?

The research question addressed by this study was directly informed by local operational research priorities set out by the Namibian Ministry of Health and Social Services (MoHSS) National Vector-borne Disease Control Programme (NVDCP) in collaboration with local research institutions. The NVDCP was involved in the conceptualization and design of the formative research and follow on trial described here, as well as supporting their successful implementation.

2. How were local researchers involved in study design?

Local researchers were involved in the study at multiple levels. The study was co-led with the University of Namibia (UNAM) and involved significant oversight of activities (DM) and implementation of entomological study components together with Witts and UCSF (TM and AMB). There was also a LMIC researcher employed as research staff through UC Global Programmes (HN) who played a critical role in design and implementation of the study. Active involvement from MoHSS staff in the entomological work (ML) and community sensitization and study design (PU and JKH). In addition, there were high-income country researchers with extensive experience of conducting, leading, or organising research collaborations in Namibia (JLS, CSG, JOJ, RG, AB) and entomological expertise (EV). High income country researchers are slightly over-represented on the list of authors of this article, due to the structure of the funding supporting various elements of this work.

3. How has funding been used to support the local research team?

This project supported a full-time post-doctoral early career researcher based in Namibia to oversee implementation and coordination of the research and support interim analysis. It also supported effort of partners at UNAM, including leadership for the project, capacity building through student engagement on entomological research, and use of vehicles for research. Local field teams were recruited from the study areas through UC Global Programs.

4. How are research staff who conducted data collection acknowledged?

All research staff engaged in data collection or data generation, including students, were included as authors. Field teams were thanked for their contributions in the acknowledgements.

5. Do all members of the research partnership have access to study data?

All members of the partnership have access to data.

6. How was data used to develop analytical skills within the partnership?

Interim analysis for reports was led by in-country teams and entomological data analysed by students at UNAM, with technical support from more senior scientists within the partnership.

7. How have research partners collaborated in interpreting study data?

Reports of preliminary findings were discussed with the NVDCP at meetings led by in-country partners. Research partners have supported interpretation of the study findings and opportunities to input into the manuscript.

8. How were research partners supported to develop writing skills?

The research team writing the manuscript is predominantly composed of senior academics. The pre- and post-doctoral early career researchers (HN, EV, AMB) on the authorship team wrote specific sections of the manuscript with guidance and inputs from senior academics to refine their writing skills.

9. How will research products be shared to address local needs?

This research will be published as open access. Preliminary research outputs were shared at the conclusion of the study through in-country dissemination meetings with local stakeholders. We have also been able to share results at international conferences and seminars to engage with research and programmatic stakeholders in high-income and low- and middle-income countries.

10. How is the leadership, contribution and ownership of this work by LMIC researchers recognised within the authorship?

The leadership and contribution of work in this manuscript is represented by joint senior and second authorship positions from key LMIC research partners (DM and HN). In addition, the valuable support and contributions of the MoHSS into the conceptualization and implementation of this study is recognized by their authorship (PU, ML, JP) and acknowledgments. We acknowledge, however, that the authorship team is slightly skewed towards high-income countries, comprising nine of sixteen authors. The reason for this is that the study conceptualisation, oversight and funding was driven mainly from that side.

11. **How have early career researchers across the partnership been included within the authorship team?**

We have included early career researchers (HN, FR, AMB, TM, KT & EV) within the authorship team. They were either involved in study implementation, generation of entomological or molecular data or analysis components. Three of the six are based in LMIC countries.

12. How has gender balance been addressed within the authorship?

Eight authors are female (JLS, AMB, TM, KT, PU, JKH, CSG, and EV) and eight authors male (HN, FR, ML, JOJ, BG, RG, AB and DM).

13. How has the project contributed to training of LMIC researchers?

The project contributed most clearly to the training of LMIC researchers through the entomological component, which included structured implementation of UCSF's Entomological Surveillance Planning Tool (ESPT) and technical support and training around these activities. Data management was also led in country by LMIC partners with technical support from UCSF around form development and data cleaning. The postdoctoral LMIC researcher gained on-site leadership and management skills with support from more senior partners.

14. How has the project contributed to improvements in local infrastructure?

The project has raised considerable interest among the programme and other stakeholders in Namibia, resulting in allocated funds for malaria nets and IRS at worksites when resources allow. There was also support through this project towards infrastructure and sustainability of the Oshakati Insectary.

15. What safeguarding procedures were used to protect local study participants and researchers?

Safeguarding procedures used to protect local study participants and researchers included administration of informed consent and assent for minors aged over 12 years. This process was conducted privately, away from worksite owners, and emphasized that participation in the study would not influence their employment or treatment they might receive. All data collection was carried out in password-encrypted tablets and all electronic data and paper documents for the study securely stored.

Protocol

Targeting malaria high-risk populations with tailored intervention packages: A study to assess feasibility and effectiveness in northern Namibia

Republic of Namibia
Ministry of Health and Social Services

UNAM
UNIVERSITY OF NAMIBIA

UCSF
University of California
San Francisco

Version Number: 1
July 3, 2019

Table of Contents

1. Abbreviations and acronyms 5
2. Protocol summary 7
3. Study Personnel and Institutions 13
4. Background 16
 4.1 Malaria in high-risk populations 16
 4.2 HRPs in Namibia 16
 4.3 Malaria interventions for HRPs in Namibia 17
 4.4 Targeted delivery of malaria interventions to HRPs 18
5. Rationale 19
 5.1 Significance 19
6. Study aims 20
 6.1 Primary aim 20
 6.2 Secondary aim 20
 6.3 Primary research question and hypotheses 20
7. Study sites 21
8. Study Design 23
 8.1 Study overview 23
 8.2 Study population 24
 8.3 Outcome measures 25
 8.4 Matching and randomization 26
 8.5 Sample size 26
 Coverage of malaria interventions 26
9. Study Interventions 27
 9.1 Overview 27
  9.1.1 Community sensitization and understanding 27
  9.1.2 Targeted reactive case detection (RACD) 28
  9.1.3 Presumptive treatment 28
  9.1.4 Targeted Indoor Residual Spraying (IRS) 30
  9.1.5 LLIN distribution and/or vector control packs (sprayed tents/tarps and topical repellent) 31
  9.1.6 AL adherence and safety monitoring 32
10. Study evaluation methods 33
 10.1 Overview of evaluation activities by study aim, research objectives, outcomes, and data collection activities 33

10.2 Impact evaluation of targeted delivery of malaria interventions (presumptive treatment and enhanced vector control) ... 35
10.2.1 Outcome measures ... 35
10.2.2 Sample size ... 36
10.2.3 Data collection ... 36
Cross-sectional surveys ... 36
Case and control survey data ... 39
Confirmed malaria case incidence ... 41
10.2.4 Data management and analysis ... 41
10.3 Assessment of feasibility, acceptability of and adherence to interventions ... 42
10.3.1 Outcome measures ... 43
10.3.2 Sample size ... 44
10.3.3 Data collection ... 44
Intervention implementation data ... 44
10.3.4 Data management and analysis ... 48
10.4 Surveillance capacity building and assessment of targeted reactive case detection ... 49
10.4.1 Outcome measure ... 49
10.4.2 Sample size ... 49
10.4.3 Data collection ... 49
10.4.4 Data management and analysis ... 50
10.5 Population size estimation and turnover assessment ... 50
10.5.1 Outcome measure ... 50
10.5.2 Data collection ... 51
Multiplier method activities ... 51
Capture recapture activities ... 51
Data sources ... 52
10.5.3 Data management and analysis ... 53
10.6 Vector assessment in agricultural areas of Zambezi Region ... 54
10.6.1 Outcome measures ... 54
10.6.2 Data collection ... 55
10.6.3 Data management and analysis ... 57
10.7 GPS logger movement study ... 58
10.7.1 Outcome measure ... 58
10.7.2 Data collection ... 59
10.7.3 Data management and analysis ... 60

11. Laboratory analysis 60
12. Ethical considerations 62
12.1 IRB 62
12.2 Racial and ethnic origin 62
12.3 Inclusion of vulnerable subjects – children and pregnant women 62
12.4 Informed consent procedures 62
12.5 Alternatives to participation 63
12.6 Potential benefits of the proposed research 63
12.7 Potential risks of the proposed research 63
12.8 Adequacy of protection against risks 64
12.9 Management of sick participants 65
12.10 Data and safety monitoring plan 65
13. Potential limitations, data quality assurance, and dissemination plan 66
13.1 Potential limitations 66
13.2 Data quality assurance plan 67
13.3 Dissemination Plan 67
14. Timeline 69
15. References 70
16. Appendices 72

1 Abbreviations and acronyms

ACD	Active case detection
AL	Artemether-lumefantrine
BCC	Behavior change communication
DBS	Dried blood spot
DOT	Directly observed therapy
ELISA	Enzyme-linked immunoabsorbent assay
GIS	Geographical information system
GPS	Global positioning system
HFCA	Health catchment area
HEW	Health extension workers
HRP	High-risk population
IRB	Institutional Review Board
LLIN	Long-lasting insecticide treated bed net
IRS	Indoor residual spraying
MoHSS	Ministry of Health and Social Services
MEI	Malaria Elimination Initiative
MMP	Mobile and migrant populations
MRC	Multidisciplinary Research Centre
NVDCP	National Vector-borne Diseases Control Programme
PCR	Polymerase chain reaction
Pf	*Plasmodium falciparum*
PI	Principal Investigator
RACD	Reactive case detection
RDT	Rapid diagnostic test
SAE	Serious adverse event
SOP	Standard operating procedures

UCSF	University of California, San Francisco
UNAM	University of Namibia

2 Protocol summary

<table>
<tr><td>Title</td><td>Targeting malaria high-risk populations with tailored intervention packages: A study to assess feasibility and effectiveness in northern Namibia</td></tr>
<tr><td>Primary hypothesis</td><td>Targeted delivery of presumptive treatment and enhanced vector control will increase coverage of malaria interventions in high-risk populations and be more effective than standard of care case management and vector control in reducing malaria infection over one transmission season in Namibia.</td></tr>
<tr><td>Study design</td><td>An experimental matched pair pre/post design will be used to evaluate the feasibility and impact of targeted delivery of malaria interventions (including presumptive treatment and vector control interventions) against standard of care.

A total of 8 health facility catchment areas (HFCAs), 6 in Zambezi Region and 2 in Ohangwena Region, will be matched into pairs based upon population size and malaria risk criteria. Within each matched pair, HFCAs will be randomized into either the intervention or control arm.</td></tr>
<tr><td>Research Aims</td><td>Primary aims: To evaluate the feasibility and effectiveness of targeted delivery of malaria interventions to malaria high-risk populations (HRPs) (including presumptive treatment and enhanced vector control) for (1) increasing coverage of interventions and (2) reducing malaria parasite prevalence.

Secondary aims:

1. To calculate the association between confirmed malaria infection detected through the health system and receipt of an intervention within two regions in northern Namibia.
2. To evaluate the change in the health center catchment-level incidence of confirmed malaria infection within HRPs and the wider community, in intervention HFCAs vs control.
3. To evaluate the operational feasibility, acceptability of and adherence to targeted delivery of surveillance and interventions to high-risk populations.
4. To generate estimates of population size and turnover of known HRPs over a malaria transmission season.</td></tr>
</table>

	5. To describe *Anopheles* bionomic characteristics in agricultural areas, and evaluate potential efficacy of interventions to reduce vector density and biting.<br>6. To characterize the movement patterns of a sample of HRPs throughout one transmission season.<br>7. To improve surveillance capacity in HRPs and compare test positivity rates from targeted reactive case detection in high-risk agricultural worksites to routine reactive case detection in villages.
**Study site and target populations**	Total population of approximately 33,000 in 8 health center catchment areas (HFCAs) with major agricultural areas, within two regions in Namibia (Zambezi [6 HFCAs] and Ohangwena [2 HFCAs] Regions). Of this total, approximately 6,000 are estimated to be part of the target high-risk population groups (3,000 in intervention areas).<br><br>Based on preliminary data, the target HRPs are region-specific. In Zambezi Region, key HRPs are cattle herders and high-risk agricultural workers, while in Ohangwena Region only cattle herders who meet specified eligibility criteria as cross-border travelers to Angola will be targeted.
**Time frame**	October 2019-May 2020
**Intervention**	**Standard of care:** Control and intervention areas will receive the standard of care in Namibia: passive case detection through health facilities and health extension workers, routine indoor residual spraying (IRS), and reactive case detection (RACD) accompanied by reactive IRS.<br><br>**Test intervention:**<br><br>*Zambezi Region*<br><br>Targeted delivery of an intervention package to high-risk workers at farms and cattle posts in 3 of the 6 health catchment areas will include:<br><br>1. Presumptive treatment: all workers who regularly sleep overnight at farms/cattle posts will be presumptively treated with artemether lumefantrine (AL) in December/January (ploughing season) and again in March (early in the harvest season).<br>2. Targeted IRS: IRS with DDT (to traditional structures) or Deltamethrin (to modern structures) will be provided in December/January at farms or cattle posts that were not covered during the primary spray campaign (September to November).Tarps/tents will be sprayed with Deltramethrin.

	3. Vector control pack: LLIN for those who regularly sleep overnight at farms/cattle posts and do not sleep in a sprayed structure, plus a topical repellent and flashlight.<br>4. Education: Information on the causes, symptoms and prevention of malaria.<br><br>*Ohangwena Region*<br><br>Targeted delivery of an intervention package to cross-border cattle herders who are employed by owners in 1 of the 2 health catchment areas will include:<br><br>1. Presumptive treatment: all cattle herders who meet eligibility criteria as cross-border travelers will be presumptively treated with AL upon their return to Namibia between December to April, with maximum of two treatments separated by one month.<br>2. Targeted IRS: IRS with DDT (to traditional structures) or Deltamethrin (to modern structures) will be provided in December/January at cattle posts and kraals that were not covered during the primary spray campaign (September to November). Tents and tarps taken to Angola will be sprayed with Deltramethrin.<br>3. Vector control pack: LLIN for those who do not sleep in a sprayed structure in Angola, plus a topical repellent and flashlight.<br>4. Education: Information on the causes, symptoms and prevention of malaria.
**Primary outcome measures**	1. Self-reported coverage of any intervention among the targeted population, with IRS and LLIN measured pre-and post-intervention and presumptive treatment measured post-intervention only. Measures of intervention coverage include the proportion of individuals who, at any time during the study period: (i) presumptively received a full course of AL (presumptive treatment), (ii) slept at a worksite in a structure sprayed with insecticide during the past 6 months (IRS), and (iii) slept under a bednet (LLIN) the last night staying at a worksite.<br>2. Prevalence of infection among the targeted population using PCR
**Secondary outcome measures**	Effectiveness outcome measures<br>• Passively detected malaria cases confirmed by RDT or microscopy.<br>• Recent malaria exposure in HRPs as measured by test positivity by serological assays in cross-sectional surveys.<br>• Test positivity from RACD events, stratified by HRP classification.

	• Vector occurrence and densities, vector indoor resting density, vector biting time, vector biting location, human biting rate, human biting risk, and insecticide susceptibility status and frequency.<br><br>Operational feasibility and acceptability outcome measures<br>• Acceptance as evaluated by participation rate and by qualitative assessment.<br>• Costs of intervention and per case averted.<br>• Adherence as evaluated by pill count (presumptive treatment) and self-reported compliance (LLIN and topical repellents).<br>• Population size estimate of HRP groups<br>• Description of HRP movement over one transmission season
**Evaluation methods**	The primary outcome of intervention coverage will be assessed through cross-sectional surveys of HRPs at baseline and endline, using random sampling from census listings of worksites/employers. Similarly for parasite infection prevalence, at each cross-sectional survey, participants will be interviewed and tested for malaria using RDT, and DBS will be collected for molecular testing.<br><br>Secondary outcome evaluation methods:<br><br>Effectiveness assessment<br>1) A facility-based case-control study will be conducted to assess impact on odds of health facility-detected (passive) malaria cases.<br>2) Incidence in the HRP target group and non-HRP health catchment population will be estimated through collection of surveillance data through HEWs and health facilities, with expanded outreach through farm owners and cattle post owners in both intervention and control areas.<br>3) Past exposure will be assessed through seropositivity in the cross sectional surveys of HRPs will be assessed to compare past exposure in intervention and control areas.<br>4) Test positivity rates will be evaluated during routine RACD events and compare the proportion of individuals screened testing positive for malaria by RDT and PCR in HRP worksites to those in villages.<br>5) Vector assessments will be conducted alongside of cross-sectional surveys in Zambezi Region: vector indoor resting density will be assessed through pyrethrum spray catches (PSCs), and vector biting time and location, as well as human biting rates, will be measured through human landing catches (HLCs). Alongside the HLCs, human

	behavior observations (HBOs) will be carried out to describe exposure of humans to malaria vectors, i.e., human biting risk.<br><br>Operational feasibility and acceptability assessment<br>6) Acceptability will be assessed through quantitative measurement of study participation and through qualitative assessment in focus group discussions and key stakeholder interviews at endline.<br>7) Costing and cost-effectiveness data will be collected during study implementation in 2019/2020.<br>8) Adherence to AL and compliance to vector control interventions will be assessed by a pill count follow-up and use tracker log in a sample of participants, and self-reported measures for all interventions during the cross-sectional survey. Topical repellent containers will be weighed and LLIN condition assessed at specific timepoints.<br>9) Estimates of population size and turnover for HRPs will be evaluated through capture-recapture methods, including service delivery data, physical enumeration and census through farm and cattle post owners.<br>10) Spatial movement will be evaluated by GPS tracker devices provided to a cohort of cross-sectional participants at the baseline survey and incident cases, who are followed to the end of the study.
**Sample size**	A sample size of 930 participants in each arm is required to detect a 20% difference in the proportion of target population who used any intervention at a worksite between control and intervention arms, assuming coverage of 50% in control areas and a design effect of 6, with 90% power at the 5% significance level and allowing for 20% non-response. This sample size would provide 80% power to detect an absolute reduction of 5% in the prevalence of malaria between control and intervention arms, assuming a prevalence of 10% in the control arm, 5% significance and a design effect of 2. Based on the estimated size of the HRP population in each region, we will recruit 60% of the total sample from Zambezi Region and the remaining 40% from Ohangwena region.<br><br>Using preliminary information on farm/post size, we would need to sample approximately 112 farms/posts in each arm in Zambezi Region and 124 in each arm in Ohangwena Region. A probability-proportional-to-size calculation will determine how many of the farms/posts to include from each health catchment area to reach the total sample size in each arm.
**Statistical and analytical plan**	Three main approaches will be used to evaluate the impacts of targeted intervention delivery to HRPs. First, a difference-in-difference (DID)

	analysis will be conducted to evaluate the effect of the intervention package on coverage, malaria prevalence and malaria incidence in the population under treatment (average treatment effect on the treated). This approach compares outcomes in treatment and control arms at endline, adjusted for baseline differences measured pre-intervention. The analysis will be conducted within a regression framework using generalized linear models (GLMs), using binomial (for prevalence) and negative binomial (for incidence) distributions. The model will adjust for potential confounders (including seasonal variation and socio-demographics) and include a fixed effect to capture clustering at the health facility level.<br><br>Second, the protective effects of the intervention on malaria infection at endline will be estimated for individuals using multiple regression and adjusting for clustering at the health facility level. The protective effect of the intervention is expressed by the regression coefficient for exposure, i.e. the adjusted risk ratio of having a positive outcome in the intervention group versus in the control group.<br><br>Third, conditional logistic regression analyses will be conducted to estimate the odds of exposure to the intervention in cases versus controls, controlling for potential confounders at different scales. A fixed effect for health facility will be used to adjust for additional clustering at this level.

3 Study Personnel and Institutions

**Principal Investigators**	
Jennifer Smith, PhD<br>Assistant Professor, Epidemiology and Biostatistics<br>Malaria Elimination Initiative, Global Health Group, University of California, San Francisco<br>Email: Jennifer.Smith@ucsf.edu<br>Phone: +1-415-597-9247	Dr. Smith will be responsible for general leadership and oversight of the project. She will lead study design and protocol development, provide oversight of standard operating procedures in the field, supervisory visits and support interim and final data analysis and manuscript writing.
Roly Gosling, MD, PhD<br>Associate Professor, Dept. of Epidemiology and Biostatistics<br>Lead, Malaria Elimination Initiative, Global Health Group, University of California, San Francisco<br>Email: Roly.Gosling@ucsf.edu<br>Phone: +1-415-597-8114	Dr. Gosling will provide oversight of the project, with a focus on diagnostic and treatment approaches, and contribute to the study design, with technical input on interpretation of results and manuscript writing.
Dr. Davis Mumbengegwi<br>Deputy Director, and Head of Division of Science, Technology and Innovation, Multidisciplinary Research Centre, University of Namibia<br>Email: dmumbengegwi@unam.na<br>Phone: +264 (0) 61-206-3908	Based in Namibia, Dr. Mumbengegwi will provide oversight of laboratory procedures and analyses and support to study design and protocol development. He will ensure compliance with standard procedures, and contribute to data analysis and manuscript writing
**Investigators**	
Adam Bennett, PhD<br>Associate Director for Surveillance<br>Assistant Professor, Epidemiology and Biostatistics<br>Malaria Elimination Initiative, Global Health Group, University of California, San Francisco<br>Email: Adam.Bennett@ucsf.edu<br>Phone: +1 415 597 4981	Dr. Bennett is responsible for oversight of the project, and will contribute to the study design, protocol development and interim data checks. Dr. Bennett will provide technical input to the data analysis, interpretation and manuscript writing.
Henry Ntuku, MD, PhD<br>High Risk Populations Surveillance Specialist<br>Malaria Elimination Initiative,<br>Global Health Group<br>University of California, San Francisco<br>Email: henry.ntuku@ucglobalprograms.org	Based in Namibia, Dr. Ntuku will be responsible for overall coordination and day-to-day leadership and supervision of the project, including project administration, HR and in-country operations. He will also lead coordination with in-country partners and support study design, data analysis and manuscript writing.
Jerry Jacobson, PhD<br>Independent consultant<br>Email: jerryojacobson@gmail.com	Dr. Jacobson will lead the design and implementation of the population size estimation activities. He will provide technical support to the design of protocols, SOPs and data analysis related to population size estimation.
Dr. Michelle Hsiang, MD<br>Deputy Lead, Operational Research, Malaria Elimination Initiative, Global Health Group<br>Assistant Adjunct Professor, Pediatric Infectious Diseases, Dept of Pediatrics<br>University of California, San Francisco<br>Assistant Professor, Pediatric Infectious Diseases, Dept of Pediatrics, University of Texas Southwestern Medical Center<br>Email: Michelle.Hsiang@UTSouthwestern.edu	Dr. Hsiang will contribute to the project design, provide oversight with regards to laboratory data collection and management as well as technical and clinical input on diagnostic and treatment approaches. Dr. Hsiang will support data analysis and manuscript writing

Phone: +1-415-595-5978	
Immo Kleinschmidt, PhD<br>Professor of Epidemiology<br>Department of Infectious Disease Epidemiology, London School of Hygiene and Tropical Medicine<br>Email: immo.kleinschmidt@lshtm.ac.uk<br>Phone: +44(0)20 7927	Dr. Kleinschmidt will contribute to the targeted vector control aspects of the project, including experimental design, data collection, analysis, and manuscript preparation
Petrina Uusiku, MD<br>Chief Medical Officer, National Vector-borne Diseases Control Programme, Ministry of Health and Social Services<br>Email: uusikup@nacop.net<br>Phone: +264-811-462-707	Dr. Uusiku will support aspects of the project supported by the National Vector-borne Diseases Control Programme (NVDCP). She will provide input on design, coordination, and manuscript preparation. She will ensure that study findings inform future program planning and implementation as indicated.
Bryan Greenhouse, MD PhD<br>Associate Professor<br>Dept of Medicine / Div. of Infectious Diseases<br>University of California, San Francisco<br>Email: bryan.greenhouse@ucsf.edu<br>Phone: +1-415-206-8844	Dr. Greenhouse will lead genotyping and will work with Dr. Mumbengegwi and his lab to support polymerase chain reaction related aspects of the project. Additionally, he will contribute to overall study design, data analysis, and manuscript preparation.
Neil Lobo, PhD<br>Professor, University of Notre Dame, Notre Dame, IN<br>Malaria Elimination Initiative<br>Global Health Group, Institute for Global Health Sciences<br>University of California, San Francisco<br>Email: nlobo@nd.edu	Dr. Lobo will support entomology related aspects of the project. Additionally, he will contribute to overall study design, data analysis, and manuscript preparation. He will perform molecular entomological assays if required
Elodie Vajda, MS<br>Research Scientist<br>Malaria Elimination Initiative<br>Global Health Group, Institute for Global Health Sciences<br>University of California, San Francisco	Ms. Vajda will support the design, planning and capacity building for the vector assessment in Zambezi Region. Additionally, she will contribute to data analysis and manuscript preparation of resulting data.
Cara Smith Gueye, MPH PhD<br>Associate Director of Namibia Programs<br>Malaria Elimination Initiative<br>Global Health Group, Institute for Global Health Sciences<br>University of California, San Francisco	Dr. Smith Gueye will support the design, planning and coordination of the acceptability and feasibility evaluation (Ohangwena and Zambezi) and the vector assessment (Zambezi Region).
**Collaborators**	
Justine Petrus<br>Chief Environmental Health Officer<br>Ministry of Health and Social services, Ohangwena Region<br>Email : Justinepetrus@yahoo.com<br>Phone: +264 81 284 4064	Ms. Petrus will support project implementation through coordination of vector control activities and facilitating the relationship between the study and the Ohangwena regional health directorate.
Michael Lifasi<br>District Environmental Health Officer<br>Ministry of Health and Social services, Zambezi Region	Mr. Lifasi will support project implementation through coordination of vector control activities and facilitating the relationship between the study and the Zambezi regional health directorate.

Email : mmlifasi222@gmail.com<br>Phone: +264 81 856 6857	
Brooke Whittemore, MPH<br>Research Assistant<br>Department of Pediatrics<br>UT Southwestern Medical Center<br>Email: Brooke.Whittemore@UTSouthwestern.edu<br>Phone: +1-214-648-3561	Ms. Whittemore will provide administrative and logistical support, contribute to data management, data analysis, IRB submission and maintenance, and manuscript preparation.

4 Background

4.1 Malaria in high-risk populations

As malaria endemicity declines, transmission becomes focused in specific subgroups of populations, termed high-risk populations (HRPs), whose behaviors, occupations and socio demographic characteristics place them at higher risk of malaria infection, due to increased exposure to infectious mosquito bites [1]. These same populations frequently experience inequitable access to public health services, due to high mobility, migration status, language and cultural barriers or geographical remoteness of residences or worksites [2-4]. As a result, HRPs are more likely to be excluded from routine malaria surveillance systems and intervention campaigns, and therefore can contribute to sustaining malaria transmission dynamics or reintroducing infection after elimination is achieved. Achieving and maintaining malaria elimination will require a high coverage of effective interventions in HRPs, which in turn requires targeted and novel strategies to access and deliver tailored malaria surveillance and response [5].

Malaria programs lack systematic approaches to characterize HRPs and guidelines for adapting surveillance and response strategies to improve coverage in these populations. The Malaria Elimination Initiative (MEI) at the University of California San Francisco (UCSF) has developed "A Malaria Elimination Guide to Targeted Surveillance and Response in High-Risk Populations (HRP)" that provides National Malaria Control Programs (NMCPs) with a set of approaches to gather detailed epidemiological evidence on risk factors and behaviors of populations likely at high risk for malaria, adapt surveillance activities and improve targeting of interventions. This toolkit has been used in Namibia and other low-burden settings to help characterize malaria HRPs and identify opportunities to improve targeted surveillance and response.

4.2 HRPs in Namibia

A series of pilots in northern Namibia used a case-control methodology from the MEI HRP Toolkit to identify characteristics of high-risk populations in Ohangwena (2012-2014) and Zambezi Regions (2015-2016) [6]. These regions are respectively located in the north-central and north-eastern parts of Namibia, and have historically high levels of malaria transmission attributed to their tropical climate, remote location and high levels of cross-border movement with Angola and Zambia. Unpublished data identified specific populations at higher risk of malaria in Zambezi Region, including cattle herders, students, and individuals engaged in agricultural work during peak biting hours. In Ohangwena, male cross-border travelers were found to have the highest risk of malaria [6]. These populations are likely to be missed by routine surveillance and IRS campaigns, and tailored strategies for accessing them to improve coverage and impact transmission have not been developed.

To better understand the characteristics of previously identified HRPs and inform planning for targeted malaria surveillance and response strategies, the UCSF-MEI conducted a rapid formative assessment in both regions from October through December 2018 [7, 8]. This study identified high levels of indoor and outdoor exposure to mosquito bites in identified populations coupled with clear intervention gaps such as low coverage vector control measures and limited access to health services and associated barriers. Key barriers to access identified HRPs in the formative study include high mobility (particularly cross-

border movement), conflicting timing of malaria interventions and seasonal population movement, geographical remoteness of work-sites and fears around immigration status. In addition, there remains a lack of key data to inform intervention planning in these populations, including farm/cattle post numbers and locations, HRP population size, turnover, and infection levels. Given issues around access and lack of a clear sampling frame, intervention delivery strategies are likely to require a strong community-based approach to reach HRPs through gatekeepers, venue based and peer referral approaches. Finally, through a participatory approach, findings of the rapid formative assessment were workshopped in early 2019 with participants from the National Vector borne Disease Control Programme (NVDCP), the Regional Ministry of Health and Social Services (MOHSS) in both Ohangwena and Zambezi regions and local stakeholders in order to triangulate results, enrich interpretation and ensure recommendations are aligned with programmatic priorities. The target populations, delivery approach and intervention package evaluated in this study are directly informed by the final recommendations from the formative assessment.

4.3 Malaria interventions for HRPs in Namibia

HRPs in Namibia are at higher risk for malaria in part because of low coverage of routine interventions due to high mobility, remoteness or other barriers to seeking treatment (such as nationality) [7, 8]. Ideally, targeted strategies would build capacity around passive surveillance in these populations through existing systems as well as proactive approaches to identify and treat subclinical infections, fill gaps in IRS and provide alternative vector control interventions for those who do not live in sprayable structures. Given the limitations of currently available rapid diagnostic tests and microscopy that lack sensitivity in detecting asymptomatic infections in the community, targeted presumptive treatment (TPT) has been prioritized by the MoHSS for use in known HRPs.

Namibia's malaria control strategy has principally relied on malaria prevention through vector control using indoor residual spraying (IRS). IRS is conducted between September and November each year using Dichlorodiphenyltrichloroethane (DDT) on traditional structures, whilst deltamethrin is used on modern structures with cement-plastered surfaces. The country has also recently introduced pirimiphos-methyl (Actellic® 300 CS) for both traditional and modern structures. As is common in Southern Africa in areas with lower rainfall, the main vector in Namibia is *Anopheles arabiensis*. Preliminary results from ongoing entomological monitoring of susceptibility suggests that there is "possible" insecticide resistance to Deltamethrin in Zambezi and Ohangwena, and "possible" insecticide resistance to DDT developing in Zambezi [9]. However, these data are limited and resistance to any insecticide used in Namibia is assumed to be minimal in the proposed study areas. DDT-based IRS has served Namibia well as a method for preventing malaria and reducing transmission to low levels. However, the single round of IRS, as currently practiced, misses houses that are locked at the time of spraying. This includes structures in agricultural fields that are typically unoccupied until December/January, when the ploughing season begins, thus missing covering populations with high malaria risk with IRS, a key intervention.

Reactive case detection (RACD), i.e. focal screening and treatment of residents in neighboring households to passively detected cases is widely implemented in Namibia. However, due to low sensitivity of rapid diagnostic tests to detect low density infections and logistical challenges to achieving high coverage of those targeted for RACD, current evidence suggests that RACD has minimal impact on malaria transmission [10, 11]. Anecdotally, coverage is particularly low in HRP worksites in Namibia, which tend to be less accessible to program staff. Other strategies such as presumptive treatment might be more effective when targeted to populations known to be at high risk of infection. For this study, we

use artemether-lumefantrine (AL or Coartem), an artemisin combination therapy (ACT) that is the first line treatment for uncomplicated *Plasmodium falciparum* malaria in Namibia and has been approved for use in over 80 countries including the United States. AL has proven safe and effective against uncomplicated malaria and is well tolerated with minimal adverse effects [12]. A total of six doses are given over a three day period, and weight adjusted for children. Due to lack of data, AL is not indicated for children less than 5kg and women in the first trimester pregnancy. AL is effective against early stage gametocytes, providing the drug with some transmission blocking potential [13]. A randomized controlled trial conducted by the UCSF-MEI in Namibia has shown that reactive and focal presumptive treatment using AL, both alone and in combination with reactive IRS was effective in reducing infection prevalence and AL was safe with no severe adverse effects, well-tolerated, with high adherence and acceptability [14]. In a low transmission setting such as this, presumptive treatment with a short-acting drug such as AL is likely to have a greater impact on transmission than in a higher endemic setting due to the low probability of reinfection.

In addition to indoor exposures to mosquito bites, formative research in HRPs in Namibia identified numerous outdoor exposures in agricultural workers and cattle herders. These are related to occupational outdoor activities which may take place at night (such as ploughing or guarding cattle/crops) and social activities at farms and local bars. Entomological research conducted in Zambezi and Ohangwena regions suggest that outdoor biting occurs throughout the night, beginning in the early evening around 8pm [9]. There is a clear need to identify, pilot and evaluate the potential effectiveness of alternative vector control strategies which can be used to protect these populations from outdoor biting.

4.4 Targeted delivery of malaria interventions to HRPs

HRPs in Namibia tend to be missed by routine malaria interventions and have lower access to primary health care, and as a consequence require more targeted strategies to reach adequate coverage and improve case detection. To date, there is no experience conducting targeted delivery of malaria interventions to HRPs in Namibia or evidence of their impact, although limited net distributions were targeted to cattle herders present in villages in Ohangwena Region in May 2019. Other countries have demonstrated that strategies that improve community member ownership of distribution of interventions appear to have a large impact on increasing treatment coverage for neglected tropical diseases and malaria [15]. These strategies are increasingly used in the context of nomadic or otherwise mobile populations who may face greater barriers to accessing care.

Formative research in Namibia has highlighted the need to work collaboratively with farm and cattle post owners, through headman and other key gatekeepers, to maximize IRS coverage in agricultural areas during the spray season and conduct targeted interventions tailored to exposure profiles. Timing of interventions will vary in the two regions, due to the differing population movement of the targeted HRPs throughout the malaria transmission season. Importantly, many cattle herder subpopulations with posts located far into Angola will stay there from August until the rainy season in December-April. A few subgroups (such as bull-herders) may only return for cattle immunization clinics (timing varies), the festive bull competition (late May) and feeding the cattle after the harvest (May/June). As a consequence, intervention coverage is likely to vary substantially between subgroups.

5 Rationale

After a decade of tremendous decrease in malaria morbidity and mortality between 2001 and 2013 as a result of successful malaria control efforts, progress towards malaria elimination in Namibia has plateaued in the past few years. A number of outbreaks plagued the north of the country between 2016 and 2018, despite the large-scale application of IRS and reactive case detection as well as mass screening and treatment campaigns in high burden areas. This has prompted the country to shift its elimination target from 2020 to 2022 and highlights the need for new strategies to re-establish progress towards malaria elimination and expand coverage of surveillance and interventions to address existing gaps.

Over the last two years, the NVDCP of the Namibia MOHSS has completed the National Strategic Plan 2017-2022 (NSP), outlining strategies and activities and budget required to reach elimination by 2022. This revised strategy covers essential elimination strategies, including surveillance and response, case management, vector control, entomology, IEC/BCC, research and program management. In order to reach key targets of universal coverage of vector control interventions and treatment of malaria cases, the NSP has also recommended proactive surveillance and targeted LLIN distribution in high risk populations (HRPs), or those groups known to be at highest risk of malaria infection, who may have limited access to routine health services. However, the programme lacks resources and operational guidance to implement targeted strategies and evaluate their impact on intervention coverage and malaria transmission in HRPs and the wider community. In order to maximize sustainability and impact, targeted strategies will need to be community-led, build on existing health infrastructure and improve access to care in populations at highest risk of malaria.

5.1 Significance

The proposed operational research will evaluate targeted approaches for extending case management and malaria interventions to identified HRPs in northern Namibia, who have low access to routine health services and are believed to play a role in driving local transmission. The study addresses an urgent need for operational research on how to access those most distant from regular health services and quantify the added value of targeted surveillance and response in HRPs in terms of improving case detection and impact on malaria transmission.

In addition, the process and results support three core NVDCP strategies:

- **Improving response to malaria cases, identification of foci, and case-based reporting to support classification of all malaria cases and to prevent onward transmission from incident cases.** Increased access to case management and prevention in HRPs will be done in parallel with the MoHSS's goal of core surveillance system strengthening. Importantly, this trial will incorporate community-led approaches for expanding coverage to high-risk populations, including venue-based and peer-referral approaches.
- **Reaching the goal of universal coverage of IRS**, which is limited in areas of geographical remoteness and amongst highly mobile populations. The study will evaluate targeted vector control interventions intended to fill current gaps in coverage in HRPs, by measuring increases in coverage and impact on malaria transmission. The results will provide valuable evidence to donors and implementing partners to support integration of targeted delivery strategies into routine activities.

- **Increasing access to diagnosis and treatment of malaria together with delivery of health education**. This study will pilot new strategies for delivering these interventions to HRPs through a combination of venue-based and peer-referral strategies. In doing so, the project will build capacity of health extension workers (HEWs) who are a core resource for regional programmes.

6 Study aims

6.1 Primary aim

The **primary aim** of this project is to determine the feasibility and effectiveness of targeted delivery of malaria interventions (including presumptive treatment and enhanced vector control) to high-risk cattle herders and agricultural workers for improving intervention coverage and reducing the prevalence of malaria within the target population in northern Namibia.

6.2 Secondary aim

In addition, the following **secondary aims** will be addressed:
<u>Effectiveness</u>

- To calculate the association between confirmed malaria infection detected through the health system and receipt of an intervention within two regions in northern Namibia.
- To evaluate the change in the health center catchment-level incidence of confirmed malaria infection within HRPs and the wider community, due to intervention.
- To improve surveillance capacity in HRPs and compare test positivity rates from targeted reactive case detection in high-risk agricultural worksites compared to routine reactive case detection in villages.
- To describe *Anopheles* bionomic characteristics in agricultural areas, and evaluate potential efficacy of interventions to reduce vector density and biting

<u>Operational/Feasibility</u>

- To evaluate the operational feasibility, acceptability of and adherence to targeted delivery of interventions to high-risk populations.
- To generate estimates of population size and turnover of known HRPs over a malaria transmission season.
- To characterize the movement patterns of a sample of HRPs throughout one transmission season.

6.3 Primary research question and hypotheses

This project will aim to answer the following research question and hypotheses:
Does targeted delivery of malaria interventions (presumptive treatment and enhanced vector control) to HRPs increase intervention coverage and reduce malaria infection, compared to the standard of care (case management and vector control), over one transmission season in Namibia.

<u>Null hypothesis</u>: Compared to standard of care (routine case management through health facilities and health extension workers [HEWs] and vector control through regular spray campaigns), there is no

additional benefit of targeted delivery of malaria interventions to HRPs (including presumptive treatment and enhanced vector control), in increasing coverage of effective interventions and reducing malaria infection over one transmission season in Namibia.

<u>Research hypothesis</u>: Targeted delivery of presumptive treatment and enhanced vector control to HRPs will increase intervention coverage and reduce malaria infection over one transmission season in Namibia, compared to standard of care case management and vector control.

7 Study sites

Ohangwena and Zambezi Regions are respectively located in the north-central and north-eastern parts of Namibia, and have historically high levels of malaria transmission attributed to their tropical climate, remote location and high levels of cross-border movement with Angola and Zambia. In both regions, malaria is highly seasonal and typically is higher from January to June. Malaria is almost entirely due to *Plasmodium falciparum*. In 2018 (an outbreak year), Ohangwena reported 1,711 malaria cases for a population of approximately 281,358 translating into an incidence of 6 cases per 1,000 population, while Zambezi reported 3,354 cases for a population of 102,357, therefore the region had approximately 32 cases per 1,000 population in 2018. Peak periods of transmission corresponds with seasonal population movement between Namibia and neighboring malaria endemic countries, with Namibian cattle herders moving their herds back to Ohangwena Region from Angola and Zambian workers coming into Zambezi Region to herd cattle, plough and harvest crops. Both regions are largely rural, with significant agricultural activities in Okongo district in Ohangwena and western Zambezi Region.

The dominant vector in these regions remains *Anopheles arabiensis*, which has shown signs of possible resistance to insecticides in some regions and outdoor biting activity starting from 8pm [9]. Recent data from entomological surveys conducted in response to the 2017 malaria outbreak in Zambezi and Ohangwena Region suggests that vector composition may be complex and include *An. funestus* [16]. The main malaria interventions carried out by the MoHSS in the regions include case management, reactive case detection, and preseason annual blanket IRS campaigns with dichlorodiphenyltrichloroethane (DDT) or deltamethrin. In 2018, the reported IRS coverage in Ohangwena and Zambezi regions was 86% and 80% respectively. LLIN are distributed through universal campaigns directed to households and targeted distributions to vulnerable populations through antenatal clinics (children under 5 years and pregnant women). The last universal LLIN distribution occurred in 2015. In addition, reactive case detection (RACD) accompanied by reactive IRS are part of the standard of care in Namibia. RACD coverage varies in implementation according to case burden and capacity, but typically involves response to clusters of passively detected malaria cases.

Both regions have a designated malaria surveillance officer and a rapid case reporting system which utilizes DHIS2. UCSF has been collaborating with the NVDCP since 2014 to build surveillance and describe epidemiology of malaria in Zambezi Region. Activities have included establishment of rapid reporting systems in health facilities, a retrospective review of case data from 2012-2014, several cross-sectional surveys, a case-control study to identify high risk populations, pilot studies of network sampling methods and a trial of targeted parasite elimination (including presumptive treatment and targeted IRS around index cases).

The selected study sites within the two regions include agricultural areas within six health catchment areas with a historically high burden of malaria in the two regions (Table 1 and Figure 1). These areas

primarily border Angola (Ohangwena Region) and Angola/Zambia (Zambezi Region). Together, the population of the study area according to health catchment data is 33,000. Extrapolated data from a census of farms and cattle posts conducted pre-season indicate that there are approximately 700 farms/cattle posts that employ at least one worker within the six target HFCAs in Zambezi, and an estimated 800 cattle owners operating in the two HFCAs in Ohangwena Region. On average, there are four workers sleeping at the farms/cattle post in Zambezi during the transmission season and an average of three cattle herders per owner in Ohangwena. The majority of migrant workers are Namibian cattle herders in Ohangwena Region who graze their cattle in Angola and Zambian seasonal agricultural workers in Zambezi Region [7, 17].

All selected health facilities had at least 30 malaria cases in 2018. Selected health facilities are shown in Figure 1 and Table 1, in addition to the total number of villages and population in those selected areas, and the *P. falciparum* annual parasite index (API) for 2018. The anticipated time frame for the study is October 2019 – May 2020.

Figure 1. Selected health facilities in Zambezi and Ohangwena Regions

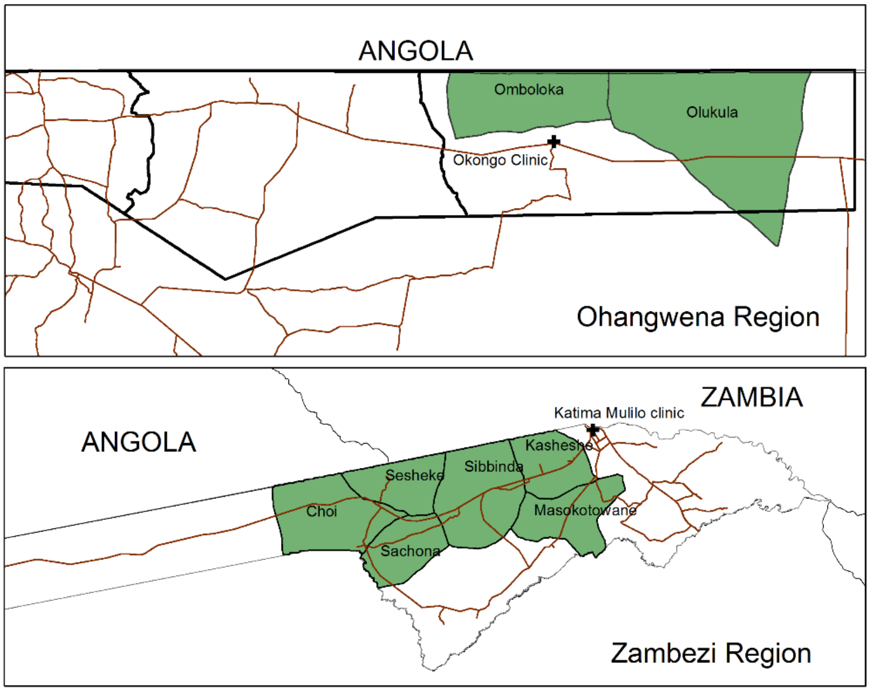

Table 1. Overview of incidence and population within selected health facility catchment areas[1] in Zambezi and Ohangwena Regions, January - December 2018

Region / District	Health facility	Population	API (per 1000)	HRP Population[2]
Zambezi	Choi	3587	51.9	480
Zambezi	Sesheke	5387	58.5	235
Zambezi	Sibbinda	4295	61.2	700
Zambezi	Kasheshe	2696	37.5	920

Zambezi	Masokotwane	2051	57.5	595
Zambezi	Sangwali	5006	30.8	575
Ohangwena / Okongo	Omboloka	5104	10.0	1485
Ohangwena / Okongo	Olukula	4891	8.0	1125
[1] Cases reported from Okongo clinic and Katima Mulilo clinic will be assigned to catchment of origin<br>[2] Based on reported number of farms/posts and average number of workers per farm/post				

8 Study Design

8.1 Study overview

To test these hypotheses, this study will employ an experimental, matched pair pre-post-intervention design comparing:

1. <u>Control:</u> Standard of care case management provided through existing health facilities and health extension workers; routine IRS conducted between September and November in villages with at least one reported case in the last three years and villages within ten kilometers of the Angolan or Zambian border, reactive case detection (RACD) and reactive IRS in households within 500m of passively detected cases.
2. <u>Intervention:</u> Targeted presumptive treatment conducted by HEWs in migrant agricultural workers and cattle herders, and enhanced vector control in agricultural areas (including IRS mop up and distribution of LLINs and topical repellent).

The primary outcomes measures to assess effectiveness include coverage of each intervention and PCR-based *P. falciparum* prevalence at endline. Secondary outcomes measures will include the odds of symptomatic malaria associated with receiving the intervention, *P. falciparum* confirmed case incidence over the study period, HRP population size, entomological outcomes, test positivity rates of targeted RACD, spatial mobility of HRPs and examine the operational feasibility, acceptability of and adherence to targeted testing and treatment by HEWs, presumptive treatment and enhanced vector control.

The study will be implemented between November 2019 and May 2020 in two regions in northern Namibia (Figure 2). Primary outcome measures will be assessed through two cross-sectional surveys at baseline (November/December 2019) and endline (March/May 2020) in the target population. Incident HRP cases and matched controls will be recruited continuously throughout the study for secondary outcomes around effectiveness. Endline qualitative studies will be conducted to assess feasibility and acceptability objectives.

Figure 2. Activity flow diagram

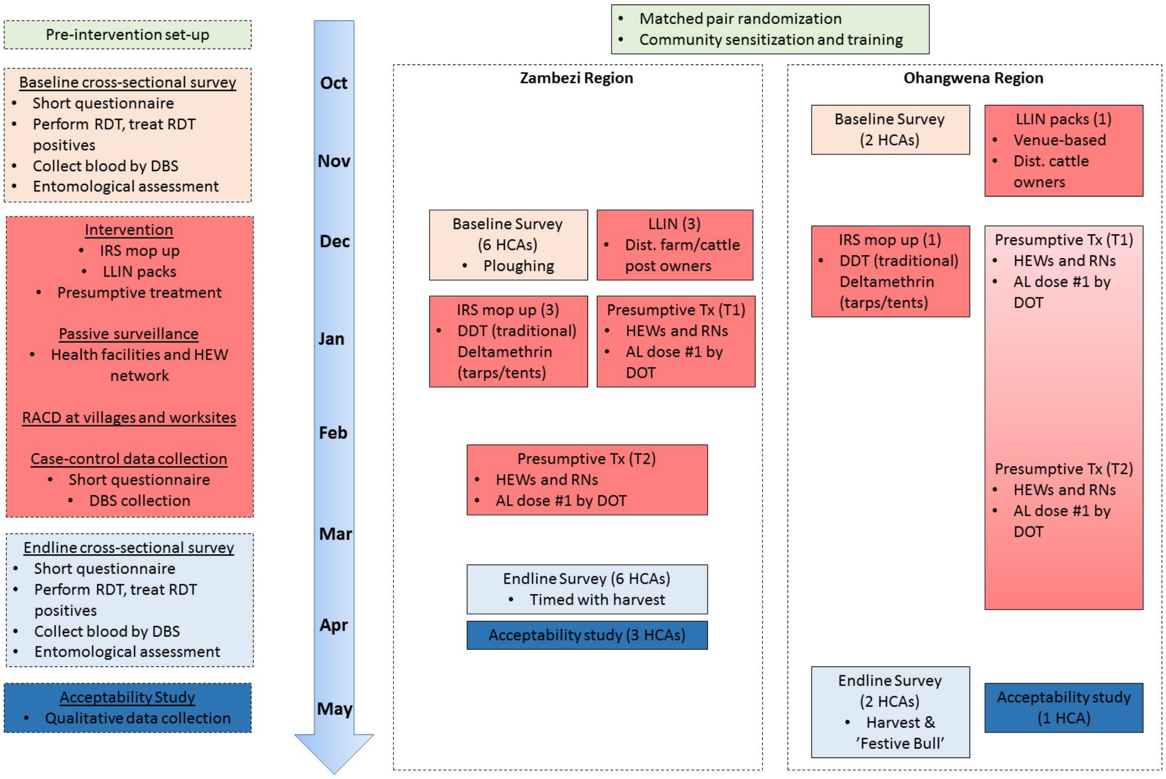

8.2 Study population

The study population includes agricultural workers and cattle herders in Zambezi Region and cross-border cattle herders in Ohangwena Region, who are employed in the selected health facility catchments. Inclusion and exclusion criteria for the interventions are described below.

<u>Inclusion and exclusion criteria</u>

Subjects must fulfill the following criteria to be eligible for **presumptive treatment**:

Inclusion criteria

- **Agricultural workers:**
    - At the time of intervention, slept regularly at a farm located within an intervention health facility catchment area over the past week or will do over the next three weeks.

- **Cattle herders:**

At the time of intervention, either:

- Works for a cattle post owner based in an intervention health facility catchment area

- **All participants:**
    - Willing and available to participate in the study
- Informed consent for participants under the age of 18 years will be provided by the parent or guardian. Minors 12-18 years of age will be asked for written assent.

- If the number of seasonal workers is unexpectedly high, presumptive treatment in Zambezi Region will be restricted to participants reporting travel outside of Namibia in the last 60 days

Exclusion criteria:
- **All HRP members:**
    - Pregnancy in the first trimester
    - Previous regular menstruation with no menstruation for most recent four weeks
    - Weight < 5 kg
    - Severe malaria
    - Known AL allergy
    - Received presumptive treatment with AL within the past 3 weeks

Subjects must fulfill the following inclusion criteria to be eligible for **enhanced vector control (LLIN or sprayed tent/tarp, topical repellent)**:
Inclusion criteria
- **Agricultural workers:**
    - At the time of intervention, slept regularly at a farm located within an intervention health facility catchment area over the past week or will do over the next three weeks, and
    - At the farm location, does not sleep in a structure sprayed with insecticide
- **Cattle herders:**
    - Works for a cattle post owner based in an intervention health facility catchment area, and
    - At the cattle post, does not sleep in a structure sprayed with insecticide, and
    - Travels to Angola during the malaria transmission season [*Ohangwena only*]
- **All participants:**
    - Willing and available to participate in the study
- Informed consent for participants under the age of 18 years will be provided by the parent or guardian. Minors 12-18 years of age will be asked for written assent.

8.3 Outcome measures

Table 2. Outcome measures

**Outcomes**	**Indicator**
Primary Aims	
Effective coverage	Self-reported coverage of any intervention among the targeted population, with IRS and LLIN measured pre-and post intervention and presumptive treatment measured post-intervention only. Measures of intervention coverage include the proportion of individuals who, at any time during the study period: (i) presumptively received a full course of AL (presumptive treatment), (ii) slept at a worksite in a structure sprayed with insecticide during the past 6 months (IRS), and (iii) slept under a bednet (LLIN) the last night staying at a worksite. In addition, for IRS, LLIN and topical repellents, proportion of person-months over which eligible people were protected at a worksite out of all eligible person-months, measured during each cross-sectional survey through a series of survey questions about receipt and use of the intervention.
Infection prevalence	Proportion of individuals with malaria infection (detected by PCR) out of all individuals tested within the baseline and endline surveys.
Secondary Aims	
Effectiveness	

Odds of symptomatic malaria	The odds of exposure (receiving each intervention) in incident confirmed HRP malaria cases detected at health facilities or HEWs relative to population controls, matched by neighborhood area and restricted to HRP eligibility.
Incidence	Cumulative incidence of confirmed malaria cases over the study period in the target population and community, at the health facility level.
Exposure	Seroprevalence of infection, among all age groups, as measured by ELISA.
RACD test positivity	Proportion of individuals screened by RDT and PCR during RACD events, stratified by HRP status and location type (village or work site).
Feasibility/Operational	
Operational program coverage	Proportion of individuals and farms visited and offered the interventions within the target areas.
Adherence	Proportion of people who complete the course of AL among a subset of people initiated who are randomly selected to receive modified DOT, as assessed by pill count; proportion of people who report use of LLIN and topical repellents over a 30 day period, as assessed by a daily reporting log; proportion of people who report compliance to vector control interventions and AL adherence at endline.
Acceptability	Quantitative assessment (proportion agreeing to receive each intervention, proportion indicating they would participate in future interventions at endline cross sectional survey) and qualitative assessment.
Cost	Cost per case adverted.
Population size & turnover	Population size estimate and turnover between seasons.
Vector characteristics	Species composition using morphology; Vector density by collection methods, period and collection method; Temporal correlation of biting times with human exposure patterns.
Human movement patterns	GPS movement tracts over one transmission season of a representative sub-sample of cross-sectional participants and incident cases.

8.4 Matching and randomization

A total of 8 health center catchment areas (HFCAs) in Zambezi Region and Ohangwena Region were selected for inclusion based upon (i) HRP population, (ii) proximity to the border, and (iii) historical malaria incidence per 1000 population between January and December 2018. HFCAs will be matched into pairs following the baseline cross-sectional survey and enumeration activities, based upon location (region and distance to the border), historical incidence per 1,000 (high/low) over the prior year, high-risk population size (high/low), prevalence of malaria by RDT at baseline, and presence of specific sub-groups such as laborers harvesting Devil's Claw (presence/absence).

Within each matched pair, HFCAs will be randomized into either the intervention or control arm so that 4 HFCAs are allocated to each group. While this randomization will not ensure balance on potential confounding factors, due to the small sample size, it avoids major biases in subjective allocation. The 'sample' command in Stata v14 (StataCorp, College Station, TX) will be used to implement randomization.

8.5 Sample size

Coverage of malaria interventions

Current routine IRS coverage in Zambezi and Ohangwena regions is reported to reach at least 80% of the population and a cross-sectional survey reported a coverage of 71% in western Zambezi Region. We expect coverage to be lower in agricultural areas, based on information collected in the formative

assessment. A sample size of 930 participants in each arm is required to detect a 20% difference in the proportion of target population who used any intervention at a worksite between control and intervention arms, assuming coverage of 50% in control areas and a design effect of 6, with 90% power at the 5% significance level and allowing for 20% non-response. This sample size would provide 80% power to detect an absolute reduction of 5% in the prevalence of malaria between control and intervention arms, assuming a prevalence of 10% in the control arm, 5% significance and a design effect of 2. Based on the estimated size of the HRP population in each region, we will recruit 60% of the sample from Zambezi Region and the remaining 40% from Ohangwena region.

The cross-sectional sampling strategy will be based on a census listing of farms and cattle posts conducted pre-season to determine the number of locations and workers employed. Preliminary data from Zambezi Region show that there are 700 farms or posts that employ at least one seasonal worker within the six target health catchment areas and nearly 800 cattle owners in the two catchment areas in Ohangwena Region. Based on 5 workers on average are present on each farm/post in Zambezi Region and 3 employed by cattle owners in Ohangwena Region, we would need to sample approximately 112 farms/posts in each arm in Zambezi Region and 124 in each arm in Ohangwena Region. Using this information, a probability-proportional-to-size calculation will determine how many of the farms/posts to include from each health catchment area to reach the total sample size in each arm.

HRP-level malaria parasite prevalence

The above sample size would provide 80% power to detect an absolute reduction of 5% in the prevalence of malaria between control and intervention arms, assuming a baseline prevalence of 10% and 5% significance and a design effect of 2.

9 Study Interventions

9.1 Overview

Interventions will be conducted by HEWs and study staff in coordination with farm and cattle post owners, with support from traditional authorities and Regional MoHSS partners.

9.1.1 Community sensitization and understanding

We have already secured commitment from National and Regional MoHSS partners during the formative assessment and workshops leading up to development of this protocol. Prior to starting the study, we plan to build awareness and encourage participation at the local (community) levels and continue to build strong links and coordinate through the Regional MoHSS. Once the protocol has been approved by all stakeholders, we will sensitize health facility staff and health extension workers for malaria, including meeting them at health facilities and providing information about study activities. We will work with HEWs and surveillance teams to promote "expanded passive surveillance" to all HRPs through a referral network involving farm and cattle post owners.

Each health facility catchment included in the study will be assigned to intervention or standard of care (which both include expanded passive surveillance) before the study begins. Sensitization of farms and

cattle posts in intervention areas will occur before the study begins, using a “top down” approach which relies on communication through local councilors, village headman, and farm and cattle post owners. This is necessary due to potential sensitivities around immigration status for migrant workers in Namibia and high cross-border mobility of cattle herders in Ohangwena.

9.1.2 Targeted reactive case detection (RACD)

Although RACD is already part of the standard of care in Namibia, coverage varies in implementation according to case burden and capacity and typically involves response to clusters of passively detected malaria cases. Anecdotally, coverage is reported to be particularly low in agricultural worksites due to their geographical remoteness and limited capacity of the program to respond to all cases. RACD at worksites for eligible index cases who are members of a target HRP group (as outlined in Section 8.2 on ‘Study Population’) will be supported by the study team within all 8 HFCAs, following standard operating procedures outlined below:

- In the absence of consistent real time tablet-based reporting, nurses at HF report malaria cases at real time through a WhatsApp platform (sharing a picture of the case notification form with the RDT cassette) managed by the MOHSS surveillance Officer.
- The study field supervisor will be added to the WhatsApp platform to monitor incoming cases.
- At the beginning of the study, nurses at selected HF will be sensitized to add a comment on the picture when submitting an HRP malaria index case.
- The study field supervisor will also regularly consult the MOHSS surveillance officer for any reported case, member of a target HRP from the selected HF
- Within 3 days of reporting, the field supervisor will call the index case to organize a time and a day of team visit at the worksite, coordinating the visit closely with the farm/cattle owner
- If a phone call is not possible, a driver will visit the farm/cattle post to arrange the team visit
- Within 7 days of reporting a study team will visit the worksite at the time and day previously agreed to conduct RACD

At the worksite, all individuals who slept at the farm/cattle post the previous night will be screened using RDTs.

If found positive by RDT, treatment will be administered as in Table 3. Per national policy, a single low dose of primaquine (0.25mg/kg of weight) will be administered to study participants on the first day of treatment in addition to AL (Appendix 5). Contraindications to primaquine include pregnancy (any trimester), age less than 12 months, infants weighing less than 10 kilograms, women in the first 12 months of breastfeeding, and prior allergic reaction to primaquine. Participants with contraindications to primaquine will not be excluded from the study. Women of reproductive age will be assessed for treatment as in Section 9.1.3.

9.1.3 Presumptive treatment

Presumptive treatment of eligible HRPs will occur at two set time points, coinciding with key agricultural seasons (Zambezi Region) and peak cross-border mobility (Ohangwena Region). Due to differing movement patterns in each region and availability of HRPs in Namibia, the logistics and timing of AL distribution will differ. A combination of peer-referral and venue-based approaches will be used to

maximize intervention coverage within target populations in each region. The intervention team will include a field investigator, a driver, a HEW and a supervising nurse. Both field investigator and driver will also support targeted IRS in the same locality.

The following distribution strategy will be used in **Zambezi Region**:

- Timing and logistics of distribution will be closely coordinated with farm/cattle post owners and occur at central gathering points (i.e. water points for clusters of farms/cattle posts).
- Owners will inform employees of dates and times of drug distribution in advance.
- HEWs will obtain a register of eligible workers at each farm in advance and use this list to calculate a preliminary estimate of coverage based on distribution data.
- Distribution will occur over a week period in each catchment area, with a minimum of two days spent at each distribution point. All recipients on day 1 will be asked to invite others working at farms within the catchment area and who meet eligibility criteria in Section 8.2 to attend on day 2.

The following distribution strategy will be used in **Ohangwena Region**:

- Owners will inform employees that they will receive presumptive treatment upon their return from Angola, up to a maximum of two distributions between December 2019 and March 2020 and separated by at least 30 days.
- Timing and logistics of distributions will be closely coordinated with cattle post owners and occur at central gathering points (i.e. water points) by HEWs over multiple one week periods and at health facilities for other returning cattle herders.
- HEWs will obtain a register of eligible cattle herders associated with owners in each village in advance and use this list to calculate preliminary estimates of coverage based on distribution data.

HEWs will treat all eligible individuals with an age-appropriate course of artemether-lumefantrine (AL), which is currently the first line drug used for uncomplicated malaria in Namibia. AL requires two daily doses for three consecutive days, for a total of six doses (Appendix 2). The first antimalarial dose will be delivered by DOT and subsequent doses will be left with the subject, with instructions to self-administer them.

AL will be dosed per manufacturer guidelines, with weight-based dosing for children described in Table 3 below.

Table 3. AL weight-based dosing

Body weight (kg)	Tablet strength (mg)		Tablets/ dose	Mg of drug per dose		Tablets/day
	Artemether	Lumefantrine		Artemether	Lumefantrine	
5 to 14	20	120	1	20	120	1 tablet given twice* per day for 3 consecutive days
15 to 24	20	120	2	40	240	2 tablets given twice* per day for 3 consecutive days
25 to 34	20	120	3	60	360	3 tablets given twice* per day for 3 consecutive days
35 or greater	20	120	4	80	480	4 tablets given twice* per day for 3 consecutive days
*Approx. 8 hours between doses 1 and 2. Approx. 12 hours between all other consecutive doses.						

Per national guidelines in Namibia, AL will not be given to women who are pregnant in the first trimester, individuals weighing less than 5kg, those with a known AL allergy or suspected severe malaria. All women of reproductive age (15-49 years) will be asked about menses. Those who have reached menarche but have not menstruated in the past four weeks will be offered a pregnancy test. Any woman who has not had menses in the past four weeks and refuses to take a pregnancy test will be excluded from receiving AL. Any person excluded from AL will be offered a malaria RDT during the intervention. Subjects testing positive by RDT during the intervention will be referred to the closest health facility for treatment with a drug that is safe for them according to national policy, e.g. oral quinine for uncomplicated malaria in the first trimester.

All individuals with suspected severe malaria or other severe illness (including those with symptoms of severe anemia, prostration, impaired consciousness, respiratory distress, convulsions, circulatory collapse, abnormal bleeding, jaundice or passing dark urine) will be referred to the nearest health facility for clinical assessment and treatment.

9.1.4 Targeted Indoor Residual Spraying (IRS)

A mop up indoor residual spraying (IRS) campaign will be targeted to farms and cattle posts/kraals in intervention areas in December 2019 to fill gaps from the routine spray campaign (September to November 2019). This campaign will immediately follow the baseline cross-sectional survey and coincide with presumptive treatment in Zambezi Region. The spray team will include a lead spray operator (who will act as the data collector during the cross-sectional survey) and an assistance spray operator (who will be the driver). Spray teams and drug distribution teams (HEWs and nurse) will travel together and operate in the same cluster of farms/cattle posts during a given day.

IRS will be planned and targeted based on a self-reported census of sleeping structures on farms and cattle posts undertaken in April 2019 as well as information provided by the MoHSS routine spray campaign. All sprayable sleeping structures on farms and cattle posts in intervention areas will be eligible for spraying if they were not done during the routine IRS campaign. The team will spray each structure with the recommended solution of dichloro-diphenyl-trichloroethane (DDT) for traditional structures and Deltamethrin for modern structures, tarps and tents.

On the day prior to spraying, the spray personnel will call farm/cattle post owners to remind them of these activities and ask them to be present on the day and ensure that all occupants are made aware of the purpose of spraying and what is required. This will allow any occupants time to prepare and vacate the structure before spraying, as we expect workers to be in the fields/watching cattle during much of the day. This will require moving any household items in advance, including water, food, cooking utensils and toys from the house. When possible, furniture should be moved outside; if this is not possible, it should be moved to the middle of the room to allow easy access for spraying walls and covered. All walls and ceilings of all sleeping structures will be sprayed according to NVDCP guidelines at an application rate of 40 ml/sq m with a Hudson X-pert sprayer (H D Hudson Manufacturing Company, Chicago, Ill, USA). Rooms will not be sprayed if people or animals are present, or if household items are not correctly removed or positioned. It is estimated that the IRS procedure will take approximately 20 minutes per structure as these are not permanent homes and there should be a minimum of objects.

Owners will be allowed back into the structure after spraying and will be asked to mop any excess solution from the floors and wash the floors with water before allowing any young children back into the house. Owners will be reminded not to wash, paint of re-plaster sprayed walls at least until the end of the malaria transmission season.

9.1.5 LLIN distribution and/or vector control packs (sprayed tents/tarps and topical repellent)

Alternative vector control interventions, including LLINs, sprayed tents/tarps and topical repellents will be distributed to eligible HRPs between October and January 2019 and coordinated closely with farm and cattle post owners. Distribution strategies will differ between the two regions, based on HRP movement patterns.

In Zambezi Region, HEWs will distribute LLINs and/or vector control packs (including an LLIN, spraying of tents/tarps and topical repellent) to workers who are present at the farm/cattle post in December. Distribution will be timed to coincide with presumptive treatment and mop up IRS, following the baseline cross-sectional survey. Standard IRS Spray methodologies will be adapted for spraying tarps/tents. All intervention products will be marked with a unique identification code that is registered to a specific individual and farm/cattle post, with the aim that they are provided for use while the individual is employed at the farm. After the initial distribution, farm/cattle post owners will be instructed to notify the HEW of all new eligible workers arriving at the venue throughout the study period, so that they may provide an LLIN/repellent and track distribution. During the second round of presumptive treatment, during the harvest season, HEWs will conduct a stock checks to ensure that the LLINs in stock/use correspond to the number registered to a given farm/cattle post, reallocate LLINs in circulation and provide additional LLINs or repellent to workers as needed. We expect numbers in the harvest season to be higher than the ploughing season.

In Ohangwena Region, distribution of vector control packs (including an LLIN, spraying of tents/tarps and topical repellent provided in a backpack) will be timed to coincide with the baseline cross-sectional survey. Sensitization and coordination will be done through a line listing of cattle post owners and traditional authorities, but pack distribution will be done at water points and other popular venues. All intervention products will be marked with a unique identification code that is registered to a specific individual and cattle post owner, and cattle herders will be instructed to carry and use the LLIN and repellent while they are in Angola.

9.1.6 AL adherence and safety monitoring

There are limited data on the expected adherence to three-day regimen among asymptomatic people, but a recent study of targeted parasite elimination (TPE) in households surrounding index cases in Zambezi Region found that adherence to presumptive treatment was 100% (339/339) among individuals who still had their blister packs [14]. Among symptomatic people, the reported adherence ranges from 39% to 100% [18]. To ensure participants' adherence to their medication and monitor safety, participants will be informed on how to take their medications, about the side effects of the treatment and the importance of compliance to the treatment. Participants with phone numbers will be sent a daily text message to remind them (and their eligible colleagues) to adhere to their treatment.

The safety risks associated with participation in the intervention are expected to be minimal, as AL is well tolerated and safe. The common adverse events reported for AL are: headache, dizziness, loss of appetite, generalized weakness, fever, chills, arthralgia, myalgia, nausea, vomiting, and abdominal pain.

Identification of serious adverse events (SAEs) will occur passively and actively. As part of the consent process, participants will be instructed to call or visit the health facility/HEWs to report any adverse events, who will refer all SAEs to designated health facilities for management. During adherence and follow up visit, the HEWs will also ask participants for specific adverse events. The HEWs will report all adverse events to the supervising nurse. For all SAEs, the supervising nurse will discuss management with the local Physician, serving as Local Safety Monitor, who will advise whether the participant should continue or stop treatment due to safety concerns. SAEs as well as serious unexpected serious adverse reactions (SUSAR) will be reported to the study management and PI who will report to the Pharmacovigilance center of the Ministry of Health and the respective IRBs.

10 Study evaluation methods

10.1 Overview of evaluation activities by study aim, research objectives, outcomes, and data collection activities

Study activity	Research objective	Outcome measures	Data collection activity
Activity 1: Impact evaluation of targeted delivery of malaria interventions (presumptive treatment and enhanced vector control)	1. Evaluate the increase in intervention coverage in malaria HRPs and reduction in malaria prevalence in intervention areas with targeted delivery, compared to standard of care<br><br>**[Primary]**	Effective coverage of malaria interventions (proportion of individuals that report use of each intervention at a worksite over the study period); proportion of person-months covered by each intervention at a worksite.	Cross-sectional survey November/December 2019 and April/May 2020<br><br>Interventions distribution data
		Malaria prevalence in HRPs	Cross-sectional survey November/December 2019 and April/May 2020<br><br>Routine programmatic data on LLIN, IRS and RACD
	2. Evaluate the association between symptomatic malaria and receipt of an intervention<br><br>**[Secondary]**	Odds of symptomatic malaria associated with receiving each intervention	Health facility-based case-control study in HRPs within study areas
	3. Evaluate the reduction in malaria exposure in HRPs in intervention areas with targeted delivery, compared to standard of care<br><br>**[Secondary]**	Malaria seroprevalence in HRPs	Cross-sectional survey November/December 2019 and April/May 2020
	4. Evaluate the reduction in malaria transmission in HRPs and the wider community in intervention areas with targeted delivery, compared to standard of care.<br><br>**[Secondary]**	Total confirmed outpatient (OPD) malaria case incidence by health facility, stratified by HRP status	Routine malaria data from all reporting HEWs, health centers and district hospitals
Activity 2: Assessment of the feasibility, acceptability of, and adherence to targeted presumptive treatment and	1. Assess the operational feasibility of targeted delivery of presumptive treatment, LLINs and IRS to HRPs	Operational program coverage (proportion of individuals and farms/cattle posts visited and offered and accepted the interventions within the target areas)	Intervention distribution data
		Proportion of HEWs who rate conducting the interventions as very easy, somewhat easy,	Quantitative entrance, mid-intervention, and exit interviews with HEWs; Focus

enhanced vector control	**[Secondary]**	somewhat difficult, and very difficult, and changes in proportions over time; Qualitative feedback on operational feasibility.	group discussion with HEWs at end-line
		Costs of each intervention as unit and total costs; cost-effectiveness will be assessed as an incremental cost effectiveness ratio (ICER)	Program data on costs combined with estimates of program effectiveness
	2. Assess the acceptability of targeted delivery of presumptive treatment, LLINs and IRS to HRPs<br><br>**[Secondary]**	Proportion of targeted individuals refusing each intervention	Intervention distribution data
		Proportion of survey respondents who strongly disagree, disagree, are ambivalent, agree and strongly agree on the importance and acceptability of each intervention; Qualitative feedback on acceptability of interventions.	Cross-sectional survey questions for employers and HRPs; Qualitative studies at endline with HRPs, HEWs and other health sector staff and employers; Entrance, mid-term and Exit Interviews with HEWs
	3. Assess treatment adherence and VC intervention compliance<br><br>**[Secondary]**	Proportion of people who complete all pills in the pill pack after receiving the first dose of AL by DOT	Pill count for sub-sample of study participants.
		Proportion of people who self-report completion of all pills in the pill pack at each time point.	Cross-sectional survey at endline.
		Proportion of person-days who self-report use of LLINs and topical repellent over a 30 day period.	Daily use tracking log
Activity 3: Surveillance capacity building and assessment of targeted reactive case detection	1. To assess the difference in test positivity rates from targeted reactive case detection in high-risk agricultural worksites compared to village settings.<br><br>**[Secondary]**	Test positivity rates from RACD, stratified by HRP status and location (village or worksite)	Routine RACD data collection, facilitated by the study team in HRP worksites.
Activity 4: Population size estimation and turnover assessment	1. Quantify the population size and seasonal turnover in HRP groups<br><br>**[Secondary]**	Population size estimate and turnover between seasons	Service delivery data, cross-sectional surveys and census through farm/cattle post owners
Activity 5: Vector assessment in agricultural areas in Zambezi Region	1. To assess the vector composition, density and behavior in agricultural sites and correlate with human exposure patterns<br><br>**[Secondary]**	Species composition (i.e., vector occurrence and densities) using morphology; vector indoor resting density by PSCs; biting time and location, human biting rates, by HLCs; human biting risk by combining HLCs with HBOs.<br><br>Insecticide resistance status and frequency will also be evaluated.	Vector assessments using human-landing captures (with concurrent HBOs)and PSCs, timed to coincide with cross-sectional surveys in December 2019 and April 2020
Activity 6: GPS logger movement study	1. To characterize fine-scale movement patterns of HRPs over one transmission season	Proportion of individual's time spent in different locations (farm, cattle post, village) and temporal movement patterns	GPS logger data collection through time in country; monthly movement surveys

	**[Secondary]**		
		Distance and frequency of travel during and between agricultural seasons	GPS logger data collection through time in country; monthly movement surveys

10.2 Impact evaluation of targeted delivery of malaria interventions (presumptive treatment and enhanced vector control)

10.2.1 Outcome measures

<u>Outcome 1 (Primary): Effective coverage of malaria interventions</u>
The primary coverage metric will be estimated at the individual level as the proportion of individuals that directly benefited from any targeted intervention among all individuals eligible for inclusion. This will be obtained from cross-sectional data separately measuring the proportion of individuals who, at any time during the study period: (i) presumptively received a full course of AL (presumptive treatment), (ii) slept at a worksite in a structure sprayed with insecticide during the past 6 months (IRS), and (iii) slept under a bednet (LLIN) the last night staying at a worksite. In addition, for IRS, LLIN and topical repellents, the proportion of person-months over which eligible people were protected out of all eligible person-months, will be measured during each cross-sectional survey through a series of survey questions about receipt and use of the intervention. The difference in intervention coverage between arms will be assessed using a difference-in-difference approach for IRS and LLIN and the protective effect of the intervention on malaria infection at endline estimated using multiple regression.

<u>Outcome 2 (Primary): Malaria prevalence in HRPs</u>
Species-specific prevalence of malaria infection will be calculated as the proportion of people testing positive for each species malaria by PCR out of all tested HRPs. The reduction in malaria prevalence will be assessed using a difference-in-difference approach, comparing change (pre- versus post-intervention) in all-species infection between intervention and control groups.

<u>Outcome 3 (Secondary): Odds of symptomatic malaria associated with receiving each intervention</u>
The odds of clinical malaria associated with receiving each intervention will be measured by comparing the risk of exposure in cases (RDT-positive) to the risk in controls (RDT-negative) in HRPs within study areas.

<u>Outcome 4 (Secondary): Malaria seroprevalence in HRPs</u>
Malaria exposure in HRPs will be measured as the proportion of people with antimalarial antibodies present in their blood serum. ELISA assays will be used to detect biomarkers of *Pf* exposure, using collected dried blood spots during baseline and endline cross-sectional surveys. The reduction in malaria exposure will be assessed using a difference-in-difference approach, comparing change (pre-versus post-intervention) between intervention and control groups.

<u>Outcome 5 (Secondary): Total confirmed outpatient (OPD) malaria case incidence by health facility, stratified by HRP status</u>

The total confirmed outpatient malaria case incidence will be estimated for each of the study health catchment areas and stratified by HRP status. RDTs used for routine malaria diagnosis will be collected and a short questionnaire affixed to the back to allow collection of key indicators to stratify incidence estimates. Denominators will be based on health catchment populations and population size estimates obtained through this study. A reduction in malaria incidence in HRPs and the overall catchment population will be evaluated using a difference-in-difference approach, comparing change (pre- versus post-intervention) between intervention and control groups.

10.2.2 Sample size

Sample size calculations for the primary outcomes are included under Section 8.5.

Case-control study

Based on an estimated HRP population size of 6,000 within the entire study area and an incidence of 30 per 1000 in this population, we expect to recruit 180 HRP cases over the study duration. Based on this sample size, the minimum detectable effect is a 50% reduction in the odds of malaria amongst those who reported receiving the intervention, assuming a two-sided α of 0.05, power of 0.80, an estimate of 50% controls exposed (ie, received the intervention), and a ratio of one case to three controls. This will require recruitment of a total of 540 HRP controls, who will be recruited from the neighborhood of HRP index cases during targeted RACD.

10.2.3 Data collection

Cross-sectional surveys

A baseline household survey will be conducted at the beginning of the study in HFCAs to assess baseline demographics, current intervention coverage, treatment seeking, and mobility including cross-border travel. An endline survey will be conducted a month after the second AL distribution and at near the end of the transmission season to obtain an unbiased estimate of intervention coverage and malaria parasite prevalence in each study arm. Both surveys will include testing with RDTs and collection of DBS for PCR-based testing.

Timing of surveys

Timing of the baseline and endline surveys will differ in Ohangwena and Zambezi Regions, given the differing timing of HRP movement. Actual timelines may vary slightly in an effort to align the surveys with arrival of the rainy season, which largely dictates seasonal activities and population movement.

In Ohangwena, the baseline cross-sectional survey will be carried out as early as possible, to ensure high LLIN coverage prior to the start of the malaria transmission season in Angola. The study population will include those near the Angolan border as well as those who stay in Angola for longer periods of time, who will be informed about the survey through their employers. This event will also be an opportunity to distribute vector control packs, which may provide an added incentive to return to Namibia and participate. The endline cross-sectional survey in Ohangwena will be held near the end of May, when the majority of cattle herders are present in Namibia and allow the cattle to graze on the left over harvest as well as attend the “Festive Bull Competition”.

Appendix S4 – Study protocol

In Zambezi Region, the baseline cross-sectional survey will be conducted in December, near the start of the ploughing season. This is the first of two population peaks (during ploughing and harvest season) when we expect more seasonal workers present in Namibia. The end-line cross-sectional survey will be conducted in April, towards the end of the peak harvest season and before migrant workers are expected to return to Zambia.

Sampling frame and sampling strategy

The sampling strategy will differ between Zambezi Region and Ohangwena Region for the baseline and endline surveys. This is necessary, as cattle posts used by cattle herders from Ohangwena Region are located in Angola.

Within each of the HFCAs selected for study inclusion in Zambezi Region, survey staff (including HEWs) will work with village headman to ensure that a full census list of farm/cattle post owners is available prior to the cross-sectional survey. Farms/cattle posts will be selected from the census lists via simple random sampling, with probability proportional to the projected HRP population size of the health catchment area. In this region, a second round of sampling may be conducted amongst social contacts nominated by the primary sample in order to evaluate key network characteristics.

Ohangwena Region has a complete sampling frame of cattle owners available from the Directorate of Veterinary Services in Namibia, which should be made accessible for this study. Based on preliminary data collected from cattle owners, we assume that each cattle owner employs an average of 3 cattle herders, of which 2 will be accessible during the survey. If successfully obtained, we will randomly select cattle post owners from the list to obtain a representative sample of cattle herders. The list will be oversampled by 20% in order to obtain the required sample size of eligible cattle herders who cross the border with Angola. Only cattle herders who meet eligibility criteria and are employed by selected cattle herders will be included in the cross-sectional survey. As the majority of cattle posts are located in Angola, all sampling activities will be closely coordinated with selected owners and recruitment will occur at water points and other venues to coincide with distribution of vector control packs. A similar sampling approach will be conducted in late May, when cattle herders are present in Namibia for grazing the cattle following harvest and the 'festive bull competition'.

If we are not allowed access to this sampling frame, we will use time-location sampling (TLS) to obtain a representative sample of eligible cattle herders in Ohangwena Region. In this situation, a sampling frame will be constructed based on a list of eligible venues identified during the formative assessment and venue-day-time (VDT) periods of 2- to 4- hours in duration will be defined for each venues. These eligible venues include water points and coka shops. The maximum number of sampling events staff are able to support and based on achieving the sample size over a 2 week period will determine the number of VDTs to be randomly selected from the sampling frame. These sampled VDTs will be scheduled, along with an alternate VDT. During sampling events, all potential participants will be enumerated, approached and eligibility determined. The representativeness of the sample will be optimized by targeted outreach through cattle owners to encourage all cattle herders to return during the survey time period. This process should be aided by scheduling the survey to coincide with distribution of vector control packs.

Study identifiers

All farm/cattle posts owners in both regions will be given a study ID card and assigned a unique study identifier, which will be used throughout the study to identify repeat visits to locations and track intervention distributions. This ID will also be linked to individuals presenting at health facilities through a line list based on the name, telephone number and/or village of residence of the owner.

Appendix S4 – Study protocol

<u>Questionnaires and human specimen collection</u>

In Zambezi Region, selected worksites will be visited and each worker interviewed by a study staff member using a tablet or paper form. To capture any workers not present at time of survey, study staff will plan for an overnight stay whenever feasible to schedule visits for early morning or late evening, but will have a maximum of four visits to each farm/cattle post. In Ohangwena Region, a booth will be set up at water points and other venues for a period of two weeks for the baseline survey, from which vector control pack distributions will also operate. Selected cattle post owners will be asked to inform herders of the survey in advance so that they can return to Namibia, who will be identified and interviewed by a study staff member at the booth using a tablet or paper form. At endline, the same approach will be used to recruit selected cattle herders at water points and other venues if possible (such as the Festive Bull Competition).

The survey questionnaire (Appendix 7) used for interviewing will be developed in English with input from local health staff. This will then be translated to Silozi and Oshikwanyama, and back-translated by a fluent bilingual health expert prior to field testing. The survey questionnaire will capture demographics, assess potential risk factors for malaria infection, and collect network data. Information collected will include age, gender, pregnancy, nationality, ethnicity, occupation, socioeconomic status, travel history, malaria knowledge, history of malaria, treatment seeking for fever in the past two weeks, individual use and adherence to preventive measures, housing structure type, cross-border and domestic travel, participation in each intervention, and frequency of outdoor sleeping or occupational activities at night. In addition, the use and condition of LLINs registered to each participant will be assessed visually to understand the durability of nets under various conditions. Egocentric network data will be collected in a subset of worksites and in the network sample in Zambezi Region to improve implementation effectiveness by identifying opinion leaders and to measure network characteristics. Three name generators will be used to identify social contacts in the study area who provide social or health support, or traveled with them to the worksite. After participants enlist supporting alters, they will be asked to provide information on alters' demographics, malaria risk behaviors, prior malaria diagnoses and information about social network factors (e.g., network size, relation types, and ego-alter contact frequency).

During the surveys, all eligible agricultural workers and cattle herders will be invited to participate in an RDT and blood collection component. Informed consent will be obtained from all participants, including parental or guardian consent for any participant younger than 18 years of age. After consenting, the study team will capture axillary temperature, and test each individual using a standard RDT ((CareStart™Malaria HRP2/pLDH Combo Test), followed by collection of four DBS on filter paper.

If found positive by RDT, treatment will be administered as in Table 3. Per national policy, a single low dose of primaquine (0.25mg/kg of weight) will be administered to study participants on the first day of treatment in addition to AL (Table 4). Contraindications to primaquine include pregnancy (any trimester), age less than 12 months, infants weighing less than 10 kilograms, women in the first 12 months of breastfeeding, and prior allergic reaction to primaquine. Participants with contraindications to primaquine will not be excluded from the study. Women of reproductive age will be assessed for treatment as in Section 9.1.3.

Table 4. Primaquine weight-based dosing

**Body weight**	**Tablets (dose)**
10-24 kg	1/2 tablet (3.75mg)
25-49 kg	1 tablet (7.5mg)
>50 kg	2 tablets (15mg)

Case and control survey data

A case-control study will be nested in evaluation activities and conducted throughout the study period (December 2019 to May 2020). This activity will be facilitated by a research assistant posted to each study health facility and include data collection for HRP cases (RDT-positive) identified by HEWs or at the health facility and a subset of RDT-negative HRP controls identified during RACD at HRP worksites. In addition, the research assistant will support health facility staff to ensure that key data for all incident malaria cases are completed on the RDT questionnaire and RDTs stored for future molecular testing.

<u>Case definition</u>

Patient will be classified as a case if he/she is confirmed positive for malaria by RDT or microscopy within study health catchment areas in Zambezi and Ohangwena regions. Cases may be identified by a HEW or at health facilities between December 2019 and May 2020. All participants must meet eligibility as a member of a target HRP group and will be recruited from patients attending the eight health facilities.

Inclusion criteria

Subjects must fulfill ALL of the following inclusion criteria to be eligible as a case in the study:

- Attending a selected health facility/district hospital or visiting the HEW, and
- Testing positive by RDT or microscopy, and
- State their main occupation as an agricultural workers (Zambezi only) or cattle herder, and
- Reports travel to Angola during the current malaria transmission season (Ohangwena only), and
- Working on a farm or cattle post within the study area, and
- Willing and available to participate in the study.

Exclusion criteria

- Testing negative by RDT or microscopy, or
- Diagnosed clinically, without any diagnostic test for confirmation, or
- Unwilling or unavailable to participate in the study.

<u>Control definition</u>

Age-and gender matched individuals will be randomly selected from the same neighborhood as the HRP index cases during RACD at worksites, and will be classified as a control if she/he is confirmed negative by RDT or microscopy. These neighborhood matched controls will be recruited in a 3:1 ratio to cases. Matching by neighborhood reduces the case ascertainment bias related to variations in geographic accessibility to the health clinic, which is a potential problem for community controls. This design allows greater statistical power to evaluate micro-epidemiological features related to individual risk factors.

Appendix S4 – Study protocol

Inclusion criteria

Subjects must fulfill ALL of the following inclusion criteria to be eligible as a control in the study:

- Reside in the neighborhood of an index case, and
- Same gender and age category as the index case, and
- Testing positive by RDT or microscopy, and
- State their main occupation as an agricultural workers (Zambezi only) or cattle herder, and
- Working on a farm within an intervention health catchment area, and
- Willing and available to participate in the study.

Exclusion criteria

Subjects must NOT meet any of the following criteria to be eligible as a control in the study:

- Testing positive by RDT or microscopy, or
- Diagnosed clinically, without any diagnostic test for confirmation, or
- Prior malaria diagnosis within the preceding month, or
- Taking malaria prophylaxis or treatment in the preceding 14 days
- Unwilling or unavailable to participate in the study.

Selection and interview of cases and controls

All malaria cases matching a recruitment profile and confirmed by RDT or microscopy who are diagnosed in intervention health facilities/district hospital or by HEWs will be included in the study. All cases who are diagnosed at the health facility or hospital will be interviewed by a research assistant on the same day.

In this study, the neighborhood of an HRP index case is defined as the worksite or a cluster of worksites with a shared water source. Neighborhood controls will be randomly selected from workers present at the farm/post and screened during RACD upon follow up of each eligible case within 7 days of detection. Individuals will be screened for inclusion, tested for malaria, and if negative by RDT/microscopy, fully enrolled by the field workers. Whenever possible, interviews will be scheduled in advance in coordination with the farm/cattle post owner and avoid conflict with working activities.

In Zambezi region, a second round of sampling may be conducted amongst social contacts nominated by index cases and controls in order to evaluate key network characteristics.

Survey Interview and blood samples

Cases will be interviewed by study staff at health facilities using a short questionnaire providing detailed information on suspected risk factors and controls/network contacts interviewed at the time of recruitment. Themes to be explored by the survey questionnaire (Appendix 8) will include:

- Socio-economic and demographic characteristics
- Farm/cattle post conditions
- Travel and mobility patterns
- Occupational risk activities
- History of malaria-related symptoms and infection
- Knowledge and attitudes regarding malaria
- Knowledge, attitudes and use of malaria prevention methods
- Participation in each intervention
- Social network characteristics

Egocentric network data will be collected in a subset of cases and controls in Zambezi Region to improve implementation effectiveness by identifying opinion leaders and to measure network characteristics. Three name generators will be used to identify social contacts in the study area who provide social or health support, or traveled with them to the worksite. After participants enlist supporting alters, they will be asked to provide information on alters' demographics, malaria risk behaviors, prior malaria diagnoses and information about social network factors (e.g., network size, relation types, and ego-alter contact frequency).

RDTs used for initial case-control classification and DBS will be collected and stored for analysis by more sensitive diagnostic methods, such as PCR, and potentially used for a sub-group analysis.

Confirmed malaria case incidence

The confirmed case incidence from all reporting units (including health facilities, district hospitals, and HEWs with RDTs) will be captured throughout the study with support from study or health center staff. In the months prior to the start of study activities, trainings will be conducted with HEWs and health facility staff to systematize the collection of farm/cattle post identifiers and HRP eligibility at health facilities for confirmed malaria cases. Routine supervision of health facility staff will be conducted by study staff to ensure accurate recording of location data in health facility registers throughout the trial.

In addition, study staff will complete a short questionnaire affixed to each RDT of cases that includes basic information included in the registry, eligibility as an HRP (occupation) and farm/cattle post owner name and telephone number and travel history.

Routine programmatic data

Routine programmatic data on community-level interventions for the year prior to study baseline and throughout the study will be collated at the health facility level in order to control for potential sources of variation between HFCAs. This will include, for each intervention type:

- LLIN: ANC distributions (number), case investigation data (% own), RACD (% own)
- IRS: Routine spray campaign coverage
- RACD: Index case coverage, population coverage

10.2.4 Data management and analysis

Data will be collected via ODK-based tablet application with internal range checks or paper-based with subsequent double-entry, and will be stored in Microsoft Excel or Access.

<u>Outcome 1: Effective coverage of malaria interventions</u>

Coverage data from cross-sectional surveys will be analyzed in a binomial regression model with random intercepts at the health facility catchment level. The model will include a fixed effect for each study arm and a fixed effect for time period (pre- and post-intervention). The interaction between these two terms will be the primary effect measure (a difference-in-difference estimator). Subgroup analyses will be conducted by region.

<u>Outcome 2: PCR-based parasite prevalence</u>

The prevalence of infection via PCR will be assessed at two time points during the cross-sectional surveys and analyzed as intention-to-treat, regardless of intervention uptake. First, a difference-in-differences analytic approach will use generalized linear models with fixed effects to allow for clustering

within health facility catchments. The binomial distribution will be used to analyze prevalence outcomes (logistic regression) and include a fixed effect for each study arm and a fixed effect for time period (pre- and post-intervention). The interaction between these two terms will be the primary effect measure (a difference-in-difference estimator). Analyses will be adjusted for age, sex, health facility catchment, and other potential confounders. Subgroup analyses will be conducted by region.

Second, the protective effects of the intervention on malaria infection at endline will be estimated for individuals using multiple regression and adjusting for clustering at the health facility level. The protective effect of the intervention is expressed by the regression coefficient for exposure, i.e. the adjusted risk ratio of having a positive outcome in the intervention group versus in the control group.

<u>Outcome 3: Odds of symptomatic malaria</u>
Multilevel conditional logistic regression analyses will be conducted to estimate the odds of exposure to the intervention in cases versus controls, controlling for potential confounders at different scales, including those at the individual level (e.g. behavioral and socio-demographic), sleeping structure level (e.g. IRS, housing structure), farm/cattle post level (e.g. rainfall, vegetation, elevation) and site level (intervention versus control). A fixed effect for health facility will be used to adjust for additional clustering at this level. Subgroup analyses will be conducted by region, and explore the impact of excluding PCR positive controls.

<u>Outcome 4: Malaria seroprevalence in HRPs</u>
Seroprevalence of infection via ELISA will be analyzed as intention-to-treat, regardless of intervention uptake, and use generalized linear models with fixed intercepts to adjust for clustering within health facility catchments. The binomial distribution will be used to analyze prevalence outcomes (logistic regression) and include a fixed effect for each study arm and a fixed effect for time period (pre- and post-intervention). The interaction between these two terms will be the primary effect measure (a difference-in-difference estimator). Analyses will be adjusted for age, sex, health facility catchment, and other potential confounders. Subgroup analyses will be conducted by region.

<u>Outcome 5: Outpatient malaria case incidence by health facility, stratified by HRP status</u>
Monthly counts of confirmed malaria cases from the health facility registers will be stratified by HRP status and analyzed in a time series Poisson or negative binomial regression model with fixed effects at the health facility catchment level. The models will include a fixed effect for each study arm and a fixed effect for time period (pre- and post-intervention). The interaction between these two terms will be the primary effect measure (also known as the difference-in-differences estimator). Pre/post-intervention will be determined as all time periods before the start date of the intervention in the areas considered as being pre-intervention and all time periods after (and including the start date of the intervention in the area considered as being post-treatment).

10.3 Assessment of feasibility, acceptability of and adherence to interventions

The feasibility and acceptability evaluations will be conducted using a mix of quantitative data collection through interviews and the endline cross-sectional surveys, and qualitative data collection through key informant (KI) interviews and focus group discussions (FGDs). Participants of these assessments will include HRPs, farm and cattle post employers or owners, HEWs, and other health sector staff. Adherence to AL and compliance to VC interventions will be assessed through a pill count survey, LLIN and repellent

use tracking logs and the endline cross-sectional survey. In addition, LLIN condition and weight of topical repellent containers will be assessed at 30 days and at the endline survey.

10.3.1 Outcome measures

Outcome 1: Operational coverage of targeted malaria interventions

The operational coverage will be estimated at the individual, structure and/or farm level as the percent of the population that were approached by the intervention teams to offer each interventions, among those eligible for inclusion. This information will be obtained from a combination of intervention data and enumeration data. Additionally, the proportion of individuals, structure and/or farm accepting each intervention, among those eligible for inclusion in the intervention, will be estimated, providing an estimate of the effect coverage of each program at the time of implementation. Data for the denominator of structures and farms targeted for IRS will be ascertained from enumeration activities. To the extent possible, individual and farm/cattle post level factors associated with coverage will be assessed using mixed effects logistic regression.

Outcome 2: Feasibility and cost of targeted delivery of interventions to HRPs

The operational feasibility of HEWs to conduct enhanced screening, presumptive treatment and support distribution of vector control interventions will be estimated at the individual level as the percent of HEWs and farm/cattle post employers who rate the intervention activities as very easy, somewhat easy, somewhat difficult, and very difficult. Qualitative surveys will be conducted with HEWs, other health sector staff, and farm/cattle post employers. The costs and cost-effectiveness of the intervention package will be assessed as an incremental cost effectiveness ratio (ICER), as well as cost per population and case averted as measured through the difference in infections identified subsequent to the intervention. Program data on costs/expenditure for each intervention will be collected and combined with estimates of program effectiveness to estimate these measures.

Outcome 3: Acceptability of targeted delivery of interventions to HRPs

The acceptability of a targeted delivery of presumptive treatment and vector control interventions to HRPs will be assessed as the proportion of targeted individuals refusing each intervention, among those eligible for inclusion. Acceptability of IRS will be assessed as the proportion of targeted farms refusing LLIN, among those with at least one sprayable structure missed during the spray campaign. Qualitative data collection methods such as focus groups and key informant interviews with HRPs, HEWs, employers and other health sector staff will be implemented.

Outcome 4: Assess treatment adherence and intervention compliance

Participant adherence to presumptive treatment will be assessed within a sub-sample of people distributed the drug and measured as the proportion of people who complete all pills in the pill pack after receiving the first dose of AL by DOT. Changes in proportions over time will be quantified between the two distribution rounds.

Self-reported user compliance to LLINs and topical repellents will be assessed through the quantiative baseline and endline cross-sectional surveys, as well as through a use tracking log tracking compliance over a 30-day period (Appendix 16 and 17) in the cohort included in the pill count. LLIN use and condition will be empirically assessed during the endline cross-sectional survey and at 30 days, as well as repellent containers weighed after 30 in the cohort. Qualitative information on use of these interventions will be collected during key informant interviews and focus group discussions.

10.3.2 Sample size

Pill count

A previous study in Zambezi Region presumptively treating individuals living in households surrounding index cases found that adherence was 100% among individuals in follow-up pill counts who still had their blister packs. Among individuals without their blister pack, all but one (1/315) reported adherence. We expect adherence to be lower amongst HRPs, given that they may perceive their risk to be lower than those living in close proximity to an index case. There are also more migrants in this group, who have fewer ties to the local community and may feel less motivated to comply with public health measures supported by traditional authorities.

The sample size for the pill count survey was calculated as 320, which will provide an absolute precision of ± 5%, assuming the true medication adherence is 80% of people taking all six doses of the Al regimen and allowing for a 20% non-response rate. The sample will be selected from individuals presenting for AL distributions in each health catchment area, with probability proportional the size of the expected HRP population in each catchment (based on farm/cattle post census data). If possible, individuals will be randomly selected from employee lists provided at each farm or else recruited using a sampling fraction based on the estimated number of eligible workers.

The same cohort will be used to assess self-reported use of LLINs and topical repellents over a 30 day period. Assuming people report compliance as 60% and 40% of person days, the above sample size will provide an absolute precision of ± 6% (allowing for 20% non-response rate).

Qualitative assessment

As this is a qualitative formative assessment, there is no formal power calculation for sample size. Estimates presented below are based on prior experience with qualitative work in these populations.

The study will aim to conduct 2-4 focus groups with HRPs in each intervention HFCA and 8-12 in-depth interviews in each intervention HCA with (i) agricultural workers, (ii) cattle herders, (iii) Health Extension Workers and health facility-based staff involved in malaria diagnosis and treatment, and (iv) farm and cattle post owners. Focus groups will comprise 6-10 participants each.

10.3.3 Data collection

Intervention implementation data

Data collection during presumptive treatment and vector control interventions are described below.

<u>Presumptive treatment</u>

For all agricultural workers and cattle herders treated in intervention areas, after obtaining informed consent, a line listing of eligible workers will be completed for each location where the distribution is held (i.e. water point etc). HEWs will record information (Appendix 1) on the following for each eligible individual:

- Name and ID of farm/cattle post
- Date employment started at location
- Participation in previous AL distribution *[T2 only]*
- Age and gender
- Nationality

- Occupation
- ITN usage
- Travel history
- Recent care-seeking behavior for fever
- Unique identifier (for population size estimation)

If feasible, DBS will be collected for all consenting participants who are provided with presumptive treatment in Ohangwena Region and at the second distribution point in Zambezi Region. This information will help to quantify changes in the parasite burden during different seasons.

Sites will be geo-located and HEWs may also obtain contact information from consenting persons to allow for follow-up on positive cases and potential creation of support networks for other HRPs.

Targeted IRS mop up

Spray teams will coordinate closely with farm/cattle post owners to identify and geo-tag all sleeping structures at a farm/cattle post. Owners will be asked a short series of questions for each structure, in order to determine existing spray coverage, identify eligible structures for the mop-up and record information to track spray status:

- Name and ID of farm/cattle post
- Unique structure ID
- Type of structure
- Month it was last sprayed
- Number of occupants
- Mop-up spray status

LLIN and vector control pack

LLIN and vector control packs will be distributed to farm owners at the time of the IRS mop up in Zambezi Region, based on the results from the farm/cattle post census. In Ohangwena Region, vector control interventions will be distributed at the time of the baseline cross-sectional survey. LLINs and topical repellents will be marked with a unique identification code linked to each farm/cattle post and provided for any worker who sleeps at the worksite. Owners will be required to maintain a line listing of LLINs and vector control packs distributed to each employee (Appendix 15), which will include information on:

- Name and ID of farm/cattle post
- LLIN ID
- Vector control pack ID
- Date checked out/in
- Location hung (permanent structure, open, mobile)
- Employee details: date employment, name, unique identifier (for population size estimation)
- Containers of repellent provided

Workers will be required to return the LLIN to the owner when they complete employment at the farm/cattle post. HEWs will conduct a stock check occasionally and during the second AL distribution to see if LLINs are hung correctly, in use and still at the farm/cattle post where they were distributed.

Pill count

At each distribution time point, HEWs will conduct a pill count in a sub-sample of HRPs who received the first dose of AL by DOT. HEWs will collect key demographic information and locations for each participant recruited into the sample during the drug distribution. HEWs will arrange a follow up visit with participants between Days 4 and 10 for a pill count. At this visit, HEWs will count any remaining pills in the blister packs and complete a short tablet questionnaire by interview (Appendix 3). The questionnaire will collect information on:

- Demographics
- Location
- Number of pills remaining and which days were incomplete
- Reasons why treatment not completed

Repellent and bed net log
The same cohort of HRPs selected for the pill count (above) will be asked to complete a daily log tracking frequency of use of topical repellents and bed nets (if received) over a 30 day period (Appendix 16 and 17). LLIN condition and repellent containers will be respectively assessed through visual observation and weighing at the end of 30 days to correlate with reported use.

Cross-sectional surveys
During endline surveys described in 10.2.3, data will also be collected on past participation in AL distributions, receipt of medication and interventions, acceptability, adherence to medication and compliance to LLINs and topical repellents. Potential factors related to non-adherence and non-compliance will be explored from these data. Empirical observation of LLINs (use and condition) will be assessed at endline.

Qualitative assessment
At endline, FGDs and KIIs will be conducted with high-risk populations (HRPs), HEWs, employers and other health facility staff to qualitatively assess perceptions around targeted delivery of presumptive treatment and enhanced vector control.

<u>Study populations</u>
Separate FGDs and KIIs will be held for:

1) High risk populations (HRPs), including representation by sub-groups:
    a. Migrant agricultural workers [Zambezi only]
    b. Agricultural workers who work outside at night [Zambezi only]
    c. Cattle herders
2) Health Extension Workers and health facility-based staff involved in malaria diagnosis and treatment
3) Farm and cattle post owners

<u>Sample selection</u>
HRPs selected for inclusion in FGDs and KIIs will be identified during the endline cross-sectional survey based on specific risk criteria outlined above. An effort will be made to segment focus group populations by gender in order to ensure representation of both males and females. Focus groups may be further subdivided by age and gender based on the social and power structures within the communities, as deemed necessary by local collaborators.

Health extension workers will be recruited through the health facility that they are associated with and a convenience sample of farm/cattle post owners selected from intervention implementation records.

Efforts will be made to select a farm/cattle post owners representing worksites of different remoteness, size and crop type.

<u>Content of focus group discussions and in-depth interviews</u>
KIIs and FGDs will focus on assessing the operational feasibility, acceptability and compliance with targeted delivery of presumptive treatment and enhanced vector control to HRPs. Themes to be explored during the KIIs and FGDs include the following:

- Perceived effectiveness of interventions
- Operational feasibility, attitudes and practices related to HEW screening, presumptive treatment, and IRS, LLIN and mosquito repellent use
- Individual and group factors enabling uptake of interventions
- Perceived facilitating factors and challenges to scale-up of the program

The focus group guides and topic guides for In-depth Interviews with key informants are provided in appendices 10-14.

Focus groups will be facilitated by 2 members of the research staff: 1 moderator and 1 note-taker. A locally-appropriate location will be used to hold the focus group discussions (ie, community center, health facility, etc). After a brief introduction, the moderator will obtain informed consent separately from each participant. All sessions will be audio recorded. During the sessions, the research staff will generate notes as the discussion unfolds to help formulate follow-up questions and probes, with no identifying information.

Key informant interviews will also be facilitated by two members of the research staff (moderator and note-taker). An appropriate, private location will be used within the community or health clinic for the KII. The moderator will obtain informed consent, and will audio-record the session. Research staff will generate notes during the session to help generate probes and follow up questions. No identifying information will be recorded.

Costing
Detailed expenditure data on the costs of delivery of each intervention. Total costs as a unit as well as cost-effectiveness will be assessed as an incremental cost effectiveness ratio (ICER), as well as cost per population and case averted as measured through the difference in infections subsequent to the intervention.

Calculations will include costs for all consumables, as well as staff time. The types and potential sources of expenditure data is shown in Table 5.

Table 5. Types and potential sources of expenditures

Expenditure category	Types of expenditure needed	Potential sources of information
Personnel	All human resource expenditures and time contributions<br>• Salary/wage payments<br>• Value of benefits<br>• Volunteer labor	• Study expenditure for salaries and benefits<br>• Work logs or reports from health sector staff
Commodities and Services	All supplies and services used toward intervention activities<br>• In-kind donations<br>• Purchased commodities (including acquisition costs)<br>• Utilities<br>• Travel/transit costs: fuel, transit fees and services, airfare<br>• Reproduction costs, postage<br>• Staff trainings: per diem payments, trainer fees, consultants<br>• Current rental value of property	• Printing and postage receipts<br>• Training budgets and receipts<br>• Vehicle travel logs, fuel receipts<br>• Utility bills<br>• Consultant invoices

10.3.4 Data management and analysis

Individual level intervention data for presumptive treatment and vector control distributions will be collected using paper-based forms and/or ODK and data aggregated at the farm and health catchment level on a daily basis. This information will be used to monitor coverage estimates and adapt distribution strategies if necessary. Data collection tools for IRS mop up will be selected so that databases can be easily integrated with MoHSS routine IRS data.

Data from pill counts and cross-sectional surveys will be collected via ODK-based tablet application with internal range checks or paper-based with subsequent double-entry, and will be stored in Microsoft Excel or Access. Repellent and net use logs will be paper based and entered into a standardized database. Factors associated with not completing the full AL course and non-compliance with vector control interventions will be assessed in the endline cross-sectional and qualitative studies, which will provide estimates for the proportion of HRPs who received the interventions at each distribution, self-reported treatment/intervention adherence, and information on many socio-demographic and behavioral factors. Costing data will be entered and managed in a pre-formatted Microsoft Excel template.

For key informant interviews and focus groups, the note taker and moderator will discuss interview notes/similarities, and responses will be recorded/finalized at the end of each interview. The moderator for each group will transcribe the recording and once all interviews and focus groups have been conducted, data transcribed, and analysis completed with results disseminated, all of the audio recordings

will be destroyed. An experienced translator will translate the transcript to English. The local study coordinator will supervise and review the local language and English transcripts to ensure accuracy. The focus group discussions and key informant interviews will take approximately 1-2 hours each and occur at an appropriate time for the study respondents (ie, weekday vs weekend; morning vs afternoon).

Data from the interviews and focus groups will be entered into Dedoose or a similar program for simple thematic analysis and stored on a password protected device. All data will be stored on a password-protected database and will only be available to the study personnel. All data from participants will be coded using a study identification number in place of the individual's name. Data will be analyzed to provide preliminary descriptive statistics on the study population and recurrent themes within the assessment. Data will only be used for the purposes of this study.

All participants will receive compensation for travel costs associated with participating in the focus group or interview. Light snacks and non-alcoholic refreshments will be provided during the focus group and interview sessions.

10.4 Surveillance capacity building and assessment of targeted reactive case detection

10.4.1 Outcome measure

<u>Outcome 1: Test positivity rates from RACD, stratified by HRP status and location (village or worksite)</u>
Test positivity rates will be calculated as the proportion of individuals screened during routine RACD who test positive for malaria by RDT and PCR. Rates will be stratified by HRP status and location of event (either within villages or at an HRP worksite).

10.4.2 Sample size

The minimum detectable difference in RDT test positivity between non-HRP RACD at villages and HRP RACD at worksites was 2%, assuming a test positivity of 2% at villages, sample sizes of 3300 and 900 in each group and a design effect of 2.

Justification for the sample size assumptions are outlined below:

- <u>165 non-HRP and 180 HRP index cases with RACD</u> within the study area in Zambezi Region: based on case incidence of 10 per 1000 people and 30 per 1000 people in respective non-HRP and HRP populations and 50% of non-HRP cases followed up by the program.
- 20 individuals are screened on average in each event in a village and 5 individuals in each event at a farm/cattle post

10.4.3 Data collection

According to MoHSS guidelines, RACD is carried out in all individuals who reside in households located within 500m of an index case household following a case burden prioritization. RACD activities are

conducted at the village or worksite for HRP, however RACD coverage in HRP at worksite is low due to remoteness and lack of accessibility of these areas.

In addition, the study team will support implementation of RACD at worksites for eligible HRP index cases (i.e. farms and cattle posts) using the MOHSS RACD data collection system. Data collected will include the following (Appendix 4):

- The index case ID
- Name/ID of RACD location
- Demographics of individuals tested
- RDT results

Coverage at these locations is frequently very low, due to limited program capacity and geographical remoteness. Eligible HRP index cases will be visited at their worksite by a team including the HEW, a field investigator and the research assistant based at the health facility.

RDTs will be saved from screening activities at HRP worksites, for later molecular analyses.

10.4.4 Data management and analysis

Existing RACD data collection systems developed by the MoHSS are tablet based and collect information on the index case and all individuals screened during RACD. Unfortunately, paper-based data collection is typically used in place of tablet-based systems due to technical difficulties operating the tablets and software. The MEI will support technical capacity building and provide assistance to ensure that tablet-based data collection systems are used for all RACD activities within the study area. The same platform will be used for all RACD data collection.

Test positivity rates will be compared between RACD conducted at HRP worksites and non-HRP villages.

10.5 Population size estimation and turnover assessment

10.5.1 Outcome measure

<u>Outcome 1: Population size estimate and turnover between seasons</u>

The population size of target HRPs will be estimated in intervention areas using a combination of multiplier and multiple capture-recapture (CR) methods. In Ohangwena Region, the population is assumed fixed, although accessibility of cattle herders in Namibia may vary throughout the year. In Zambezi Region, the population is estimated at two distinct time periods in order to capture turnover between agricultural seasons.

Changes in the number of HRP present over time might be due to movement into and out of the study area (mobility) and individuals initiating risk activities or ceasing to practice risk activities (turnover). Turnover could result from younger individuals initiating the risk activity (aging in), older individuals aging out, temporary interruptions in practicing the risk activities, and more lasting occupational changes. Each of these elements of turnover might fluctuate over the course of the year due to seasons and other patterns. Turnover will be estimated by comparing captures between periods and based on data from the

cross-sectional surveys. This may help authorities plan how many new individuals will need to be provided prevention information, LLINs, treatment, etc. after the harvest season.

10.5.2 Data collection

Multiplier method activities

Multiplier methods use two sources of data to estimate population size: (1) a count of unique individuals from the target population receiving a service and (2) a representative estimate of the proportion of the target population in receipt of the service or object.

In Ohangwena Region, the target population (cattle herders who graze cattle in Angola) is assumed to be relatively stable, despite high levels of cross-border movement throughout the year which affects accessibility on the Namibian side of the border. The cattle immunization clinic scheduled for July provides an opportunity to obtain a count of eligible cattle herders when many are in the country and the cross-sectional surveys provide a representative sample of the entire target population.

In order to ensure key assumptions of the multiplier methods:

- Data collection activities for the two data sources will be conducted independently (i.e., so that being present in one capture does not correlate with being present in another). The first data source (clinic) needs not be random and the second source (cross-sectional survey) will be random.
- Eligibility screening will be conducted during the clinic to ensure only target HRPs are counted at the clinic and included in the cross-sectional survey.
- Data sources will be conducted in the same health catchment areas and assume no significant in/out migration of the study population (ie have aligned time periods).

No individual data need to be recorded during the initial count at the cattle immunization clinic.

Capture recapture activities

Although eligible cattle herders in Ohangwena region are considered a more stable population, it is necessary to consider two distinct time periods in Zambezi Region for population size estimation:

1) During a part of the ploughing season, reflecting the total HRP present at any time from Nov 2018 to January 2019 (Period 1)
2) During a part of the harvest season, reflecting the total HRP present at any time from Feb 2019 to May 2019 endline survey Period 2

For each period, multiple CR will combine person-level data from ≥ 3 independent data sources (i.e. mapping, survey, AL distribution, vector pack distribution) to estimate the total number of HRPs. Potential capture recapture data sources are listed in table 6. Each data source serves as a “capture” of the target HRP population. The captures may overlap, including some of the same individuals. There is no requirement that any capture is a random sample. To satisfy theoretical assumptions of CR and ensure adequate precision of the size estimates:

- Data collection activities used as captures will be conducted independently (i.e., so that being present in one capture does not correlate with being present in another).
- Eligibility screening will be conducted during each capture to ensure only target HRPs are met.
- Captures will record a unique identifier to link individuals across captures for analysis.
- Captures will aim to reach ≥ 200 HRPs each.

Additional activities may be conducted to ensure a sufficient number of captures meeting the above criteria if it is not possible to conduct mapping, AL distribution, and vector distribution independently or if their reach is insufficient. For example:

- Outreach to provide education on malaria prevention and/or testing services
- A special meeting or event specifically for HRPs
- Distribution of a unique object to HRPs

Table 6. Potential capture-recapture data sources

**Period 1 captures (Nov 2018 - Jan2019)**	**Period 2 captures (Feb 2019 to May 2019)**
Mapping and enumeration	Mapping and enumeration*
Baseline survey	Endline survey
AL distribution (round 1)	AL distribution (round 2)
Vector control pack distribution	
Educational outreach (round 1)	Educational outreach (round 2)
* Ohangwena only	

Data sources

<u>Mapping and enumeration</u>

Preliminary meetings have been held to collect data from farm and cattle post owners through meetings organized through headman and local councilors. These meetings were intended to collect data from all owners on the number of HRPs working at each location, how this population varies throughout the year and the number and type of sleeping structures. However, attendance at these meetings varied widely and data received is incomplete.

Based on estimated numbers of cattle post owners per village and extrapolation of data from forms completed so far by owners, there is wide variation in the distribution of cattle posts and farms in the two regions. We will conduct more comprehensive mapping and enumeration in intervention areas prior to and during IRS mop-up in December 2019 (ploughing season in Zambezi Region). A census listing of cattle owners with contact details in Ohangwena region will facilitate this process. Data collected will include:

- Name and ID of farm/cattle post
- Nearest village, constituency, health district
- Name of farm/post owner
- Monthly number of workers by nationality and gender
- Number of workers present by nationality and gender
- GPS location

The unique ID code of workers present during the mapping, and who meet eligibility as HRPs, will be recorded so that the mapping can serve as a capture.

Baseline and endline surveys

The cross-sectional baseline and endline surveys will each serve as an independent capture by collecting the unique ID code of survey participants to link to other captures. Mobility modules will gather data on the timing and duration of completed and planned travel into and out of the respective study area.

Intervention implementation data

Data collection during presumptive treatment and vector control interventions are described in the above section. Each source described will provide count data for a separate capture or service access, and will be linked through a unique code.

- Presumptive treatment with AL in December 2019 and/or March 2020
- LLIN distribution while in Namibia

Efforts will be made to carry out presumptive treatment, LLIN distribution, and vector pack distribution independently so that they may serve as independent captures.

Educational outreach

Educational components of the intervention will be carried out together with other interventions. However, additional activities may be planned to increase the number of captures if necessary, such as an educational booth at watering holes in Ohangwena region**.** These activities would be facilitated by health extension workers and the study team, who would provide information about malaria prevention tailored to the target population and obtain count data for service access (linked through the same unique code).

10.5.3 Data management and analysis

Unique identifier

A unique identifier appropriate to the HRP population will be developed and will underlie the success of the size estimates. The unique identifier will be developed and evaluated prior to initiating the baseline survey, mapping and interventions (AL distribution, vector pack distribution, educational outreach).

To develop the identifier, meetings will be held with local study partners and key informants knowledgeable of HRPs. Elements appropriate to the context will be explored and may include:

- subject's initials
- a parent's initials
- sex/gender
- date of birth
- birth order
- birthplace or place of residence
- year or age when first engaged in the risk activity

Evaluation of the identifier will aim to ensure the code is reasonably unique (not likely to be the same for different people), reliable (likely to be reported the same over time), acceptable to the population, and feasible to implement rapidly in the field. A data capture form will be developed. The code will be evaluated on ≥ 50 individuals per region.

Data management
Data from cross-sectional surveys and intervention implementation will be managed as described in previous sections. Physical mapping and enumeration of populations will use a standard ODK-based tablet application with internal range checks or paper-based with subsequent double-entry, and will be stored in Microsoft Excel or Access.

Data analysis
The multiplier and capture-recapture data will estimate the total HRP present over the course of the study (November 2018 to May 2019) combining data from all captures and separately for the defined time periods. The number of HRPs present over the course of the year will also be estimated from the survey module on cross-border travel in the baseline and endline surveys. The R package 'SizeEstimation' and/or 'RCapture' will be used to generate population size estimates within a Bayesian Hierarchical framework that includes all sources.

Turnover will be estimated by the number of HRPs present during the harvest season who are not present during the ploughing season, (ie by comparing the Period 1 and 2 captures).

10.6 Vector assessment in agricultural areas of Zambezi Region

10.6.1 Outcome measures

<u>Outcome 1: Vector biting time and location, and indoor resting density in agricultural areas during cross-sectional surveys</u>
Vector occurrence and densities will be recorded through human landing catches (HLCs) and pyrethrum spray catches (PSCs). Vector species-specific biting time and location (indoors vs. outdoors), and human biting rates, will be evaluated indoors and outdoors in intervention and non-intervention areas during the cross-sectional surveys using HLCs. Human behavior observations (HBOs) will be conducted alongside HLCs to characterize exposure of humans to mosquito bites (i.e., human biting risk). In addition, PSCs will be used to measure indoor vector resting density. These data will be used to look at how vector species composition and relative abundances vary at baseline and between agricultural seasons, the potential effect of IRS, LLINs and spatial repellent interventions, and contextualize cross-sectional data on human risk behavior.

<u>Outcome 2: Insecticide susceptibility status and frequency</u>
Insecticide susceptibility bioassays will be used to assess vector susceptibility profiles over the two main agricultural seasons, in order to contextualize the likely impact of targeted IRS on transmission in HRPs compared to other interventions in the package.

10.6.2 Data collection

Data collection for a number of vector indicators will be carried out using standard methods in both intervention and control areas in Zambezi Region (Table 7).

Table 7. Indicators that will be measured through the entomological collections.

**Vector indicators (species specific)**	**Method**	**Targetted IRS + control**	**LLINs + repellant + control**
Vector occurrence	HLC/PSC	✓	✓
Vector density (unit time: hourly)	HLC/PSC	✓	✓
Vector biting behavior (location)	HLC	✓	✓
Vector biting behavior (time)	HLC	✓	✓
Human biting rates	HLC		
Insecticide susceptibility status	WHO/CDC	✓	✓
Insecticide susceptibility frequency	WHO/CDC	✓	✓
Indoor resting density	PSC	✓	✓
Human behaviors/biting risk	Human behavior observations (HBOs)	✗	✓

Site selection

The study will be restricted to six health catchment areas in Zambezi Region, given that we hypothesize that most cattle herders in Ohangwena are infected in Angola. There will be 3 HCAs in the control arm, and 3 HCAs in the intervention arm. One farm, or a cluster of farms, (composed of multiple sleeping structures) will be selected per HCA agricultural area, adding up to a total of 6 ‘farms’ (i.e., ‘sites’). Each intervention farm will be matched to a corresponding control farm, matched following the cross-sectional baseline survey by type of structures (with at least one part of sites with each type of structure), ecology, size of farm/cluster, population size and malaria prevalence by RDT.

Larval collections and morphological identification

Larval collections will be collected from a minimum of 3-5 of different breeding sites in each cluster of farms, until sufficient larvae are collected to use for the IR tests. This strategy avoids sampling individuals from single egg batches, which could result in a high proportion of siblings in the test population. Larval collections will take place during 7 days (or till enough larvae are captured for the required number WHO tube tests).
Samples from the same cluster and the same type of breeding site will be pooled before testing in order to provide a sufficient number of test mosquitoes. Larvae will be reared to the adult stage for insecticide resistance testing.

Appendix S4 – Study protocol

Information will be recorded for each sample location, including:

- Health catchment area
- Type of breeding site (e.g. rice field, rainwater collection, irrigation channel or well)
- Location of site (e.g. farm or cattle post)
- Global positioning system (GPS) coordinates from which the larval collection was made

<u>Adult mosquito collection and morphologic identification</u>

Adult *Anopheles* mosquito collection via HLCs and PSCs will be conducted at all sampling sites (farms) at baseline (December/January) and endline (March) in Zambezi Region. Only *Anopheles* will be collected; all other genera will be counted and then are purposefully discarded.

Available capacity will likely allow 2 field teams; thus, collections will occur sequentially, whereby one intervention site and its corresponding control site will be sampled at a time (about a week).

In each of the six sites, human landing captures (HLCs) (Appendix 18) will be conducted (indoors and outdoors) in one to two sleeping structures of each type of structure (e.g. traditional, tent, open structure with tarp, etc) over 4 (min) to 7 consecutive nights. Each collection will be carried out from 18:00 to 06:00 hours. Mosquitoes will be captured indoors and outdoors using two collectors per night, who will be rotated every 4 hours though the night, to avoid biases due to differences in attractiveness and ability in catching mosquitoes. Structures included for HLCs will be selected using the same approach across all sites. Selected HLC structures will be separated from each other by at least one other infrastructure (i.e., HLC structures should not be next to each other).

An additional collector will be positioned in any outside sleeping area, where people are not sleeping under any structure.

In addition, indoor resting density will be measured using pyrethrum spray catches (PSCs) (Appendix 20) in different structure types, targeting a sample of 4 structures of each type. PSCs will be conducted during the early hours of the morning. For structures with multiple rooms, PSCs may be carried out in the entire structure; however, at minimum, the PSC should target the sleeping room. Structures will not be repeated to ensure that residual insecticide does not affect catches. Selected PSC structures will be separate from HLC structures, and far enough from the HLC structures to avoid contaminating HLC structures with pyrethroid fumes.

All mosquitoes collected will be placed into screened collection cups and transferred to the Zambezi Region insectary (referred to as the 'Katima Insectary' or the 'KM Insectary') for morphological identification of species and species complexes, using standard keys used in Namibia [19]. Mosquitoes will be captured individually in vials which will be plugged with cotton wool and labelled by hour and collection site.

A representative subset of morphologically identification specimen of each species will have their species identification confirmed be validated by PCR (for *An. gambiae* and *An. funestus* complex mosquitoes). Non-amplifiers, and other species will have their species identity confirmed by sequencing of the ITS2 region.

<u>Human behavior observations</u>

During the HLCs, data collectors will record human behavior observations (Appendix 19) both inside and outside, at the end of each collection hour. Indicators recorded include:

- The number of people <u>awake</u>,
- The number of people <u>asleep under net</u>,
- The number of people <u>asleep without net</u>

These data will be compared with information from the cross-sectional survey and interpreted alongside results from the vector assessments to enable a determination of location based risk of being bitten (and any intervention related effects).

10.6.3 Data management and analysis

<u>Outcome 1: Indoor and outdoor vector resting and biting behavior in agricultural areas during peak population times</u>
Data will be entered into a standardized entomological database. Multivariate analysis (Poisson or Negative Binomial Regression Models) will be used to compare adult mosquito captures rates across collecting methods (HLC and PSCs), location (indoor/outdoor), and health catchment area. The number of adult Anopheles females caught in each collection method on each night will be treated as the outcome variable, and number fed will also be recorded for PSCs. Resting behaviors will be used to inform on IRS while biting behaviors will be used to inform on location based risk of being bitten, LLIN potential effect and Repellant based effect.

Descriptive analyses of mosquito population composition will consist of species composition and density by collection method and location. Seasonal abundance in the two periods will be determined using the ratio of total number of all mosquitos of each species collected during each assessment and the total number of all mosquitos collected during each assessment. All analyses will be in R software or STATA.

Molecular identification using PCR (for morphologically identified *An. gambiae* s.l. and *An. funestus* s.l.) will be using to determine specific species behaviors and insecticide resistance rates. Other Anopheles mosquitoes may be sequenced at the ITS2 or CO1 regions for species identification if required.

<u>Outcome 2: Insecticide susceptibility status</u>
Larvae collected in the field will be placed in glass vials filled with water, and stored in coolers with moist towels to avoid overheating of vials during transport of specimens from the field to the lab. Transport of live specimens will abide to the transportation SOP implemented by the Katima Insectary. In particular, during transport, air will be replenished in the vials by the opening and closing of vials every few hours, and temperature and humidity will be controlled and monitored throughout the drive to the insectary.

Larvae will be reared in the Katima insectary in accordance to the insectary's wild type mosquito rearing SOP. Larval instars 1-2 and instars 3-4 will be reared in separate larval trays, and will be fed (in addition to the organic matter collected along with the larvae) ground up brewer's yeast and dog biscuits. Adults will be feds 10% sucrose water. *Note that no breeding of adults will occur, hence, no blood-feeding is necessary.*

For the WHO insecticide susceptibility tests, we will aim to raise 120–150 adult female mosquitoes of each given species for each of the two insecticides tested; of these, 100 will be exposed to the insecticide that is being tested (in four or five replicates each of around 20–25 mosquitoes). The remaining 50 mosquitoes will serve as wild type "controls" (i.e. two replicates each of around 20–25 mosquitoes).

Adult female mosquitos derived from larval collections will be used in the standard WHO insecticide susceptibility bioassay or US Centers for Disease Control and Prevention (CDC) bottle bioassay to assess vector phenotypic resistance to insecticides commonly used for IRS (DDT and deltramethrin) [20]. This assay exposes mosquitos to known concentrations of an insecticide for a fixed period of time and records number of fatalities 24 hours after exposure. A total of 120–150 female mosquitoes should be tested for each insecticide at the discriminating concentration, with at least four replicates of 20–25 mosquitoes per test. Tests should be carried out at 25 °C ± 2 °C and 80% ± 10% relative humidity, at existing laboratories in Zambezi Region.

In the event of insufficient mosquito numbers, both males and females may be used for IR testing. If there are insufficient males and females for a full test (testing 4 replicates and 2 controls simultaneously), then testing of single or two replicates per day till obtaining all 4 replicates, will be acceptable. However, note that any results from such procedures will have to be subsequently confirmed via a full test according to the WHO protocol.

A standard form will be used for recording and reporting the results of bioassays, both mortality and knockdown rates. The assessment of mortality (i.e. a count of the number of dead mosquitoes in both the exposure and the control tubes) is made at the end of the specified post-exposure period.

Morphological identification of species and species complexes or groups will be carried out after conducting the insecticide susceptibility tests. Dead adults will be stored and preserved in Eppendorf tubes with silica gel and cotton wool. Molecular identification to species-level of theses specimens will follow.

Molecular identification using PCR (for morphologically identified *An. gambiae* s.l. and *An. funestus* s.l.) will be using to determine specific species insecticide resistance using dead and alive mosquitoes from the insecticide resistance tests. Other Anopheles mosquitoes may be sequenced at the ITS2 or CO1 regions for species identification if required.

10.7 GPS logger movement study

10.7.1 Outcome measure

<u>Outcome 1: Proportion of individual's time spent in different locations (farm, cattle post, village) and temporal movement patterns</u>

The proportion of individual's time spent in different locations will be measured from aggregating GPS readings (located within pre-defined space-time windows) which are spatially located within a given type of area (farm, cattle post, grazing location, village etc). Where possible, common locations will be tagged and classified by field workers to distinguish individual farms, fields and other points of interests (such as bars, homes, etc).

<u>Outcome 2: Distance and frequency of travel during and between agricultural seasons</u>

Aggregated GPS readings (located within pre-defined space-time windows) will be plotted to distinguish individual trips and measure specific characteristics of each trip, including distance of travel and frequency of trips.

10.7.2 Data collection

The study will recruit agricultural workers and cattle herders who meet one of the following criteria in Zambezi Region:

- Prevalent cases identified during the cross-sectional survey (n=20)
- RDT-negative cohort recruited during the cross-sectional survey (n=40)
- Incident cases passively detected during the study period (n=20)
- A cohort of HRPs sampled from AL distribution at T2 (beginning of harvest season)

In Ohangwena Region, the study will recruit cattle herders who meet one of the following criteria:

- Prevalent cases identified during the cross-sectional survey (n=20)
- RDT-negative cohort recruited during the cross-sectional survey (n=40)
- Incident cases passively detected during the study period (n=20)

In addition to the above, eligible individuals must be at least 18 years of age and be willing to wear/bring tracking device for two or more consecutive months. This time period may be longer for cattle herders in Ohangwena, who may not return to Namibia as frequently. All participants will be followed for a minimum of two months from the date of recruitment.

During the enrollment process, the study team will provide detailed information about the data collected with the GPS tracker and how such data will be used for malaria interventions in Namibia. Participants will be asked to give informed consent and allowed 2-3 days to decide whether to participate or not in this study.

Mobile Action i-gotU USB GPS Tracker (GT-100)

Mobile Action Part Number: GT-100

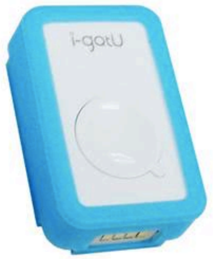

Dimensions:	47 x 29 x 12 mm
Weight:	21g
Power:	Built-in 230mAh Lithium-ion battery
Chipset:	Built-in SiRF StarIII low-power chipset
Antenna:	Built-in GPS patch antenna
Average Acquisition Time:	Cold Start : < 60 seconds<br>Warm Start : < 38 seconds
Interface:	USB 1.1 interface for PC Connection
Operation Temperature:	-10° to + 60° C

Consenting individuals will be asked to wear an "i-gotU" (i-gotU model GT100) GPS logger device continually for a period of two months during all of their activities and to ensure that batteries are refreshed. All enrolled study participants will receive one GPS device, eight replacement batteries and a battery charger. Each battery is expected to last 7-10 days and participants will be requested to charge batteries every Friday/once a week. The spatial accuracy of this device is estimated at roughly 4 meters. The GPS device will be programmed to provide GPS locations every hour over the one-month study period. The device will be programmed to stay turned on at night but will not transmit when the individual is not moving.

The study team will interview study participants at the end of the first and second months, if possible, using a movement questionnaire (Appendix 21) to describe recent travel over the past month, distances,

locations visited and purpose of travel, in order to cross-validate both sets of movement data. In addition, the questionnaire will assess the usability of using the tracking device in terms of comfort carrying it and challenges keeping it charged and on their person. The questionnaire will be administered by trained study staff. Sites identified in interviews will be located and geocoded if they are located within the study area.

10.7.3 Data management and analysis

Raw GPS data will be stored in a password-protected server and all analysis will be conducted by assigned researchers at UCSF.

Trajectories from all study participants will be tagged with the date, time, elevation, latitude and longitude. A spatial algorithm (i-cluster) will be used to aggregate consecutive GPS readings located within pre-defined space-time windows. GPS data will be plotted over a malaria risk surface derived from environmental and vector data, where available, and types of place (bar in a village, small scale farm, cattle post, kraal, etc) to estimate time spent in locations during during biting hours (dusk through dawn). Independent analyses will be carried out to compare the agreement between GPS data collection and the movement interview.

11 Laboratory analysis

The laboratory procedures described below will be followed for all laboratory-based activities conducted during this project. The laboratories performing these tests include UNAM, UCSF and Notre Dame.

Rapid diagnostics tests

Carestart RDTs (CareStart™Malaria HRP2/pLDH Combo Test) will be used to determine malaria infection status and will be performed on participants during cross-sectional surveys, GPS logger follow-ups and to pregnant women (or women of reproductive age refusing pregnancy testing) during presumptive treatment. The HEW and/or nurse will use a finger prick blood sample to run the Carestart RDTs with results available within 20 minutes and recorded by study staff. All RDTs will be used according to the manufacturer's instructions. The results of these tests will be provided to the participant.

RDTs collected at health facilities and by HEWs as part of routine diagnosis for malaria will be collected for further molecular studies for malaria epidemiology only.

Filter paper sample collection

Dried blood spots (DBS) will be collected onto filter paper during cross-sectional surveys for future molecular studies for research purposes only. These results will not be provided to the participants. Filter paper (Whatman 3MM) will be pre-cut into individual squares and stapled to a thick card that will serve as its cover. Blood spots will be collected onto the filter paper in volumes of approximately 25 µl aliquots per blood spot (4 blood spots per card). Filter paper samples labelled with the individual's barcodes or ID number on the covering cardboard and will be allowed to dry at ambient temperature and relative humidity before closing the card over the filter paper. Filter paper samples will be transported from the field in a Ziploc bag then placed in a stock card filter paper box with desiccant and humidity indicator card and stored at 4˚C within one week, and at -20˚C within one month. Dried blood

spots (DBS) will be regularly transported to the district or regional offices for refrigerated storage prior to bulk transport and shipment to designated laboratories.

Molecular testing

Dried blood spots will be extracted using the Chelex method with Tween. Samples will undergo PCR testing with species-specific identification at UNAM. Other molecular studies may include analyses of polymorphisms in parasite and/or human genes for mutations that may affect clinical malaria. Molecular studies will be performed only for research purposes and will have no impact on the clinical management of study participants.

Entomological specimens morphologically identified An. gambiae s.l. and An. funestus s.l. will be identified to specific species using standard published assays using PCR (at UNAM) [21, 22]. Other Anopheles species and those that no not amplify with PCR (other species) may be sequenced at the ITS2 or CO1 regions [23] (at the university of Notre Dame) for species identification if required

Serology

Serology, a test of past infection as assessed by the presence of antimalarial antibodies, will be used to improve the identification of hotspots and estimate current and historical transmission intensities[9]. Using DBS, ELISA assays will be performed using previously described methods. Briefly, antibodies will be eluted from DBS and assayed to detect antibodies against the *P. falciparum* blood stage antigens including merozoite surface protein-1 (MSP-1) and apical membrane antigen-1 (AMA-1), both biomarkers of *P. falciparum* exposure[10]. Other antigens that are sensitive and specific for recent exposure (currently undergoing evaluation) for *P. falciparum* or *P. vivax* may also be used. ELISA assays will be performed in duplicate and optical densities recorded with an ELISA reader. Other serological and antigenic platforms (bead array, protein microarray) may be used to analyze responses to multiple antigens/antibodies, if available. Serology testing will be conducted at UNAM in Namibia, capacity allowing, or be sent to UCSF for processing.

Genotyping

Genotyping of *P. falciparum* will be used to characterize genetic diversity and importation dynamics in the two regions, and ultimately inform network transmission models. A panel of microsatellites located throughout the genome will be genotyped. Briefly, DNA samples will be amplified in a multiplex pre-amplification step followed by amplification of microsatellites in individual reactions using fluorescently tagged primers and sized using denaturing capillary electrophoresis. Multilocus genotypes from mixed infections will be reconstructed, where possible, by quantifying alleles at each locus. Genotyping of additional loci including for HRP2 deletion will be performed as needed.

Individual microsatellite amplifications will be undertaken using single round or nested PCR assays with fluorescently labeled primers, and the amplicons sized by denaturing capillary electrophoresis with internal size standards. Allele-calling will be undertaken with the aid of the GeneMapper v4.0 software. The potential for effective multilocus haplotype reconstruction in polyclonal infections will be explored. Additional informative SNP markers identified in whole genome sequencing efforts may also be genotyped as necessary to improve sample fingerprinting.

Genotyping for markers of drug resistance will include PCR and/or sequencing to identify markers of resistance to artemether-lumefantrine.
Genotyping will be conducted at UCSF, in collaboration with UNAM staff to improve capacity building in this area.

12 Ethical considerations

12.1 IRB

Prior to implementation, the protocol and all related project activities will be reviewed and approved by the institutional review boards in Namibia and UCSF. For other partnering institutions (i.e. University of Texas Southwestern and London School of Hygiene and Tropical Medicine), requests will be made for reliance on UCSF IRB.

12.2 Racial and ethnic origin

A diverse range of ethnic groups will be included in the study, including several marginalized ethnic minorities. Our study will be conducted outside of the U.S., and no racial/ethnic group will be excluded. We do not expect to find race/ethnicity differences in the intervention effect, but refusal rates may differ.

12.3 Inclusion of vulnerable subjects – children and pregnant women

All age groups will be included in this study if they meet eligibility criteria, although children are primarily expected to accompany adults to worksites. All children that participate must have the consent of the parent or guardian. Children older than 12 years and less than 18 must also provide written assent before participation. All women who are pregnant or believe they may be pregnant will be assessed appropriately before treatment.

12.4 Informed consent procedures

Participation in all research and interventions is voluntary. Written informed consent will be obtained from any participant eligible for the study. For the field procedures (survey, blood testing, treatment and follow-up visits), people will be asked to consent each time they are eligible to be part of the study. Inform consent forms are provided in appendices 6a-6l. Consent will not be required for collection of de-identified, used RDTs from health facilities inside and outside of the study areas, as these samples are already collected and available under routine malaria case management activities by the MoHSS. Consent will also not be required for activities that are already implemented to the wider population as part of the MoHSS standard of care, including IRS, LLIN distributions, and reactive screening and treatment. Presumptive treatment is not part of the current standard of care, and the information form will be explained to all individuals at a distribution point (farm or cattle post) prior to obtaining individual informed consent.

Individuals will be included in the study only if they or their parents/guardians provide written informed consent. The consent process will be conducted in a private area and in an appropriate local languages at the start of every new contact with an individual. For individuals under 18 years of age, informed consent will be obtained from a parent or guardian. Written assent for adolescents 12 to <18 years of age will be obtained in addition to consent from a parent or guardian. As part of the informed consent, specific consent for the pregnancy test will also be required for female participants of child-bearing age (15 – 49 years).

As part of the informed consent process, study personnel will assess participants' understanding of the study procedures that were explained by using a checklist comprised of key components of the study. Participants who score at least 80% will be allowed to sign the written consent form. If the participant does not pass, the consent discussion will be repeated, before asking for a signature. In this case, the

consent form will be read again, focusing on areas where understanding was limited, and encouraging the subject to ask questions. If the participant is unable to read or write, an X will substitute for a signature. Informed consent will also be obtained from staff participating in the mosquito human landing catches during the entomological assessment. No women will be allowed to participate in this activity.

12.5 Alternatives to participation

Participation in the research study is voluntary. Individuals electing not to participate in the research study may still receive testing and treatment as part of the routine malaria case management. Individuals who do not wish to receive testing and treatment or presumptive treatment during the intervention campaigns may visit local health facilities for malaria testing and treatment.

12.6 Potential benefits of the proposed research

The proposed research may benefit patients in direct and indirect ways. Participants will directly benefit from the curative effects of AL for low-level parasitemia which may not be detected through conventional malaria testing. Participants will also directly benefit from the protective effect of the malaria prevention package (Targeted IRS, LLIN and Repellent). Furthermore, patients may directly benefit due to community-wide reductions in malaria transmission that are expected to occur after the application of the interventions.

Participants may also indirectly benefit, as the information gained from this research will be used to expand coverage to underserved populations and help establish the safety and efficacy of these strategies in Namibia. The research will benefit the scientific and malaria control communities more generally by expanding the evidence base on targeted delivery of malaria interventions to high risk populations.

12.7 Potential risks of the proposed research

Detailed discussion of potential AEs related to drug regimens is discussed above in Section 9.1.6. Finger pricks for RDTs and DBS are associated with small risks of bleeding, hematoma, and infection. To minimize these risks, the skin will be cleaned with alcohol prior to puncture, and sterile unused lancets will always be used, and pressure will be placed on the puncture site after removal of the lancet using sterile gauze. Although the quantity of blood drawn would not lead to any ill effects on the participants' health, some adults and rarely children feel faint from the blood during the finger prick. The risks will be minimized by having trained health staff perform all procedures, and all untoward effects will be evaluated by health center staff.

The safety risks associated with participation in this study are expected to be minimal. The study drugs, AL and single low dose PQ have been demonstrated to be well tolerated and safe by other studies in Namibia. Prior to drug administration, participants will be asked about known contraindications, and if such contraindications are reported, participants will be restricted from taking the relevant medication. PQ administered as a single low dose has been found to be safe in individuals with any of the G6PD variants and is recommended by WHO without G6PD testing.

The risks associated with loss of privacy in this study are likely to be low. To ensure confidentiality is maintained, all information will be treated as private by study personnel, and records kept securely in locked filing cabinets and offices. Electronic records will be kept on a secure, firewall- and password-protected server. For all data collected as part of the study, participants will be assigned a unique identification number. No personal identification information such as names will be used in any reports arising out of this research. All project staff will be trained on procedures for maintaining confidentiality.

Participants will not be paid to take part in this study. Most assessments will be conducted at working sites or other previously identified gathering points, which will eliminate the need for participant travel and minimize opportunity costs for the participants. Any diagnosis and treatment associated with the study will be provided free of charge.

12.8 Adequacy of protection against risks

We will administer an informed consent form both verbally and in writing to all participants in the local language for participation in all study activities. These forms will be read or will be given to participants to read themselves and will include a full description of voluntary participation, the right to withdraw from the study at any time, and the right to not answer any question or participate in any component of the research.

These forms will also address the risks, benefits, and purpose of the study and what we hope to learn. We will train all interviewers extensively on the consent procedure, and each form will be co-signed by a team member to ensure all participants have consented. Checks in the field by the PI and project leaders will further ensure the consent process is followed in all cases. Data collection team members will provide the contact information for study coordinators who can be contacted for any further information on the topics brought up in the interview, or for additional treatment if necessary. The confidentiality procedures are designed to meet all contingencies to ensure the confidentiality of participant data and the privacy of the participants is preserved.

Our proposed strategies to reduce risks to privacy or of disclosure of confidential information include:

1. Identifying information will be recorded only in secure database software on password protected computers, and data collectors will only have access to the data that they themselves directly collect which will be cleared from their devices after all follow-up visits are completed. All data will be stored only in password-protected files on password-protected computers in locked offices.
2. Prior to analysis, data will be de-identified with the exception of geo-location codes, which are necessary for specific per-protocol analyses. The absence of individual identifying information will protect subject confidentiality.
3. All paper records will be stored in a locked location.

The potential risks of drawing blood from a finger-prick include temporary discomfort, pain, transient bleeding, bruising, skin infection, and fainting. The volumes of blood taken will be too small to produce any adverse physiologic effects from blood loss anemia and overall the aforementioned risks associated with blood draws are likely to be low. Study staff will be trained in the proper conduct of a finger-prick according to standard operating procedures to minimize the risk of discomfort and infection.

12.9 Management of sick participants
During the intervention, people with complicated malaria or symptoms requiring further evaluation will be referred for evaluation at health facility.

12.10 Data and safety monitoring plan

Adverse events will be identified, reported and managed as indicated in Section 9.1.6. Due to the fact that this study is expected to accrue too quickly to allow for a Data Safety and Monitoring Board (DSMB) to be constituted and complete data and safety monitoring, and since the local investigator will have access to all data, a DSMB may not be feasible, instead an independent local safety monitor will be appointed to evaluate adverse events, assess associations and severity and make recommendations for continuing or stopping the treatment.

For the purpose of this study, the terms “Adverse Event” (AE) and “Serious Adverse Event” (SAE) will have the meanings below:

A. Adverse Event (AE)
Any untoward medical occurrence in a participant who is receiving or has received drugs (artemether-lumefantrine, or single low-dose primaquine). An adverse event can be any unfavourable and unintended sign (including an abnormal laboratory finding), symptom, or disease temporally associated with the use of the study drug(s). It may or may not be causally related to study drug.
Whereas the word ‘severe’ refers to the intensity of the adverse event and is graded. The term ‘serious” (e.g. serious adverse event, or SAE) is not graded. The functional table below (Table 8) will be used to grade the severity of an AE.

Table 8. Functional table to grade AE severity

Grade 1 AE - Mild	Grades 2 AE - Moderate	Grade 3 AE - Severe	Grade 4 AE –Potentially life-threatening or resulted in death.
An event that is easily tolerated by study participant, causing minimal discomfort and not interfering with everyday activities. The event can generally be managed at home.	An event that is sufficiently discomforting to interfere with everyday activities. An event resulting in an outpatient visit (e.g. local clinic) is generally but not always considered at least Grade 2.	An event that prevents normal everyday activities. An event resulting in hospitalization is generally but not always considered at least Grade 3.	Potentially life-threatening symptoms causing inability to perform basic self-care functions with intervention indicated to prevent permanent impairment, persistent disability, or death. An event resulting in death is also Grade 4, although some agencies have defined a final category, Grade 5, strictly for deaths due to an AE.

These suggestions will not be regarded rigidly, each reported AE will be dealt on its own merit, exercising the expected high standards of medical care and discretion.

B. Serious Adverse Event (SAE)
An SAE is any untoward medical occurrence that:

Results in death, OR

b) Is life-threatening,
OR

c) Requires hospitalization or prolongation of existing hospitalization,
OR

d) Results in disability/incapacity,
OR

e) Is a congenital anomaly/birth defect,
OR

f) Medical or scientific judgment should be exercised in deciding whether reporting is appropriate in other situations, such as important medical events that may not be immediately life-threatening or result in death or hospitalisation but may jeopardize the subject or may require medical or surgical intervention to prevent one of the other outcomes listed in the above definition. These should also be considered serious. Examples of such events are invasive or malignant cancers, intensive treatment in an emergency room or at home for allergic bronchospasm, blood dyscrasias or convulsions that do not result in hospitalisation, or development of drug dependency or drug abuse.

13 Potential limitations, data quality assurance, and dissemination plan

13.1 Potential limitations

Several limitations have the potential to compromise study outcomes.

<u>Malaria declines</u>
Compared to 2016-2018, transmission in 2019 was substantially lower. Large-scale declines in malaria incidence and prevalence throughout the study area over the 2019-2020 transmission season could compromise study power.

<u>Malaria increases</u>
Any large increases in malaria burden in target regions could increase overall caseloads at health facilities, potentially impacting availability of HEWs or other staff to support study activities and interventions.

<u>Differences in timing and approaches between regions</u>
Formative research in Zambezi and Ohangwena Regions have illustrated differing mobility patterns which requires cross-sectional surveys and implementation of interventions to be implemented differently. Specifically, cattle herders in Ohangwena Region are expected to be grazing cattle in Angola from August until the rainy season arrives, with some cattle herders not returning until June when the

cattle are brought back to graze on the leftover harvest. As a consequence, baseline surveys and LLIN distributions will be closely coordinate with cattle owners in order to maximize coverage of cattle herders coming from Angola. This population will be presumptively treated on their return to Namibia. In contrast, HRPs in Zambezi Region work at venues in the region and can be accessed throughout the transmission season during peak agricultural seasons.

<u>Changes in mobility of target populations</u>
The cross-border mobility patterns of HRPs targeted in this intervention are largely dictated by agricultural productivity and rainfall patterns. Low rainfall and poor crop yield (or poor gazing potential) will reduce demand for labor within the region and will affect population estimates as well as risk of infection. In addition, HRPs may freely transit between intervention and non-intervention HFCAs in order to seek work at different sites, and thereby 'carry-over' community-led interventions. The use of unique IDs will allow the impact of these movements to be assessed and adjusted for in exploratory analyses.

<u>Other partners' interventions impact study outcomes</u>
The implementation of vector control activities and/or mass screen and treat programs by other partners is possible, particularly in the event of a malaria outbreak in the region. These activities have the potential to impact study outcomes if implementation is differential across the study arms. A detailed matrix of other project activities will be created as well as data collection around participation in other activities during the baseline and endline cross-sectional surveys; these inputs will be used to assess and adjust outcomes for exploratory analyses. In addition, routine programmatic data for IRS, LLIN distribution (based on case investigation forms and ANC reports) and RACD will be collated at the health facility level to adjust for differences in control and intervention areas in the analysis.

13.2 Data quality assurance plan

<u>Data quality and management</u>
Data collection will occur in multiple locations within two regions, and differences in implementation could potentially bias results. However, study teams and regular health staff will receive comprehensive training, as well as ongoing evaluation, supervision, and supplementary capacity building as necessary to ensure data quality and completeness.

<u>Procedures to minimize biases</u>
All survey instruments and data collections will be based on the formative work (carried out in November-December 2018) and previous studies in the region. These tools will be developed in English, translated to Silozi and Oshikwanyama, and then back-translated before field testing. Any other languages (such as Oshivale) will be conducted in the appropriate language, and translated into one of the two languages above. A pilot study will test the utility of the survey instruments; these data will then be discarded if significant changes are made to the survey instrument. Study coordinators will be responsible for monitoring data quality to ensure that questionnaires are completed and entered correctly.

<u>Potential changes to this protocol based on the piloting of tools and methods</u>
The organization and supervision of HEWs may be changed based on initial feasibility studies during field-testing of survey instruments.

13.3 Dissemination Plan

The results of the cross-sectional surveys and intervention coverage statistics will be shared on a real-time basis with national and regional-level partners as well as other key stakeholders (i.e., WHO, CHAI,

Isdell Flowers) throughout implementation to ensure that the most up-to-date information about malaria is available in the regions.

14 Timeline

Expected outcomes and related activities	2019								2020							Milestones
	5	6	7	8	9	10	11	12	1	2	3	4	5	6	7	
**Study preparation**																• Develop, review and finalize work plan and protocol. In-country review; incorporate feedback into protocol. Finalize protocol.<br>• Sensitize participating health catchment areas<br>• Complete and submit applications to IRBs<br>• Finalize & translate informed consents<br>• Finalize questionnaires
**Procurement**																• Finalize SOPs for field procedures<br>• Order supplies (drugs, insecticide, field supplies)
**Trainings**																• Finalize & translate training materials<br>• Conduct trainings for field personnel
**Interventions**																
**Mop up IRS & LLIN**																• Field team deploys IRS in intervention areas<br>• Field team distributes LLINs/vector control packs to target populations
**Presumptive treatment**																• Field team distributes AL to target populations in intervention areas
**Adverse event monitoring**																• When adverse events occur, collecting additional data to assess causality<br>• Prepare reports of serious adverse events
**Data collection**																
- **Mapping and enumeration**																• Field team conducts physical mapping and enumeration in intervention areas with mop-up IRS activities
- **Baseline Cross-sectional**																• Data collection at baseline in OHA (immunization clinic and water points) and ZAM (ploughing season), coordinated through gatekeepers
- **Case-control study**																• Ongoing collection of data from incident cases and matched controls meeting HRP definition in intervention areas
- **Endline cross-sectional**																• Data collection at endline in OHA (festive bull competition and water points) and ZAM (harvest season), coordinated through gatekeepers
- **Qualitative**																• Field team conducts IDI and FGDs with HRPs and key stakeholders
**Analysis**																
**Laboratory assessment**																• finalize SOPs for laboratory procedures<br>• Conduct trainings for laboratory personnel
**Preliminary data analysis**																• Develop and finalize SOP for data analysis<br>• Preliminary analysis on coverage, acceptability, cost-effectiveness, safety and incidence

blue = Zambezi Region, orange = Ohangwena Region

15 References

1. Jacobson JO, Cueto C, Smith JL, Hwang J, Gosling R, Bennett A. Surveillance and response for high-risk populations: what can malaria elimination programmes learn from the experience of HIV? Malaria journal. 2017;16(1):33-. doi: 10.1186/s12936-017-1679-1. PubMed PMID: 28100237.
2. Hlaing T, Wai KT, Oo T, Sint N, Min T, Myar S, et al. Mobility dynamics of migrant workers and their socio-behavioral parameters related to malaria in Tier II, Artemisinin Resistance Containment Zone, Myanmar. BMC Public Health. 2015;15:886-. doi: 10.1186/s12889-015-2241-0. PubMed PMID: 26370297.
3. Smith JL, Ghimire P, Rijal KR, Maglior A, Hollis S, Andrade-Pacheco R, et al. Designing malaria surveillance strategies for mobile and migrant populations in Nepal: a mixed-methods study. Malaria journal. 2019;18(1):158-. doi: 10.1186/s12936-019-2791-1. PubMed PMID: 31053075.
4. IOM (International Organization for Migration). A global report on population mobility and malaria: moving towards elimination with migration in mind. 2013.
5. Naing C, Whittaker MA, Tanner M. Inter-sectoral approaches for the prevention and control of malaria among the mobile and migrant populations: a scoping review. Malaria journal. 2018;17(1):430-. doi: 10.1186/s12936-018-2562-4. PubMed PMID: 30445959.
6. Smith JL, Auala J, Haindongo E, Uusiku P, Gosling R, Kleinschmidt I, et al. Malaria risk in young male travellers but local transmission persists: a case-control study in low transmission Namibia. Malaria journal. 2017;16(1):70-. doi: 10.1186/s12936-017-1719-x. PubMed PMID: 28187770.
7. Malaria Elimination Initiative. Planning for targeted malaria interventions in high-risk populations (HRPs) in Northern Namibia: Findings from a rapid formative assessment in Ohangwena Region. University of California, San Francisco, 2019.
8. Malaria Elimination Initiative. Planning for targeted malaria interventions in high-risk populations (HRPs) in Northern Namibia: Findings from a rapid formative assessment in Zambezi Region. University of California, San Francisco, 2019.
9. Iitula I, Katokele S, Maliti D, Mwema T, Joseph R, Walusimbi D, et al., editors. Enhanced Entomological Surveillance For Malaria Elimination in Namibia: An Initial Baseline Survey. American Society of Tropical Medicine and Hygiene; 2018; New Orleans, USA.: Poster.
10. Wu L, van den Hoogen LL, Slater H, Walker PGT, Ghani AC, Drakeley CJ, et al. Comparison of diagnostics for the detection of asymptomatic Plasmodium falciparum infections to inform control and elimination strategies. Nature. 2015;528:S86. doi: 10.1038/nature16039 https://www.nature.com/articles/nature16039#supplementary-information.
11. The Global Health Group. Screen and treat strategies for malaria elimination: a review of evidence. 2018.
12. Zani B, Gathu M, Donegan S, Olliaro PL, Sinclair D. Dihydroartemisinin-piperaquine for treating uncomplicated Plasmodium falciparum malaria. The Cochrane database of systematic reviews. 2014;(1):Cd010927. Epub 2014/01/21. doi: 10.1002/14651858.cd010927. PubMed PMID: 24443033; PubMed Central PMCID: PMCPMC4470355.
13. Kern SE, Tiono AB, Makanga M, Gbadoe AD, Premji Z, Gaye O, et al. Community screening and treatment of asymptomatic carriers of Plasmodium falciparum with artemether-lumefantrine to reduce malaria disease burden: a modelling and simulation analysis. Malaria journal. 2011;10:210. Epub 2011/08/02. doi: 10.1186/1475-2875-10-210. PubMed PMID: 21801345; PubMed Central PMCID: PMCPMC3161019.
14. Hsiang M, Ntuku H, Roberts K, Dufour M-s, Whittemor B, Tambo M, et al. The effectiveness of malaria reactive focal mass drug administration (rfMDA) and reactive vector control (RAVC), a cluster-

randomised controlled two-by-two factorial design trial from the low-endemic setting of Namibi. 2019. Unpublished.
15. Deardorff KV, Rubin Means A, Ásbjörnsdóttir KH, Walson J. Strategies to improve treatment coverage in community-based public health programs: A systematic review of the literature. PLoS neglected tropical diseases. 2018;12(2):e0006211-e. doi: 10.1371/journal.pntd.0006211. PubMed PMID: 29420534.
16. Ministry of Health and Social Services. Namibia Malaria Outbreak 2017: Entomological Surveillance Response. Windhoek: Ministry of Health and Social Services, 2017.
17. Initiative ME. Planning for targeted malaria interventions in high-risk populations (HRPs) in Northern Namibia: Findings from a rapid formative assessment in Zambezi Region. University of California, San Francisco, 2019.
18. Banek K, Webb EL, Smith SJ, Chandramohan D, Staedke SG. Adherence to treatment with artemether-lumefantrine or amodiaquine-artesunate for uncomplicated malaria in children in Sierra Leone: a randomized trial. Malaria journal. 2018;17(1):222. Epub 2018/06/06. doi: 10.1186/s12936-018-2370-x. PubMed PMID: 29866192; PubMed Central PMCID: PMCPMC5987409.
19. Gillies MT, De Meillon B. The Anophelinae of Africa south of the Sahara (Ethiopian Zoogeographical Region): Johannesburg: South African Institute for Medical Research, P.O. Box 1038, S. Africa.; 1968. 343 pp. p.
20. WHO. Test procedures for insecticide resistance monitoring in malaria vector mosquitoes. Geneva, Switzerland: World Health Organization, 2016.
21. Scott JA, Brogdon WG, Collins FH. Identification of single specimens of the Anopheles gambiae complex by the polymerase chain reaction. The American Journal of Tropical Medicine and Hygiene. 1993;49(4):520-9. Epub 1993/10/01. doi: 10.4269/ajtmh.1993.49.520. PubMed PMID: 8214283.
22. Koekemoer LL, Kamau L, Hunt RH, Coetzee M. A cocktail polymerase chain reaction assay to identify members of the Anopheles funestus (Diptera: Culicidae) group. The American Journal of Tropical Medicine and Hygiene. 2002;66(6):804-11. Epub 2002/09/13. doi: 10.4269/ajtmh.2002.66.804. PubMed PMID: 12224596.
23. Lobo NF, St Laurent B, Sikaala CH, Hamainza B, Chanda J, Chinula D, et al. Unexpected diversity of Anopheles species in Eastern Zambia: implications for evaluating vector behavior and interventions using molecular tools. Scientific reports. 2015;5:17952. Epub 2015/12/10. doi: 10.1038/srep17952. PubMed PMID: 26648001; PubMed Central PMCID: PMCPMC4673690.

16 Appendices

1. AL distribution form
2. AL weight-based dosing
3. Pill count form
4. RACD form
5. Primaquine dosing chart
6. Informed Consent Forms:
    a. Baseline and Endline Cross Sectional Survey – Adult/Appropriate Guardian - Consent
    b. Baseline and Endline Cross Sectional Survey – Youth Assent (12-17)
    c. Case Control Study – Adult/Appropriate Guardian - Consent
    d. Case Control Study – Youth Assent (12-17)
    e. Presumptive Treatment – Adult/Appropriate Guardian - Consent
    f. Presumptive Treatment - Youth Assent (12-17)
    g. Movement Tracking – Adult - Consent
    h. HLC and HBO Household Participation – Adult - Consent
    i. PSC Household Participation – Adult - Consent
    j. HLC and HBO Data Collectors – Adult - Consent
    k. Acceptability Endline KII and FGD – Adult - Consent
    l. Acceptability HEW Interviews – Adult - Consent
7. Baseline/End line Cross Sectional Survey Questionnaires
8. Case Control Study Questionnaire
9. Entrance, Mid-intervention and Exit HEW Questionnaire
10. Acceptability HEW and Health Workers KII Interview Guide
11. Acceptability HEW and Health Workers FGD Interview Guide
12. Acceptability Employers and Owners KII Interview Guide
13. Acceptability Employers and Owners FGD Interview Guide
14. Acceptability HRP group member FGD Interview Guide
15. Vector Control Pack (LLINs, Repellent) Distribution Form
16. Topical repellant Use Log
17. Mosquito Net Use Log
18. HLC Field Collection Form
19. Human Behavior Field Data Collection Form
20. PSC Adult Mosquitoes Field Collection Form
21. GPS logger questionnaire
